# Efficient Regioselective Iodination of Pyrazole Derivatives Mediated by Cadmium(II) Acetate

**DOI:** 10.1002/open.202400443

**Published:** 2025-02-28

**Authors:** Nina G. Hobosyan, Kristine V. Balyan, Lusine A. Movsisyan, Varduhi S. Hovsepyan, Armen G. Ayvazyan, Henrik A. Panosyan, Hovhannes S. Attaryan, Hmayak B. Sargsyan, Haykanush R. Pogosyan

**Affiliations:** ^1^ Department of Organic Chemistry Scientific Technological Center of Organic and Pharmaceutical Chemistry National Academy of Sciences of the Republic of Armenia 26 Azatutyan Ave. Yerevan Republic of Armenia; ^2^ Department of Pharmaceutical Chemistry International Scientific Educational Center of NAS RA 24 M. Baghramyan Ave. Yerevan Republic of Armenia; ^3^ Department of Biology and Chemistry and their Teaching Methodology Armenian State Pedagogical University after Khachatur Abovyan 17 Tigran Mets Ave. Yerevan Republic of Armenia; ^4^ Department of Pharmaceutical Chemistry Yerevan Haybusak University 6 Abelyan Str. Yerevan Republic of Armenia

**Keywords:** iodopyrazole derivatives, iodination, propargylic compounds, pyrazole ring, cadmium (II) acetate

## Abstract

The present study focuses on the iodination of pyrazoles mediated by cadmium (II) acetate. The objects of research were compounds with pyrazole rings substituted by N‐propargyl, C, N‐alkyl groups. Depending on the molar ratios of the reagents effective ways of obtaining mono‐ and triiodo‐substituted products with the participation of the propargylic fragment in DMSO were elucidated. Induced by the cadmium (II) acetate principles of electrophilic iodination of the C‐4 position of the pyrazole ring containing electron‐donating groups were revealed. The iodination of the pyrazole derivative with only propargylic substituent was found to target the CH‐acidic center of the triple bond and to lead to the corresponding iodoalkyne. An iodo‐substituted product with triple bond is more reactive and prone to further electrophilic iodination forming a triiodo‐substituted derivative. The introduction of methyl groups in the pyrazole ring of derivatives with propargylic substituent contributed to the promotion of competitive iodination reactions due to the increase in nucleophilicity of the pyrazole ring. Dimerized pyrazole rings with carbon atoms in the 4‐th position did not succeed in being iodinated by the mentioned way. Possible pathways for both triple bond and pyrazole ring iodination involving acetyl hypoiodite were proposed.

## Graphical abstract

The iodination reactions of both N, C alkyl‐, propargylic substituted and unsubstituted pyrazole derivatives initiated by cadmium (II) acetate were performed. It was revealed that the C‐4 position of the pyrazole ring was more nucleophilic. The nucleophilicity of the pyrazole ring significantly increased due to the influence of alkyl groups and decreased in the case of N‐propargyl substituent.

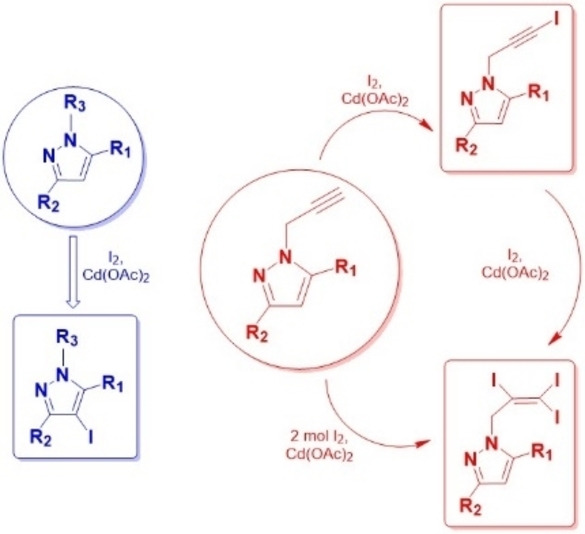

## Introduction

Pyrazoles^[1]^ are known as versatile scaffolds in organic synthesis and medicinal chemistry and often used as starting materials for the preparation of more complex heterocyclic systems with relevance in the pharmaceutical^[2]^ CropScience and other research fields. Numerous substituted pyrazoles are being developed in a wide range of therapeutical areas including anti‐inflammatory, anticancer, antibacterial, analgesic, CNS, endothelin‐antagonists as well as in agrochemistry to obtain herbicides and pesticides. Pyrazole moieties substituted at the 4‐position are often detected in biologically active compounds. 4‐Iodopyrazoles, which possess a useful functional handle at the 4‐position, represent valuable intermediates toward the syntheses of these targets.^[3]^ For example, these compounds have been employed as participants in cross‐coupling chemistry and for halogen‐metal exchange reactions. Unfortunately, existing methodologies for the synthesis of 4‐iodopyrazoles are not ideal for large scale processing.

Many examples of iodination of the pyrazole ring with the participation of oxidants are known in literature. Due to increasing interest in synthetic applications of peroxodisulfate ion, Iranian research group^[4]^ has been exploring its potential use in carbon‐heteroatom bond formation. They have revealed a procedure for the synthesis of 4‐iodopyrazoles directly from pyrazoles using iodine mediated by n‐butyltriphenylphosphonium peroxodisulfate [(n‐BuPPh_3_)_2_S_2_O_8_].

The authors^[5]^ have developed a green procedure for the regioselective 4‐iodination of pyrazoles using only 0.5 equivalent of iodine and 0.6 equivalent of hydrogen peroxide in water. This protocol has been successful for a series of differentially substituted pyrazole starting materials to afford the corresponding 4‐iodopyrazole products in good to excellent yields. We think the disadvantage of current protocol is mainly the application of soluble pyrazoles in water as the substrates. Moreover a convenient and economical one‐pot syntheses of series of new 1‐aryl‐substituted 4‐iodopyrazole‐3‐ols via aromatisation and oxidative iodination reactions from easily available pyrazolidine‐3‐ones in the presence of both sodium iodide and 30 % solution of hydrogen peroxide and a catalytic amount of sulfuric acid in dichloromethane are described in.^[6]^ A facile, sustainable and eco‐friendly protocol has been developed for the halogenation of various heterocycles which afforded the products in 90–95 % isolated yields using TBAHal/Oxone as halogenating agent. The gram‐scale iodination reaction, which demonstrates its potential applicability in organic synthesis, has also been successfully performed by optimizing conventional heating conditions. Further these halogenated pyrazoles have been utilized for different coupling reactions including formation of arylated, alkynylated and sulfenated pyrazoles.^[7]^ Besides, the iodination of N−H or N‐benzylpyrazoles using elemental iodine in the presence of ceric ammonium nitrate (CAN) as *in situ* oxidant is a mild and efficient method to prepare 4‐iodopyrazoles containing even electron‐withdrawing substituents.^[8]^ The reaction is regioselective and the iodine atom attacked 4‐C position of pyrazole ring instead of the benzyl group. The oxidative iodination of pyrazoles using iodine and CAN generates a large amount of cerium waste that is difficult to remove. A synthesis of 4‐iodosubstituted pyrazoles by iodination of pyrazole and its derivatives in the heterophase (H_2_O/CHCl_3_ (CCl_4_)) medium with the system KI/KIO_3_ in the presence of H_2_SO_4_ additives was accomplished.^[9]^ The electrochemical methods of obtaining 4‐iodopyrazols are also known.^[10]^ In addition, iodination reactions proceed in the presence of N‐iodosuccinimide (NIS). For example, dissolution of NIS in sulfuric acid gives rise to electrophilic iodine‐containing species which are capable of successfully iodinating aromatic compounds with electron‐withdrawing substituents in the temperature range from 0 °C to 20 °C. The iodination in sulfuric acid is effected by both protonated NIS and IOS(O)(OH^+^)OH intermediate.^[11]^ On the other hand a new halogenation technique was developed by Brazilian research group^[12]^ which consisted of the formation of an *in situ* iodine trifluoroacetate species from the mixture of trifluoroacetic acid and NIS, which could act as a very reactive electrophile allowing the iodination of even very deactivated pyrazoles in a short reaction time and at satisfactory yields. Iodination reactions preceded by various conversions (for example‐dehydration, aromatization) are also described in the literature. A number of new functionally substituted 1‐acyl‐5‐hydroxy‐4,5‐dihydro‐1*H*‐pyrazoles^[13]^ have been prepared in moderate to excellent yields from the corresponding 2‐alkyn‐1‐ones. The resulting dihydropyrazoles undergo dehydration and iodination in the presence of ICl and Li_2_CO_3_ at room temperature to produce 1‐acyl‐4‐iodo‐1*H*‐pyrazoles. Keipour'steam^[14]^ has suggested an original method for iodination of pyrazolic derivatives and other aromatic compounds using potassium ferrate supported on montmorillonite.

Therefore, several articles on the chemistry of pyrazoles have been published. However, they focus mainly on their synthesis and applications leaving aside the reactivity component representative of pyrazoles. Thus, in this manuscript we will focus on the pyrazole moiety, chemistry and reactivity toward electrophilic iodination. In the literature collected by us there are no examples of iodination reactions carried out in the presence of cadmium (II) acetate. The lack of similar data prompted to direct the group‘s further studies to the exposing of iodination reaction principles of electron‐rich pyrazole compounds.

## Results and Discussion

**Figure 1:**
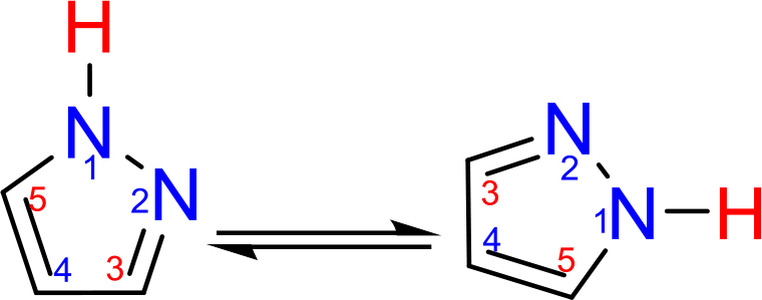
Pyrazole structure highlighting the nucleophilic (blue) and electrophilic (red) positions.

Pyrazoles are known to be electron‐rich heterocyclic systems and can be widely used in organic syntheses due to their diverse chemical properties. The three positions of the pyrazole skeleton‐N1, N2, C4 are nucleophilic, and the C3, C5 positions are electrophilic (Figure 1.^[15]^).

**Scheme 1:**
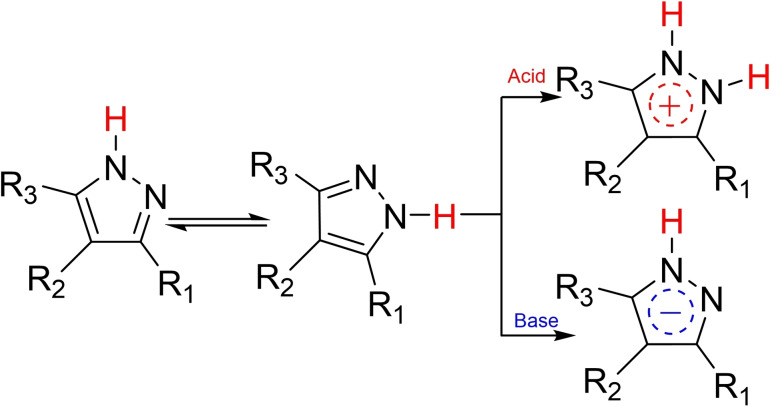
Schematic representation of the chemical behavior of pyrazoles in acid and basic media.^[15]^

In addition, depending on the reaction conditions, the functionalization of the pyrazole ring can be carried out both with nucleophiles (at C‐3 and C‐5 positions) and electrophiles (at C‐4 position and/or with the participation of any nitrogen atoms). Among the pyrrole and pyridine nitrogen atoms of the pyrazole ring the pyridine nitrogen has the nucleophilic property.^[15]^ As it is known, the nucleophilicity of the pyrazole ring changes significantly in the protonation or deprotonation of pyrazoles due to the amphoteric nature of pyrazole (Scheme 1).

In our previous works, a new way of iodination of propargylic derivatives mediated by cadmium(II) acetate in polar protic and aprotic solvents had been described.^[16]^ We found it more convenient to direct further studies to the sphere of N‐propargyl substituted pyrazole derivatives, because in the works^[16]^ the research had been done with the participation of propargylic systems. Based on the presented works, pyrazole derivatives containing various substituents were selected as the target substrates.

**Scheme 2:**
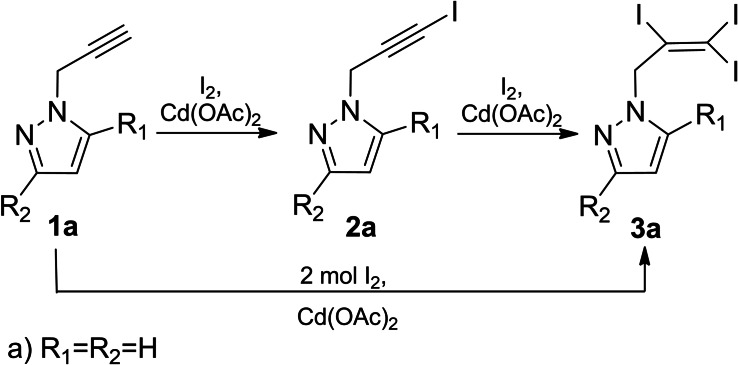
Iodination of propynylpyrazole derivatives **1 a**, **2 a**.

We started the study of the iodination reaction in the presence of cadmium (II) acetate with the model compound 1‐(prop‐2‐yn‐1‐yl)‐1*H*‐pyrazole, where the propargylic group was optimally combined with the pyrazole ring **(**Scheme 2).

**Figure 2:**
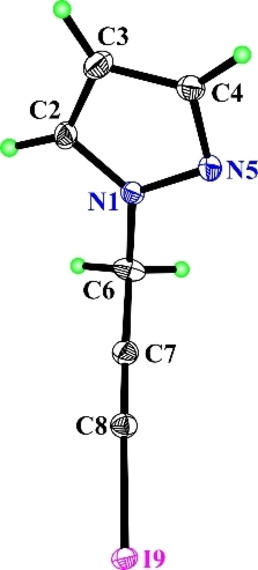
X‐ray crystallographic structures of **2 a**.^[17]^

The iodination was carried out at room temperature in the presence of polar aprotic (DMSO), protic solvents (methanol, ethanol). It became clear that the iodination of 1‐(prop‐2‐yn‐1‐yl)‐1*H*‐pyrazole (**1 a**) with 1/1/1 quantitative ratio of substrate/iodine/cadmium(II) acetate in DMSO proceeded efficiently leading to 1‐(3‐iodoprop‐2‐yn‐1‐yl)‐1*H*‐pyrazole (in 70 % yield) (**2 a**) (Figure 2).

However, the iodination of the same substrate with a molar ratio of substrate/iodine/cadmium (II) acetate 1/2/1, was accompanied with the formation of triiodosubstituted derivative‐ 1‐(2,3,3‐triiodoallyl)‐1*H*‐pyrazole (**3 a**, in 55 % yield). It should be noted that 1‐(2,3,3‐triiodoallyl)‐1*H*‐pyrazole (**3 a**, in 75 % yield) was also obtained from the iodination of 1‐(3‐iodoprop‐2‐yn‐1‐yl)‐1*H*‐pyrazole^[16b]^ (**2 a**). The structure of the obtained compounds was validated by ^1^H, ^13^C NMR, HRMS and X‐ray structural analysis data.^[17]^ The molecular structure of compound **2 a** is shown in Figure 2.

CCDC 2383957 (for **2 a**) https://www.ccdc.cam.ac.uk/services/structures?id=doi:10.1002/open.202400443 contains the supplementary crystallographic data for this paper. These data are provided free of charge by the joint Cambridge Crystallographic Data Centre and Fachinformationszentrum Karlsruhe, Access Structures service, http://www.ccdc.cam.ac.uk/structures.

Based on the previous works the proposed mechanism of iodination initiated by cadmium acetate is also applicable in elucidating the principles of iodination of **1 a** used in this work. Thus, sources of electrophilic iodine can be considered both the active intermediate acetyl hypoiodite (IOAc) and molecular iodine, electrophilicity of which increases as a result of polarizing with cadmium (II) acetate.

**Scheme 3:**
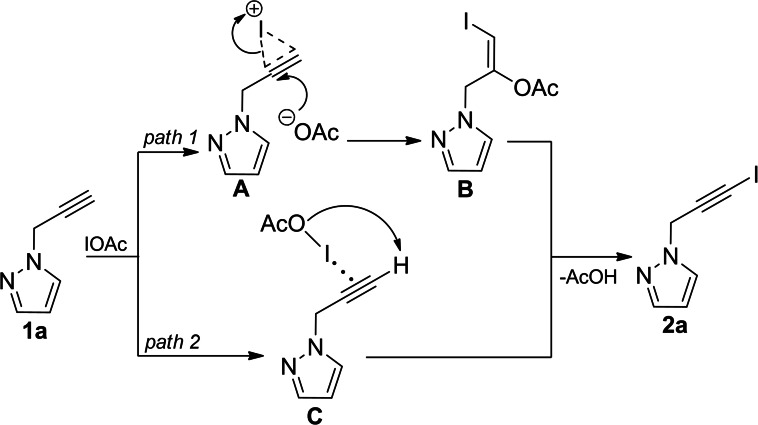
Plausible mechanism of iodination of propynylpyrazole with the acetyl hypoiodite.

The interaction of acetyl hypoiodite with the propargyl fragment of the pyrazole derivative can be presented by two possible directions with the formation of iodonium complex **A** (*path 1*) transformed into iodoacetate intermediate **B** and π‐coordinated particles **C** (*path 2*). Subsequent stabilization of the above mentioned intermediates by the elimination of acetic acid leads to **2 a** (Scheme 3).

**Scheme 4:**
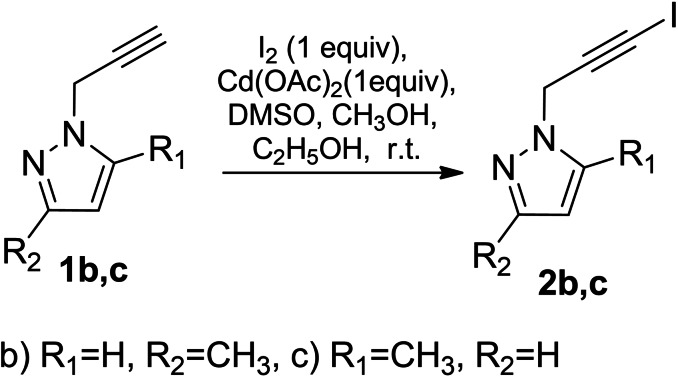
Iodination of propynylpyrazole derivatives **1 b**, **1 c**.

However, appropriately replacing propargylpyrazole **1 a** by 3‐methyl‐1‐(prop‐2‐yn‐1‐yl)‐1*H*‐pyrazole (**1 b**) or 5‐methyl‐1‐(prop‐2‐yn‐1‐yl)‐1*H*‐pyrazole (**1 c**) the regioselectivity of iodination reaction changes depending on the molar ratio of the reactants. In the case when the molar ratio of substrate/iodine/cadmium(II) acetate is 1 : 1 : 1 only the triple bond is targeted for iodination respectively leading to 1‐(3‐iodoprop‐2‐yn‐1‐yl)‐3‐methyl‐1*H*‐pyrazole^[17]^ (**2 b**) and 1‐(3‐iodoprop‐2‐yn‐1‐yl)‐5‐methyl‐1*H*‐pyrazole (**2 c**) (Scheme 4).

**Figure 3:**
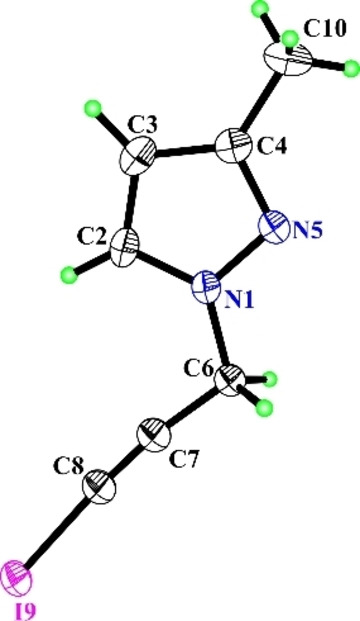
X‐ray crystallographic structures of **2 b**.^[17]^

The structure of the obtained compounds was validated by ^1^H,^13^C NMR, HRMS data. The molecular structure of compound **2 b** is shown in Figure 3.

CCDC 2383965 (for **2 b**) https://www.ccdc.cam.ac.uk/services/structures?id=doi:10.1002/open.202400443 contains the supplementary crystallographic data for this paper. These data are provided free of charge by the joint Cambridge Crystallographic Data Centre and Fachinformationszentrum Karlsruhe, Access Structures service, http://www.ccdc.cam.ac.uk/structures.

**Scheme 5:**
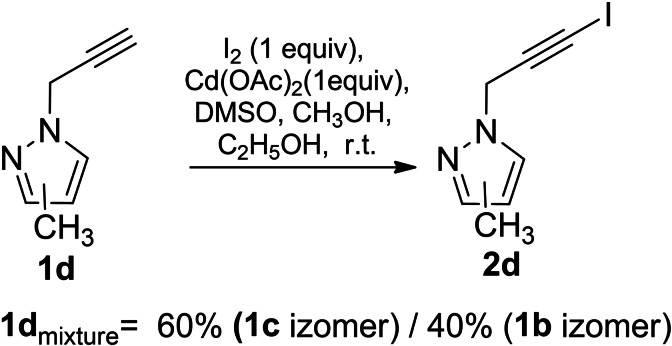
Iodination of propynylpyrazole derivative **1 d**.

The iodination reaction was also explored on the sample of a mixture of 3(*5*)‐methyl‐1‐(prop‐2‐yn‐1‐yl)‐1*H*‐pyrazoles (**1 d**) (60(**1 c**)/40(**1 b**) % molar ratio). According to ^1^H NMR data, the iodination proceeded again via the substitution of the hydrogen atom of the triple bond, leading to a mixture of 1‐(3‐iodoprop‐2‐yn‐1‐yl)‐3(*5*)‐methyl‐1*H*‐pyrazoles (**2 d)** which molar ratio coincided with the starting materials **1 d** (Scheme 5).

**Scheme 6:**
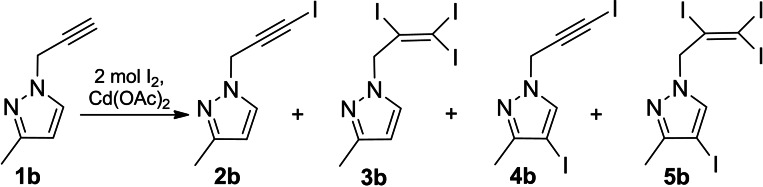
Iodination of propynylpyrazole derivative **1 b** with double excess of iodine

In the case of applying double excess of iodine upon 3‐methyl‐1‐(prop‐2‐yn‐1‐yl)‐1*H*‐pyrazole (**1 b**) both the triple bond and the C‐4 position of the pyrazole ring were subjected to electrophilic attack resulting in a mixture of the following four substances 1‐(3‐iodoprop‐2‐yn‐1‐yl)‐3‐methyl‐1*H*‐pyrazole (**2 b)**, 3‐methyl‐1‐(2,3,3‐triiodoallyl)‐1*H*‐pyrazole (**3 b)**, 4‐iodo‐1‐(3‐iodoprop‐2‐yn‐1‐yl)‐3‐methyl‐1*H*‐pyrazole (**4 b**) and 4‐iodo‐3‐methyl‐1‐(2,3,3‐triiodoallyl)‐1*H*‐pyrazole (**5 b**) (Scheme 6).

The basis for the identification of the mixture was the data of ^1^H, ^13^C NMR (Table 1, Table 2). According to the ^1^H NMR data (Table 1) of the experimental result the iodo‐substituted product of the triple bond **2 b** dominated in the mixture of **2 b**, **3 b**, **4 b**, **5 b**.

**Table 1:** ^1^H NMR data (ppm) of the components **2 b**, **3 b**, **4 b**, **5 b** of the mixture.

N ppm(mult.*)	5‐CH‐Pz	4‐CH‐Pz	CH2	CH3
2b	7.43 d	6.05 d	5.00 s	2.27 s
3b	7.32 d	6.07 d	4.98 s	2.29 s
4b	7.52 s	‐	4.99 s	2.23 s
5b	7.42 s	‐	4.99 s	2.26 s

mult.*‐ Multiplicities‐ singlet (s), doublet (d).

**Table 2:** ^13^C NMR data of the components **2 b**, **3 b**, **4 b**, **5 b** of the mixture.

N ppm	≡C−I	=CI_2_	−IC=	−CH_2_‐C≡	−CH_2_−C≡	4‐C, Pz
2b	4.35	–	–	42.93	–	105.90
3b	–	24.30	113.09	–	66.46	106.21
4b	5.51	–	–	43.49	–	60.13
5b	–	25.63	111.84	–	66.99	60.68

Analyzing the qualitative composition of the mixture we took the ^1^H, ^13^C NMR data (Table 1, Table 2) of the pure isolated substance **2 b** as a basis. The substance **4 b** was similized to **4 e**, because in the case of both substances the C‐4 position of the pyrazole ring is iodinated. Substances **3 b** and **5 b** are analogues of substance **3 a** due to the presence of the 2,3,3‐triiodoallyl fragment.

Previous works proved^[16b]^ that the introduction of iodine into organic molecules significantly affected the chemical shifts of sp and sp^2^‐carbons (Table 2). Thus, the effect of iodine was most obvious on the sp‐carbon atoms of substances **2 b**, **4 b**. Therefore, the chemical shifts of the iodine‐substituted sp‐carbon appeared in the strong field. In the case of triiodo‐substituted **3 b**, **5 b** substances the chemical shifts of sp^2^‐carbon atoms differed significantly depending on the number of iodine atoms attached to these carbons. The characteristic chemical shifts of the carbon atoms of the methylene group contained in all the components of the mixture also depended on the nature of the carbon atom of the adjacent group. In the case of substances **4 b**, **5 b** the chemical shifts of iodinated 4‐C of the pyrazole ring appeared in the strong field comparing to the analogs **2 b**, **3 b** without iodine in the pyrazole ring.

Similar results were also reported in the iodination of isomeric derivative 5‐methyl‐1‐(prop‐2‐yn‐1‐yl)‐1*H*‐pyrazole (**1 c**). The results recorded by us show that the introduction of methyl groups significantly affects the regioselectivity of the iodination reaction, which can be explained by the increase in the nucleophilicity of the pyrazole ring due to the electron‐donating effect of the methyl group. The presented interpretation coincides with the analysis available in the literature.^[15]^

**Figure 4:**
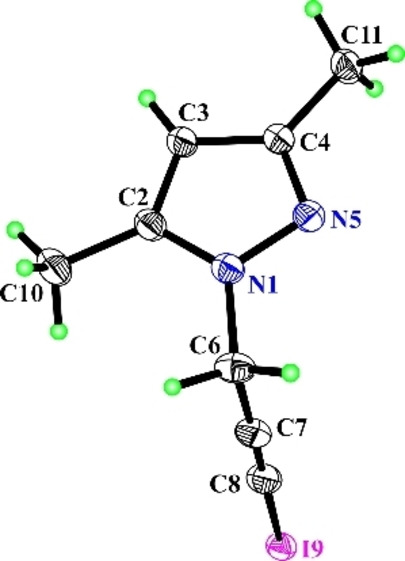
The molecular structure of compound **2 e** ^[17]^ with thermal displacement ellipsoids drawn at the 50 % probability level.

**Scheme 7:**
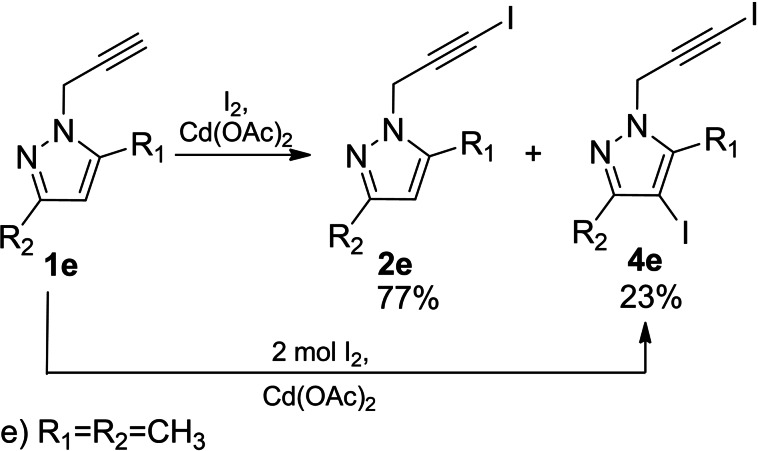
Iodination of propynylpyrazole derivative **1 e**.

Studying the chemical behavior of the pyrazole ring in the presence of two electron‐donating methyl groups was of particular interest. Therefore, 3,5‐dimethyl‐1‐(prop‐2‐yn‐1‐yl)‐1*H*‐pyrazole (**1 e**) was chosen as a model nucleophilic substrate. The regioselectivity of the iodination reaction mediated by cadmium (II) acetate was explored, depending on the molar ratio of reactants, as well as the nature of the solvent. It was found that in the polar aprotic solvent DMSO at the molar ratio of substrate/iodine/cadmium (II) acetate 1/1/1 a mixture consisting of two substances 1‐(3‐iodoprop‐2‐yn‐1‐yl)‐3,5‐dimethyl‐1*H*‐pyrazole (**2 e**) (Figure 4) and 4‐iodo‐1‐(3‐iodoprop‐2‐yn‐1‐yl)‐3,5‐dimethyl‐1*H*‐pyrazole (**4 e**) was formed. According to the ^1^H NMR data the molar ratio of the substances in the mixture is ∼ 77 %/23 % correspondingly (Scheme 7).

Consequently, the corresponding chemical shifts of methylene (N−CH_2_) protons 4.92 ppm, 4.98 ppm appearing in the form of singlets served as the basis for the identification of the composition of the mixture. 1‐(3‐iodoprop‐2‐yn‐1‐yl)‐3,5‐dimethyl‐1*H*‐pyrazole^[17]^ *(* **2 e**) was separated from mixture (**2 e** and **4 e**) by recrystallization with ethanol/H_2_O (1 : 1 vol. ratio) system. The molecular structure of compound **2 e** is shown in Figure 4.

CCDC 2383967 (for **2 e**) https://www.ccdc.cam.ac.uk/services/structures?id=doi:10.1002/open.202400443 contains the supplementary crystallographic data for this paper. These data are provided free of charge by the joint Cambridge Crystallographic Data Centre and Fachinformationszentrum Karlsruhe, Access Structures service, http://www.ccdc.cam.ac.uk/structures.

Summarizing the obtained results, we conclude that increasing the number of methyl groups contributes to rising the nucleophilicity of the pyrazole ring. The evidence of the latter is the absence of the iodination product at the C‐4 position of the pyrazole ring by implementing the iodination of one methyl group containing pyrazole derivatives **1 b**, **1 c**, **1 d** with 1/1/1 molar ratio of substrate/iodine/cadmium (II) acetate. Under similar conditions, the iodination of 3,5‐dimethyl‐1‐(prop‐2‐yn‐1‐yl)‐1*H*‐pyrazole (**1 e**) with two methyl groups gave a mixture consisting of both triple bond and aromatic ring hydrogen substitution products (∼77/23). As expected, the iodination of **1 e** with a twofold excess of iodine yielded only 4‐iodo‐1‐(3‐iodoprop‐2‐yn‐1‐yl)‐3,5‐dimethyl‐1*H*‐pyrazole (**4 e**). Tripling or quadrupling the amount of iodine did not significantly affect the regioselectivity of the reaction and iodination led only **4 e**.

Thus, during the iodination of **1 a** alone the increase in the amount of iodine did not lead to substitution at the C4 position of the pyrazole ring. However, the introduction of methyl groups into the ring contributed to the competitive reactions caused by the triple bond and the pyrazole ring.

**Figure 5:**
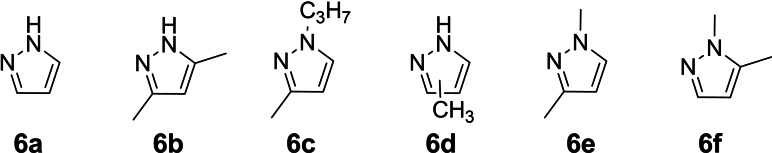
Pyrazole derivatives with electron‐donating groups.

In order to expand the range of tested substrates and exclude the influence of the propargylic group on the pyrazole ring the model compounds 1*H*‐pyrazole (**6 a)**, 3(*5*)‐methyl‐1*H*‐pyrazole (**6 d)**, 3,5‐dimethyl‐1*H*‐pyrazole (**6 b)**, 3‐methyl‐1‐propyl‐1*H*‐pyrazole (**6 c)**, 1,3‐dimethyl‐1*H*‐pyrazole (**6 e)**, 1,5‐dimethyl‐1*H*‐pyrazole (**6 f)** were chosen to promote the reduction in the number of directions of competitive electrophilic attacks (Figure 5).

**Scheme 8:**
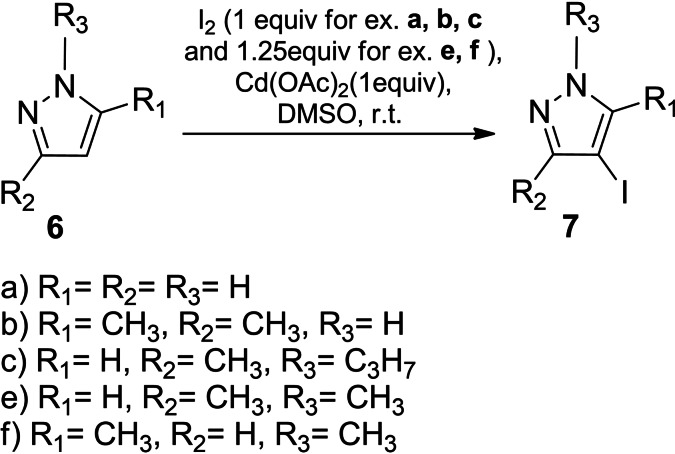
Iodination of pyrazole derivatives **6 a∼c**, **6 e**, **6 f**.

**Scheme 9:**
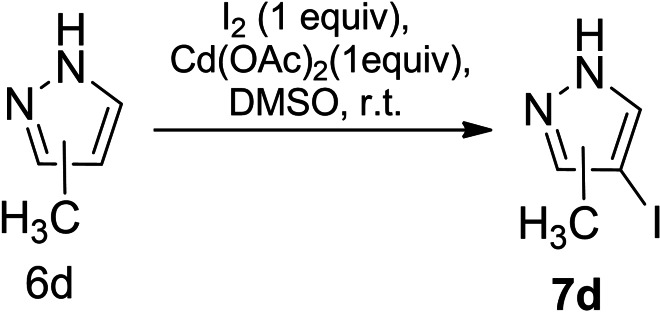
Iodination of pyrazole derivative **6 d**.

By iodinating **6 a**∼**d** with a molar ratio of substrate/ iodine/cadmium acetate 1 : 1 : 1 (1 : 1.25 : 1 in the case of **6 e**, **6 f**) the corresponding iodinated derivatives of the C‐4 position of the pyrazole ring 4‐iodo‐1*H*‐pyrazole (**7 a**), 4‐iodo‐3,5‐dimethyl‐1*H*‐pyrazole (**7 b**), 4‐iodo‐3‐methyl‐1‐propyl‐1*H*‐pyrazole (**7 c**), 4‐iodo‐3(*5*)‐methyl‐1*H*‐pyrazole (**7 d**), 4‐iodo‐1,3‐dimethyl‐1*H*‐pyrazole (**7 e**), 4‐iodo‐1,5‐dimethyl‐1*H*‐pyrazole (**7 f**) were obtained (Scheme 8, 9).

The increase in the yields of the obtained iodopyrazole derivatives **7 a∼f** coincided with the rise in the nucleophilicity of the pyrazole ring due to the electron‐donating effect of the alkyl groups. When mentioned substrates **6 a∼d** were treated with a twofold excess of iodine the regiochemistry of the reaction was not changed; only the most nucleophilic C‐4 position of the pyrazole ring was iodinated.

**Figure 6:**
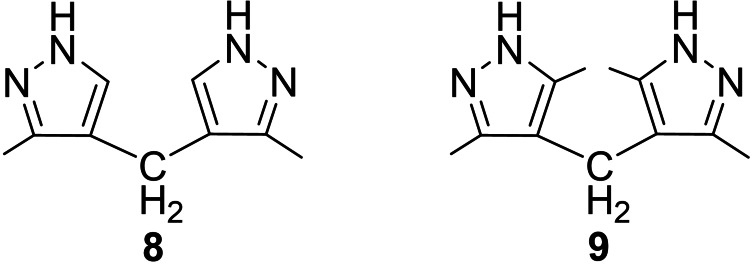
Pyrazole derivatives with electron‐donating groups dimerized by C‐4 positions

The study of iodination of pyrazole systems **8**, **9** (Figure 6). with electron‐donating groups dimerized by C‐4 positions^[18]^ was of great interest. It was found that iodination did not occur in the mentioned examples. The latter proved again that the electrophilic substitution in the pyrazole ring is more special to the C‐4 position.

**Scheme 10:**
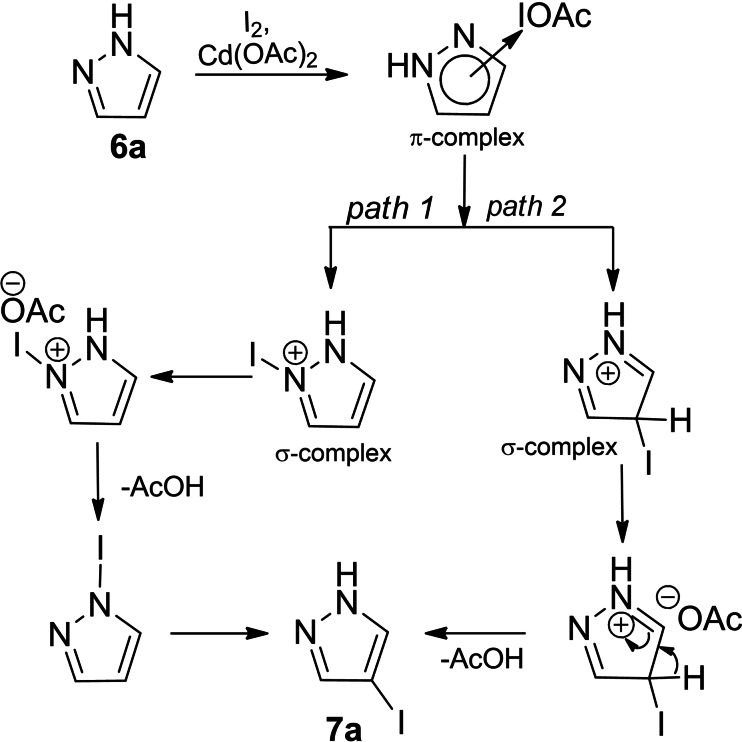
Plausible mechanism of iodination of pyrazole derivatives with the acetyl hypoiodite.

The iodination of the pyrazole ring initiated by cadmium(II) acetate could be represented by the following sequence of conversions. As mentioned above, the source of electrophilic iodine was acetyl hypoiodite obtained from the interaction of cadmium (II) acetate with molecular iodine. The π‐complex formed by the interaction of the acetyl hypoiodite and the π‐system of the pyrazole ring quickly transformed into a σ‐complex (*path 1*) where electrophilic iodine was coordinated to the most basic pyridine nitrogen atom of the pyrazole ring leading to the intermediate iodopyrazol‐2‐ium acetate. It was stabilized by elimination of acetic acid to form N‐iodopyrazole the rearrangement of which produced 4‐iodopyrazole (Scheme 10).

The transformation of the π‐complex formed by the interaction of the electrophilic iodine and the π‐system of the pyrazole ring into the σ‐complex (*path 2*) with the I−C‐4 bond the aromaticity restoration of which led to the target 4‐iodopyrazole was not excluded either. The efficiency of iodination of pyrazole derivatives largely depends on the nature of the substituents and their position in the pyrazole ring.

Comparing the data of iodination of unsubstituted to N, C‐substituted pyrazole derivatives with electron donating groups, it was found that methyl, propyl groups significantly affect the duration of the reaction, as well as the yields of separated target substances (Table 3).

**Table 3:** Comparing the experimental data of iodination of pyrazole derivatives **6 a∼f**.

N	Iodo‐1*H*‐pyrazole derivatives	duration of the reaction (h)	Yeild (%)
7a	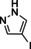	4	65
7b	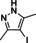	2	81
7c	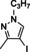	2.5	88
7d	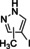	3	70
7e	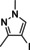	2	72
7f	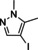	3	68

However, the acceptor propargyl group significantly reduces the nucleophilicity of the pyrazole ring, which was proved by experimental data with the absence of C‐4‐iodination products. If both electron donating alkyl and electron withdrawing propargyl groups are present in the pyrazole ring, the number of iodination directions increases and the chemoselectivity accordingly decreases.

## Conclusions

The presented work was aimed at studying the chemical behavior of both N,C substituted and unsubstituted pyrazole derivatives in the iodination reactions initiated by cadmium (II) acetate and by optimizing the role of the solvent and the molar ratios of the reactants.As a result of research, it was found that the C‐4 position of the pyrazole ring was more sensitive to the attack of electrophilic iodine in the process iodination reactions mediated by cadmium (II) acetate. The reactivity of the latter significantly increased due to the influence of electron donating alkyl groups, and decreased in the case of N‐propargyl substituent. The example of N‐propargyl pyrazole showed that the substitution of the terminal triple bond hydrogen atom by iodine, the subsequent electrophilic iodination of the obtained iodoalkynes, as well as the possibility of direct produce of triiodosubstituted pyrazole derivatives from N‐propargyl pyrazole in a one‐pot version, were substantiated. The introduction of methyl groups into the N‐propargyl pyrazole ring also contributed to the substitution reactions at the C4 position of the aromatic ring and to affording the immediate iodinated products of the pyrazole ring. In the case of N, C‐alkyl substituted and unsubstituted pyrazoles, only the C‐4 position of the ring was the target of the iodination reaction initiated by cadmium (II) acetate.

## Experimental Section

^1^H and ^13^C NMR spectra have been recorded on a

– Varian Mercury‐300 VX spectrometer (Varian Inc., Palo Alto, CA, USA) with resonance frequencies of 300.086 and 75.465 MHz, respectively
– Avance 400 Neo (Bruker BioSpin, Germany) with resonance frequencies of 400.15 and 100.618 MHz, respectively. Chemical shifts are given for CDCl_3_, DMSO‐d6/CCl_4_ solution: 1/3 relative to internal TMS at 303 K.

Signal assignments are made based on DEPT and HMQC spectra. Chemical shifts (δ) are in parts per million (ppm) internally referenced to the solvent nuclei. Multiplicities are assigned as singlet (s), broad singlet (br), doublet (d), triplet (t), quartet (q), multiplet (m) or a combination of these (e. g., dd, dq, dt) and coupling constants (J) are reported in Hertz (Hz). The reaction progress was monitored by TLC on ′′Silica gel 60 F_254_′′ plates (Th Geyer, Germany), developers ‐ KMnO_4_ and iodine vapor. Melting points were measured on a Stuart SMP11 apparatus (designed in UK, assembled in PRC) in open capillary tubes. FT‐IR spectra were recorded using KBr disks and vaseline oil on a Nicolet Avatar 330 FT‐IR spectrometer (Thermo Nicolet, Foster, CA, USA). Mass spectra were obtained on a XEVO G3 QTof spectrometer (Waters Corporation Company, Milford, MA, USA). Reaction products were purified by column chromatography on silica gel or by distillation under reduced pressure. All commercially available reagents and solvents were purchased from standard suppliers Sigma Aldrich (Saint Louis, MO, USA) and used without further purification.

### General Procedure A

#### Iodination of Propynylpyrazole Derivatives 1 a∼e and 2 a

Cadmium (II) acetate (460 mg, 2 mmol) was dissolved in 5 ml DMSO at 25 °C and propynylpyrazoles compounds **1 a**, **b**, **c**, **d**, **e** and **2 a** (2 mmol) were added dropwise. The reaction mixture was stirred for 30 min then grated crystalline iodine (508 mg, 2 mmol) was added and the stirring was continued for 4 h. The reaction progress was monitored by TLC. Upon completion the reaction mixture was filtered, the filtrate was quenched with a Na_2_S_2_O_3_ solution (10 %, 5 ml), washed with a Na_2_CO_3_ solution (20 %, 5 ml), extracted with dichloromethane and dried over anhydrous MgSO_4_. After filtration the solvent was removed under reduced pressure to afford the crude product.

### General Procedure B

#### Iodination of Pyrazole Derivatives 6 a∼d

Cadmium (II) acetate (230 mg, 1 mmol) was dissolved in 5 ml DMSO at 25 °C and pyrazoles compounds **6 a*∼* d** (1 mmol) were added dropwise. The reaction mixture was stirred for 30 min then grated crystalline iodine (254 mg, 1 mmol) was added and stirring was continued for 1 h. The reaction progress was monitored by TLC. Upon completion the reaction (∼4 h) mixture was filtered, the filtrate was quenched with a Na_2_S_2_O_3_ solution (10 %, 4 ml), washed with a Na_2_CO_3_ solution (20 %, 4 ml), extracted with dichloromethane and dried over anhydrous MgSO_4_. After filtration the solvent was removed under reduced pressure to afford the crude product.

### General Procedure C

#### Iodination of N‐substituted Pyrazole Derivatives 6 e, 6 f

Cadmium (II) acetate (230 mg, 1 mmol) was dissolved in 5 ml DMSO at 25 °C and N‐substituted pyrazole derivatives **6 e**, **f** (1 mmol) were added dropwise. The reaction mixture was stirred for 30 min then grated crystalline iodine (318 mg, 1.25 mmol) was added and stirring was continued for 2 h. The reaction progress was monitored by TLC. Upon completion the reaction (∼3 h) mixture was filtered, the filtrate was quenched with a Na_2_S_2_O_3_ solution (10 %, 4 ml), washed with a Na_2_CO_3_ solution (20 %, 4 ml), extracted with dichloromethane and dried over anhydrous MgSO_4_. After filtration the solvent was removed under reduced pressure to afford the crude product.

### General Procedure D

#### Iodination of Pyrazole Derivatives 1 a, 1 b, 1e

Cadmium (II) acetate (230 mg, 1 mmol) was dissolved in 5 ml DMSO at 25 °C and of pyrazole derivatives **1 a**, **1 b**, **1 e** (1 mmol) were added dropwise. The reaction mixture was stirred for 30 min then grated crystalline iodine (508 mg, 2 mmol) was added and stirring was continued for 3 h. The reaction progress was monitored by TLC. Upon completion the reaction mixture was filtered, the filtrate was quenched with a Na_2_S_2_O_3_ solution (10 %, 6 ml), washed with a Na_2_CO_3_ solution (20 %, 6 ml), extracted with dichloromethane and dried over anhydrous MgSO_4_. After filtration the solvent was removed under reduced pressure to afford the crude product.

## Supporting Information

The authors have cited additional references within the Supporting Information (Ref. [19–23]).

## Conflict of Interests

The authors declare no conflict of interest.

## Supporting information

As a service to our authors and readers, this journal provides supporting information supplied by the authors. Such materials are peer reviewed and may be re‐organized for online delivery, but are not copy‐edited or typeset. Technical support issues arising from supporting information (other than missing files) should be addressed to the authors.

Supporting Information

## Data Availability

The data that support the findings of this study are available in the supplementary material of this article.
