# Supplementary material for: Efficient Regioselective Iodination of Pyrazole Derivatives Mediated by Cadmium(II) Acetate
Source: ChemistryOpen. 2025 Feb 28;14(7):e202400443. doi: 10.1002/open.202400443 (PMC13042630; doi:10.1002/open.202400443)
Supplement: Supplementary file 1 — Supporting Information [file OPEN-14-e202400443-s001.pdf]

ChemistryOpen

Supporting Information

Efficient Regioselective Iodination of Pyrazole Derivatives Mediated by Cadmium(II) Acetate

Nina G. Hobosyan, Kristine V. Balyan,* Lusine A. Movsisyan,* Varduhi S. Hovsepyan, Armen G. Ayvazyan, Henrik A. Panosyan, Hovhannes S. Attaryan, Hmayak B. Sargsyan, and Haykanush R. Pogosyan

Supporting Information

Efficient Regioselective Iodination of Pyrazole Derivatives Mediated by Cadmium(II) Acetate

Nina G. Hobosyan, Kristine V. Balyan*, Lusine A. Movsisyan*, Varduhi S. Hovsepyan, Armen G. Ayvazyan, Henrik A. Panosyan, Hovhannes S.Attaryan, Hmayak B. Sargsyan and Haykanush R. Pogosyan

E-mail: balyankristine1979@gmail.com
E-mail: lusmov372@gmail.com

Table of Contents

1. General Procedure A S2
2. General Procedure B S5
3. General Procedure C S7
4. General Procedure D S9
5. NMR spectra S12
6. HRMS Spectra S41
7. IR Data S61
8. X-ray Crystallographic Data S71

Experimental Section

1. General Procedure A

Iodination of propynylpyrazole derivatives 1a~ e and 2a

Cadmium (II) acetate (460 mg, 2mmol) was dissolved in 5 ml DMSO at 25 °C and propynylpyrazoles compounds **1a, b, c, d, e** and **2a** (2 mmol) were added dropwise. The reaction mixture was stirred for 30 min then grated crystalline iodine (508 mg, 2 mmol) was added and the stirring was continued for 4 h. The reaction progress was monitored by TLC. Upon completion the reaction mixture was filtered, the filtrate was quenched with a $Na_2S_2O_3$ solution (10 %, 5 ml), washed with a Na_2CO_3 solution (20 %, 5 ml), extracted with dichloromethane and dried over anhydrous $MgSO_4$. After filtration the solvent was removed under reduced pressure to afford the crude product.

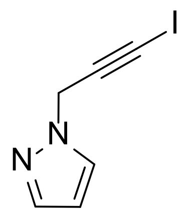

1-(3-iodoprop-2-yn-1-yl)-1*H*-pyrazole (2a). According to the *general procedure A* 1-(prop-2-yn-1-yl)-1*H*-pyrazole (**1a**) (212 mg, 2.0 mmol) afforded 1-(3-iodoprop-2-yn-1-yl)-1*H*-pyrazole (**2a**) which was purified by recrystallization from ethanol to get 325 mg (70 % yield) in light brown solid. R_f=0.6 (C_6H_{14}/$(CH_3)_2CO$ 1:1); m.p. 82 °C; ^1H NMR (300.086 MHz, $CDCl_3$): δ=7.57 (d, J = 2.3 Hz, 1H; H-5, Pz), 7.53 (d, J = 1.8, Hz, 1H; H-3, Pz), 6.30 (dd, J = 2.3, 1.8 Hz, 1H; H-4, Pz), 5.10 (s, 2H; CH_2); ^{13}C NMR (75.464 MHz, $CDCl_3$): δ=140.1 (5-C; Pz), 128.9 (3-C; Pz), 106.3 (4-C; Pz), 87.1 (CCH_2), 43.3 (CH_2), 4.0 (CI); HRMS (TOF MS AP+): *m/z* for $C_6H_5IN_2$ $[M+H]^+$ calcd: 232.9576; found: 232.9621; IR$_{\tilde{v}}$ max (cm^{-1}): 2184(C≡C), 1515 (Pz-ring).

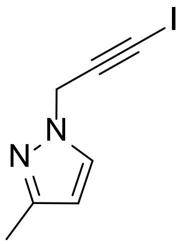

1-(3-iodoprop-2-yn-1-yl)-3-methyl-1*H*-pyrazole (**2b**). According to the *general procedure A* 3-methyl-1-(prop-2-yn-1-yl)-1*H*-pyrazole (**1b**) (240 mg, 2.0 mmol) afforded 1-(3-iodoprop-2-yn-1-yl)-3-methyl-1*H*-pyrazole (**2b**) which was purified by recrystallization from ethanol to get 403 mg (82 % yield) in milky white solid. R_f=0.64 ($C_6H_{14}/(C_2H_5)_2O$ 1:1); m.p.102-105 °C; ^{1}H NMR (300.086 MHz, $CDCl_3$) δ=7.44 (d, J = 2.3 Hz, 1H; H-5, Pz), 6.05 (dq, J = 2.3, 0.5 Hz, 1H; H-4, Pz), 5.00 (s, 2H; CH_2), 2.27 (d, J = 0.5, 3H; CH_3); ^{13}C NMR (75.464 MHz, $CDCl_3$) δ=149.4 (3-C; Pz), 129.6 (5-C; Pz), 105.9 (4-C; Pz), 87.3 (CCH_2), 43.0 (CH_2), 13.6 (CH_3), 3.9 (CI); HRMS (TOF MS ES+) m/z for $C_7H_7IN_2$ $[M+H]^+$ calcd: 246.9732, found: 246.9483; IR$_{\tilde{\nu}}$ max (cm^{-1}): 2193(C≡C), 1516 (Pz-ring).

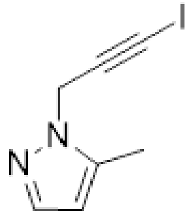

1-(3-iodoprop-2-yn-1-yl)-5-methyl-1*H*-pyrazole (**2c**). According to the *general procedure A* 5-methyl-1-(prop-2-yn-1-yl)-1*H*-pyrazole (**1c**) (240 mg, 2.0 mmol) afforded 1-(3-iodoprop-2-yn-1-yl)-5-methyl-1*H*-pyrazole (**2c**) which was purified by recrystallization from ethanol to get 271 mg (55 % yield) in milky white solid. R_f=0.61 ($C_6H_{14}/(C_2H_5)_2O$ 1:1); m.p.84-87 °C; ^{1}H NMR (300.086 MHz, $CDCl_3$) δ=7.39 (d, J = 1.8 Hz, 1H; H-3, Pz), 6.04 (dq, J = 1.8, 0.8 Hz, 1H; H-4, Pz), 5.00 (br, 2H; CH_2), 2.35 (br, 3H; CH_3); ^{13}C NMR (75.464 MHz, $CDCl_3$) δ=139.0 (3-C; Pz), 138.4 (5-C; Pz), 106.2 (4-C; Pz), 87.6 (CCH_2), 40.9 (CH_2), 11.1 (CH_3), 2.8 (CI); HRMS (TOF MS ES+) for $C_7H_7IN_2$ $[M+H]^+$ calcd: 246.9732, found: 246.9742; IR$_{\tilde{\nu}}$ max (cm^{-1}): 2184 (C≡C), 1543 (Pz-ring).

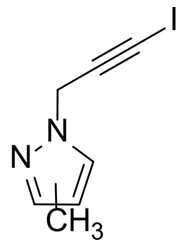

1-(3-iodoprop-2-yn-1-yl)-3(*5*)-methyl-1*H*-pyrazoles (**2d**). According to the *general procedure A* mixture of 3(*5*)-methyl-1-(prop-2-yn-1-yl)-1*H*-pyrazoles (**1d**) (60% : 40%) (240 mg, 2.0 mmol) afforded mixture of 1-(3-iodoprop-2-yn-1-yl)-3(*5*)-methyl-1*H*-pyrazoles (**2d**) (~60% : ~40%) (330 mg, 67 % yield) in white solid.

- *1-(3-iodoprop-2-yn-1-yl)-3-methyl-1H-pyrazole (**2b**)*. ^{1}H NMR (400.15 MHz, $CDCl_3$) *δ*=7.45 (d, *J* = 2.2 Hz, 1H; H-5, Pz), 6.07 (d, *J* = 2.2 Hz, 1H; H-4, Pz), 5.02 (s, 2H; CH_2), 2.28 (s, 3H; CH_3); ^{13}C NMR (100.618 MHz, $CDCl_3$) *δ*=149.4 (3-C; Pz), 129.7 (5-C; Pz), 105.9 (4-C; Pz), 87.3 (CCH_2), 43.0 (CH_2), 13.6 (CH_3), 3.7 (CI).
- *1-(3-iodoprop-2-yn-1-yl)-5-methyl-1H-pyrazole (**2c**).* ^{1}H NMR (400.15 MHz, $CDCl_3$) *δ*=7.40 (d, *J* = 1.8 Hz, 1H; H-3, Pz), 6.05 (dq, *J* = 1.8, 0.8 Hz, 1H; H-4, Pz), 5.00 (s, 2H; CH_2), 2.35 (d, *J* = 0.8 Hz, 3H; CH_3); ^{13}C NMR (100.618 MHz, $CDCl_3$) *δ*=139.0 (3-C; Pz), 138.4 (5-C; Pz), 106.2 (4-C; Pz), 87.6 (CCH_2), 40.8 (CH_2), 11.1 (CH_3), 2.8 (CI).

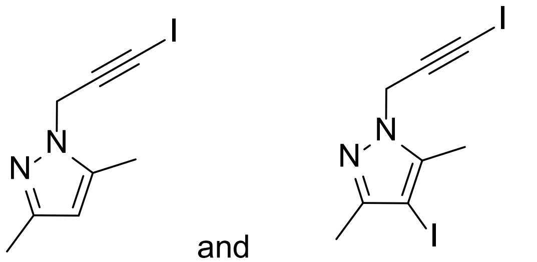

1-(3-iodoprop-2-yn-1-yl)-3,5-dimethyl-1*H*-pyrazole (**2e**) and **4-iodo-1-(3-iodoprop-2-yn-1-yl)-3,5-dimethyl-1*H*-pyrazole (4e)**. According to the *general procedure A* 3,5-dimethyl-1-(prop-2-yn-1-yl)-1*H*-pyrazole (**1e**) (268 mg, 2.0 mmol) afforded mixture of 1-(3-iodoprop-2-yn-1-yl)-3,5-dimethyl-1*H*-pyrazole and 4-iodo-1-(3-iodoprop-2-yn-1-yl)-3,5-dimethyl-1*H*-pyrazole (**2e, 4e**) (150 mg) in light brown solid.

- *1-(3-iodoprop-2-yn-1-yl)-3,5-dimethyl-1H-pyrazole* (***2e***). ^{1}H NMR (300.086 MHz, $CDCl_3$) δ=5.83 (br, 1H; H-4, Pz), 4.92 (s, 2H; CH_2), 2.30 (d, J = 0.8 Hz, 3H; 5-CH_3), 2.22 (s, 3H, 3-CH_3).
- *4-iodo-1-(3-iodoprop-2-yn-1-yl)-3,5-dimethyl-1H-pyrazole* (***4e***). ^{1}H NMR (300.086 MHz, $CDCl_3$) δ=4.98 (s, 2H; CH_2), 2.34 (s, 3H; 5-CH_3), 2.22 (s, 3H; 3-CH_3).

1-(3-iodoprop-2-yn-1-yl)-3,5-dimethyl-1*H*-pyrazole (**2e**) was separated from above mentioned mixture (**2e** and **4e**) by recrystallization with ethanol / H_2O (1:1 vol. ratio) system to get 100 mg white solid. R_f=0.69 (C_6H_{14}/$(C_2H_5)_2O$ 1:1); m.p.120-122 °C; ^{1}H NMR (300.086 MHz, $CDCl_3$) δ=5.83 (br, 1H; H-4, Pz), 4.93 (s, 2H; CH_2), 2.29 (d, J = 0.6 Hz, 3H; 5-CH_3), 2.22 (s, 3H; 3-CH_3); ^{13}C NMR (75.464 MHz, $CDCl_3$) δ=148.1 (5-C; Pz), 139.4 (3-C; Pz), 106.1 (4-C; Pz), 87.8 (CCH_2), 40.4 (CH_2), 13.5 (CH_3), 11.1 (CH_3), 2.6 (CI).

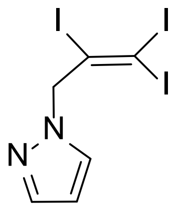

1-(2,3,3-triiodoallyl)-1*H*-pyrazole (**3a**). According to the *general procedure A* 1-(3-iodoprop-2-yn-1-yl)-1*H*-pyrazole (**2a**) (464 mg, 2 mmol) afforded 1-(2,3,3-triiodoallyl)-1*H*-pyrazole (**3a**) which was purified by recrystallization from ethanol to get 729 mg (75 % yield) in light brown solid. R_f=0.75 (C_6H_{14}/($(CH_3)_2CO$, 1:1); m.p.129 °C; ^{1}H NMR (300.086 MHz, $CDCl_3$) δ=7.55 (dd, J = 1.9, J = 0.6 Hz, 1H; H-3, Pz), 7.45 (dd, J = 2.3, 0.6 Hz, 1H; H-5, Pz), 6.29 (dd, J = 2.3,1.9 Hz, 1H; H-4, Pz), 5.09 (s, 2H; CH_2); ^{13}C NMR (75.464 MHz, $CDCl_3$) δ=140.3 (5-C; Pz), 129.7 (3-C; Pz), 112.5 (=CI), 106.4 (4-C; Pz), 66.7 (CH_2), 24.4 (=CI_2); HRMS (TOF MS ES+) m/z for $C_6H_5I_3N_2$ $[M+H]^+$ calcd: 486.7665. found: 487.7255; $IR_{\tilde{\nu}}$ max (cm^{-1}): 1515 Pz-(ring).

2. General Procedure B

Iodination of pyrazole derivatives 6 a~d

Cadmium (II) acetate (230 mg, 1 mmol) was dissolved in 5 ml DMSO at 25 °C and pyrazoles compounds **6a~ d** (1 mmol) were added dropwise. The reaction mixture was stirred for 30 min then grated crystalline iodine (254 mg, 1 mmol) was added and stirring

was continued for 1 h. The reaction progress was monitored by TLC. Upon completion the reaction (~4 h.) mixture was filtered, the filtrate was quenched with a $Na_2S_2O_3$ solution (10 %, 4 ml), washed with a Na_2CO_3 solution (20 %, 4 ml), extracted with dichloromethane and dried over anhydrous $MgSO_4$. After filtration the solvent was removed under reduced pressure to afford the crude product.

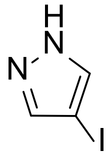

4-iodo-1*H*-pyrazole (**7a**). According to the *general procedure B* 1*H*-pyrazole (**6a**) (68 mg, 1.0 mmol) afforded 4-iodo-1*H*-pyrazole (**7a)** which was purified by recrystallization from ethanol to get 126 mg (65 % yield) in yellowish solid. R_f=0.69 (C_6H_{14}/$(C_2H_5)_2O$ 1:1); m.p. 91-93 °C; ^1H NMR (300.086 MHz, $CDCl_3$) δ=11.34 (br, 1H; NH), 7.66 (s, 2H; H-3,5, Pz); ^{13}C NMR (75.464 MHz, $CDCl_3$) δ=139.1 (3,5-C; Pz), 57.00 (4-C; Pz); HRMS (TOF MS ES+) m/z for $C_3H_3IN_2$ $[M+H]^+$ calcd: 194.9419, found: 194.9411; $IR_{\tilde{v}}$ max (cm^{-1}): 1537 (Pz-ring).

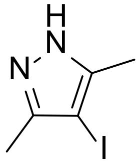

4-iodo-3,5-dimethyl-1*H*-pyrazole[19,20] (7b). According to the *general procedure B* 3,5-dimethyl-1*H*-pyrazole (**6b**) (96 mg, 1.0 mmol) afforded 4-iodo-3,5-dimethyl-1*H*-pyrazole (**7b**) which was purified by recrystallization from ethanol to get 180 mg (81 % yield) in yellowish solid. R_f=0.65 (C_6H_{14}/$(C_2H_5)_2O$ 1:1)); m.p.118-120 °C; ^1H NMR (300.086 MHz, $CDCl_3$) δ=10.84 (br, 1H; NH), 2.28 (s, 6H; 3,5-CH_3); ^{13}C NMR (75.464 MHz, $CDCl_3$) δ= 146.8 (3,5-C; Pz), 63.3 (4-C; Pz), 13.1 (3,5-CH_3); HRMS (TOF MS ES+) m/z for $C_5H_7IN_2$ $[M+H]^+$ calcd: 222.9732, found: 222.9734; $IR_{\tilde{v}}$ max (cm^{-1}): 1555 (Pz-ring).

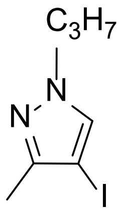

4-iodo-3-methyl-1-propyl-1*H*-pyrazole (7c). According to the *general procedure B* 3-methyl-1-propyl-1*H*-pyrazole (**6c**) (124 mg, 1.0 mmol) afforded 4-iodo-3-methyl-1-propyl-1*H*-pyrazole (**7c**) 220 mg (88 % yield) in yellowish oil. R_f=0.70 ($C_6H_{14}/(C_2H_5)_2O$ 1:1); b.p. 84 °C (4 mm Hg); ^{1}H NMR (300.086 MHz, $CDCl_3$) δ=7.33 (s, 1H; H-5, Pz), 4.00 (t, *J* = 7.1, 2H; NCH_2), 2.25 (s, 3H; 3-CH_3), 1.91-1.79 (m, 2H; CH_2, Pr), 0.91 (t, *J* = 7.4, 3H; CH_3, Pr); ^{13}C NMR (75.464 MHz, $CDCl_3$) δ=150.6 (3-C; Pz), 134.1 (5-C; Pz), 58.6 (4-C; Pz), 54.2 (NCH_2), 23.8 (CH_2), 13.6 (CH_3; Pr), 11.2 (3-CH_3); HRMS (TOF MS AP+) m/z for $C_7H_{11}IN_2$ $[M+H]^+$ calcd: 251.0045, found: 251.0029; IR$_{\tilde{\nu}}$ max (cm^{-1}): 1513 (Pz-ring).

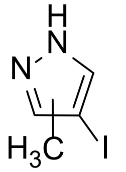

4-iodo-3(*5*)-methyl-1*H*-pyrazole (7d). According to the *general procedure B* 3(*5*)-methyl-1*H*-pyrazole (**6d**) (82 mg, 1.0 mmol) afforded 4-iodo-3(*5*)-methyl-1*H*-pyrazole (**6d**) which was purified by recrystallization from ethanol to get 146 mg (70 % yield) in yellowish solid. R_f=0.75 ($C_6H_{14}/(C_2H_5)_2O$ 1:1); m.p. 90-92 °C; ^{1}H NMR (300.086 MHz, $CDCl_3$) δ=10.94 (br, 1H; NH), 7.55 (s, 1H; CH, Pz), 2.27 (s, 3H; 3(*5*)-CH_3); ^{13}C NMR (75.464 MHz, $CDCl_3$) δ=144.9 (C; Pz), 140.9 (C; Pz), 59.8 (4-C; Pz), 12.1 (CH_3); HRMS (TOF MS ES+) m/z for $C_4H_5IN_2$ $[M+H]^+$ calcd: 208.9576, found: 208.9556; IR$_{\tilde{\nu}}$ max (cm^{-1}): 1551 (Pz-ring).

3. General Procedure C

Iodination of N-substituted pyrazole derivatives 6e, 6f

Cadmium (II) acetate (230 mg, 1 mmol) was dissolved in 5 ml DMSO at 25 °C and N-substituted pyrazole derivatives **6 e, f** (1 mmol) were added dropwise. The reaction mixture was stirred for 30 min then grated crystalline iodine (318 mg, 1.25 mmol) was added and stirring was continued for 2 h. The reaction progress was monitored by TLC.

Upon completion the reaction (~3 h.) mixture was filtered, the filtrate was quenched with a $Na_2S_2O_3$ solution (10%, 4 ml), washed with a Na_2CO_3 solution (20%, 4 ml), extracted with dichloromethane and dried over anhydrous $MgSO_4$. After filtration the solvent was removed under reduced pressure to afford the crude product.

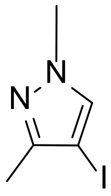

4-iodo-1,3-dimethyl-1*H*-pyrazole[21] **(7e).** According to the general *procedure C* 1,3-dimethyl-1*H*-pyrazole (**6e**) (96 mg, 1.0 mmol) afforded 4-iodo-1,3-dimethyl-1*H*-pyrazole (**7e**) which was purified by recrystallization from ethanol to get 151 mg (68 % yield) in yellowish oil. R_f=0.79 (C_6H_{14}/$(C_2H_5)_2O$ 1:1); m.p. 98-100 °C; ^{1}H NMR (300.086 MHz, $CDCl_3$) δ=7.30 (s, 1H; H-5, Pz), 3.83 (s, 3H; NCH_3), 2.23 (s, 3H; 3-CH_3); ^{13}C NMR (75.464 MHz, $CDCl_3$) δ=150.9 (3-C; Pz), 135.0 (5-C; Pz), 58.9 (4-C; Pz), 39.1 (NCH_3), 13.5 (3-CH_3).

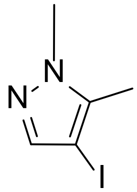

4-iodo-1,5-dimethyl-1*H*-pyrazole[20,21,22] **(7f).** According to the *general procedure C* 1,5-dimethyl-1*H*-pyrazole (**6f**) (96 mg, 1.0 mmol) afforded 4-iodo-1,3-dimethyl-1*H*-pyrazole (**7f**) 160 mg (72 % yield) in yellowish solid. R_f=0.73 (C_6H_{14}/$(C_2H_5)_2O$ 1:1); m.p. 99-101 °C; ^{1}H NMR (300.086 MHz, $CDCl_3$) δ=7.41 (s,1H; H-3, Pz), 3.85 (s, 3H; NCH_3), 2.29 (s, 5H; CH_3); ^{13}C NMR (75.464 MHz, $CDCl_3$) δ=142.8 (3-C; Pz), 140.1 (5-C; Pz), 58.9 (4-C; Pz), 37.5 (NCH_3), 11.4 (5-CH_3); HRMS (TOF MS ES+) m/z for $C_5H_7IN_2$ $[M+H]^+$ calcd: 222.9732, found: 222.9742; IR$_{\tilde{\nu}}$ max (cm^{-1}): 1524 (Pz-ring).

4. General Procedure D

Iodination of pyrazole derivatives 1a, 1b, 1e

Cadmium (II) acetate (230 mg, 1 mmol) was dissolved in 5 ml DMSO at 25 °C and of pyrazole derivatives **1a, 1b, 1e** (1 mmol) were added dropwise. The reaction mixture was stirred for 30 min then grated crystalline iodine (508 mg, 2 mmol) was added and stirring was continued for 3 h. The reaction progress was monitored by TLC. Upon completion the reaction mixture was filtered, the filtrate was quenched with a $Na_2S_2O_3$ solution (10 %, 6 ml), washed with a Na_2CO_3 solution (20 %, 6 ml), extracted with dichloromethane and dried over anhydrous $MgSO_4$. After filtration the solvent was removed under reduced pressure to afford the crude product.

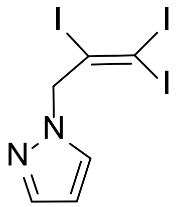

1-(2,3,3-triiodoallyl)-1*H*-pyrazole (3a). According to the *general procedure D* 1-(prop-2-yn-1-yl)-1*H*-pyrazole (**1a**) (106 mg, 1.0 mmol) afforded 1-(2,3,3-triiodoallyl)-1*H*-pyrazole (**3a**) which was purified by recrystallization from ethanol to get 267 mg (55% yield). Physical data of 1-(2,3,3-triiodoallyl)-1*H*-pyrazole (**3a**) are described in *procedure A.*

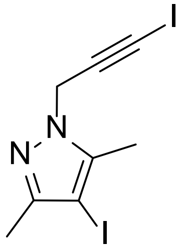

4-iodo-1-(3-iodoprop-2-yn-1-yl)-3,5-dimethyl-1*H*-pyrazole (4e). According to the general *procedure D* 3,5-dimethyl-1-(prop-2-yn-1-yl)-1*H*-pyrazole (**1e**) (134 mg, 1.0 mmol) afforded 4-iodo-1-(3-iodoprop-2-yn-1-yl)-3,5-dimethyl-1*H*-pyrazole (**4e**) which was purified by recrystallization from ethanol to get 332 mg (86% yield) in white solid. R_f=0.73 (C_6H_{14}/$(C_2H_5)_2O$ 1:1); m.p. 147-150 °C; ^{1}H NMR (300.088 MHz, [D6] DMSO/CCl_4 1/3) δ=4.96 (s, 2H; CH_2N), 2.32 (s, 3H; 5-CH_3), 2.12 (s, 3H; 3-CH_3); ^{13}C NMR (75.465 MHz,

[D6] DMSO/CCl_4 1/3) δ=147.9 (C; Pz), 139.6 (C; Pz), 86.0 (CCH_2), 62.8 (4-C; Pz), 41.0 (CH_2), 13.3 (CH_3), 11.2 (CH_3), 10.8 (CI); ^{1}H NMR (300.086 MHz, $CDCl_3$) δ=4.99 (s, 2H; CH_2N), 2.34 (s, 3H; 5-CH_3), 2.22 (s, 3H; 3-CH_3); ^{13}C NMR (75.465 MHz, $CDCl_3$) δ=150.1 (3-C; Pz), 140.8 (5-C; Pz), 87.4 (CCH_2), 63.9 (4-C; Pz), 41.8 (CH_2), 14.1 (3-C; CH_3), 12.1 (5-C; CH_3), 3.1 (CI); HRMS (TOF MS ES+) m/z for $C_8H_8I_2N_2$ $[M+H]^+$ calcd: 386.8855, found: 386.8839; IR$_{\tilde{v}}$ max (cm^{-1}): 2187(C≡C), 1530 (Pz-ring).

The assignment of signals in the ^{1}H and ^{13}C NMR spectra of compound **4e** was made on the basis of two-dimensional NMR experiments of NOESY, HSQC and HMBC. Thus, the presence of NOE between the 1-CH_2 protons and one of the methyl groups indicated that the 5-methyl group protons appeared in a weaker magnetic field. Subsequently, it was obvious that the ^{13}C NMR signal of the 5-Me group appeared in a stronger field than the signal of the 3-Me group based on the HSQC spectrum. After assignment of the methyl group signals with use of the HMBC spectrum the assignment of the carbon atoms of the pyrazole ring in positions 3 and 5 was carried out.

Mixture of 1-(3-iodoprop-2-yn-1-yl)-3-methyl-1*H*-pyrazole (**2b)**, 3-methyl-1-(2,3,3-triiodoallyl)-1*H*-pyrazole (**3b)**, 4-iodo-1-(3-iodoprop-2-yn-1-yl)-3-methyl-1*H*-pyrazole (**4b).** According to the general *procedure D* 3-methyl-1-(prop-2-yn-1-yl)-1*H*-pyrazole (**1b**) (120 mg, 1 mmol) afforded mixture of **2b, 3b, 4b, 5b** (240 mg) in brown solid.

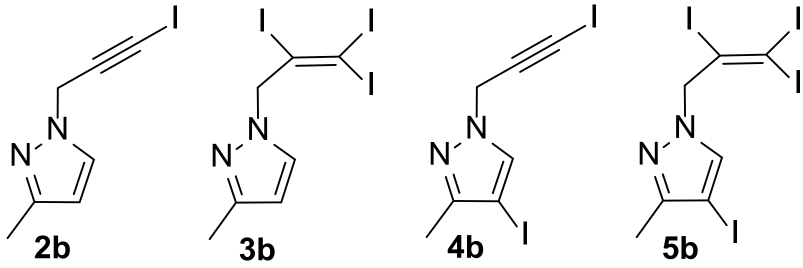

2b **3b** **4b** **5b**

- *1-(3-iodoprop-2-yn-1-yl)-3-methyl-1H-pyrazole (**2b)***
  ^{1}H NMR (300.086 MHz, $CDCl_3$) δ=7.43 (d, J = 2.3 Hz, 1H; H-5, Pz), 6.05 (d, J = 2.3 Hz, 1H; H-4, Pz), 5.00 (s, 2H; CH_2N), 2.27 (s, 3H; CH_3); ^{13}C NMR (100.618 MHz, $CDCl_3$) δ=149.3 (3-C; Pz), 129.7 (5-C; Pz), 105.9 (4-C; Pz), 87.2 (CCH_2), 42.9 (CH_2), 13.57 (CH_3), 4.35 (CI).
- *3-methyl-1-(2,3,3-triiodoallyl)-1H-pyrazole (**3b)***
  ^{1}H NMR (300.086 MHz, $CDCl_3$) δ=7.32 (d, J = 2.3 Hz, 1H; H-5, Pz), 6.07 (d, J = 2.3 Hz, 1H; H-4, Pz), 4.98 (s, 2H; CH_2N); 2.29 (s, 3H; CH_3); ^{13}C NMR (100.618

MHz, $CDCl_3$) δ=149.5 (3-C; Pz), 130.5 (5-C; Pz), 113.1 (=CI), 106.2 (4-C; Pz), 66.5 (CH_2), 24.3 (=CI_2), 13.59 (CH_3).

- *4-iodo-1-(3-iodoprop-2-yn-1-yl)-3-methyl-1H-pyrazole (**4b**)*

  ^{1}H NMR (300.086 MHz, $CDCl_3$) δ=7.52 (s, 1H; H-5, Pz), 4.99 (s, 2H; CH_2N), 2.23 (s, 3H; 3-CH_3); ^{13}C NMR (100.618 MHz, $CDCl_3$) δ=151.6 (3-C; Pz), 134.0 (5-C; Pz), 86.5 (CCH_2), 60.1 (4-C; Pz), 43.5 (CH_2), 13.71 (CH_3), 5.5 (CI).

- *4-iodo-3-methyl-1-(2,3,3-triiodoallyl)-1H-pyrazole (**5b**)*

  ^{1}H NMR (300.086 MHz, $CDCl_3$) δ=7.42 (s, 1H; H-5, Pz), 4.99 (s, 2H; CH_2N), 2.26 (s, 3H; 3-CH_3); ^{13}C NMR (100.618 MHz, $CDCl_3$) δ=151.7 (3-C; Pz), 134.6 (5-C; Pz), 111.8 (=CI), 60.7 (4-C, Pz), 67.0 (CH_2), 25.6 (=CI_2), 13.69 (CH_3).

2a

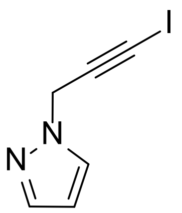
I
N
N

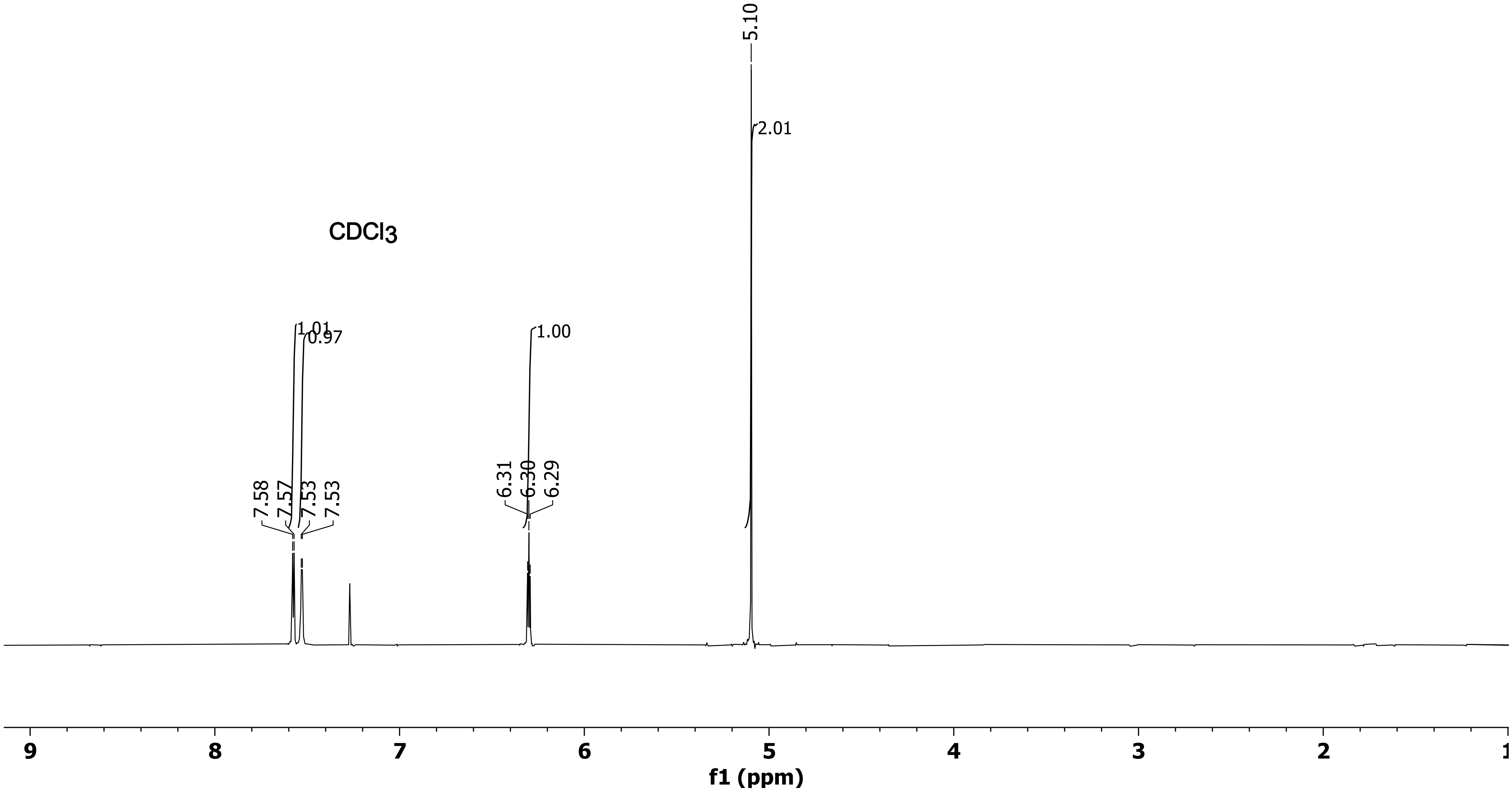
5.10
2.01
CDCl3
1.01
0.97
1.00
7.58
7.57
7.53
7.53
6.31
6.30
6.29
9
8
7
6
5
4
3
2
f1 (ppm)

2a

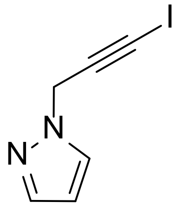
N
N
I

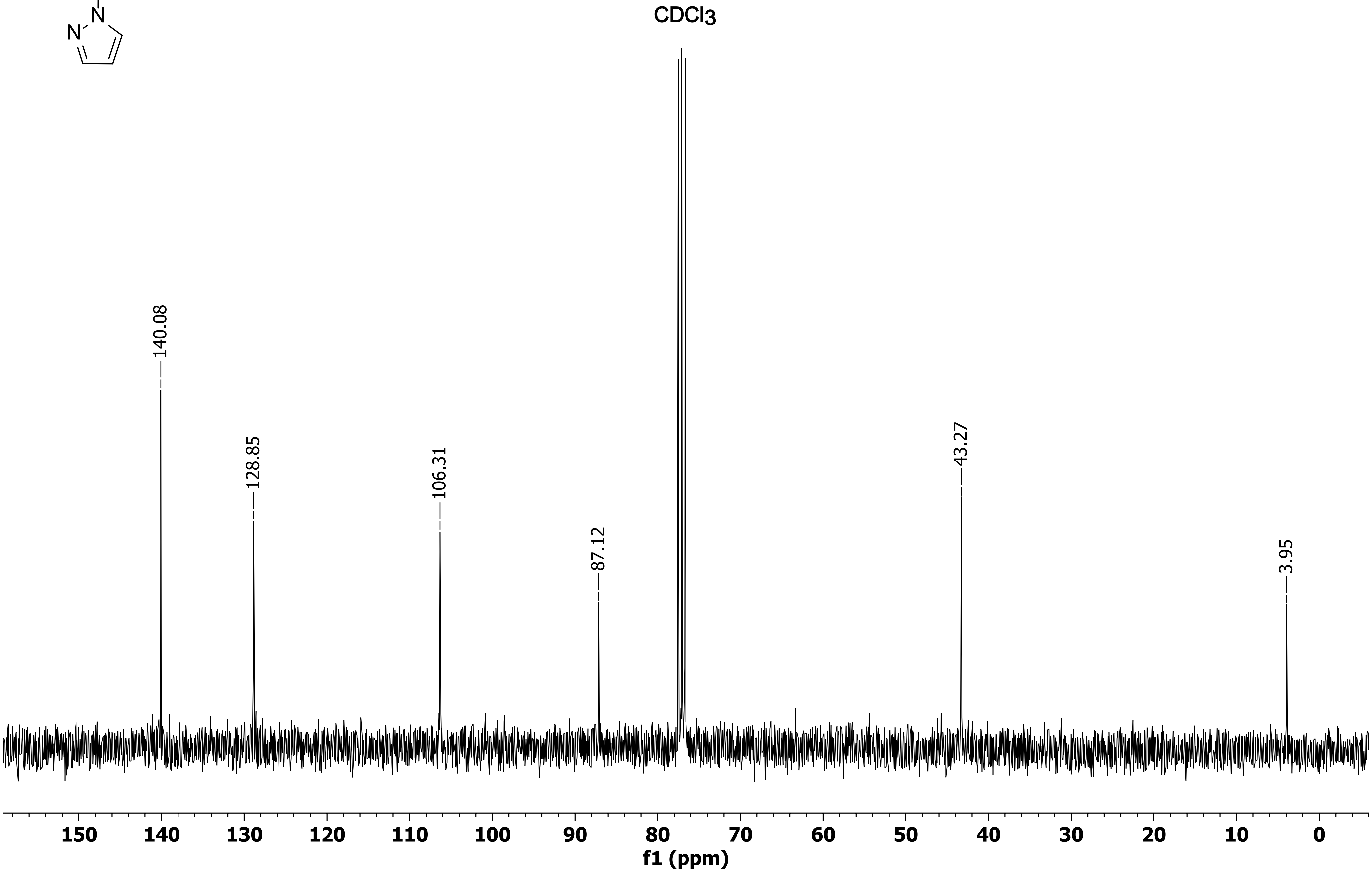
CDCl3
140.08
128.85
106.31
87.12
43.27
3.95
150
140
130
120
110
100
90
80
70
60
50
40
30
20
10
0
f1 (ppm)

2b

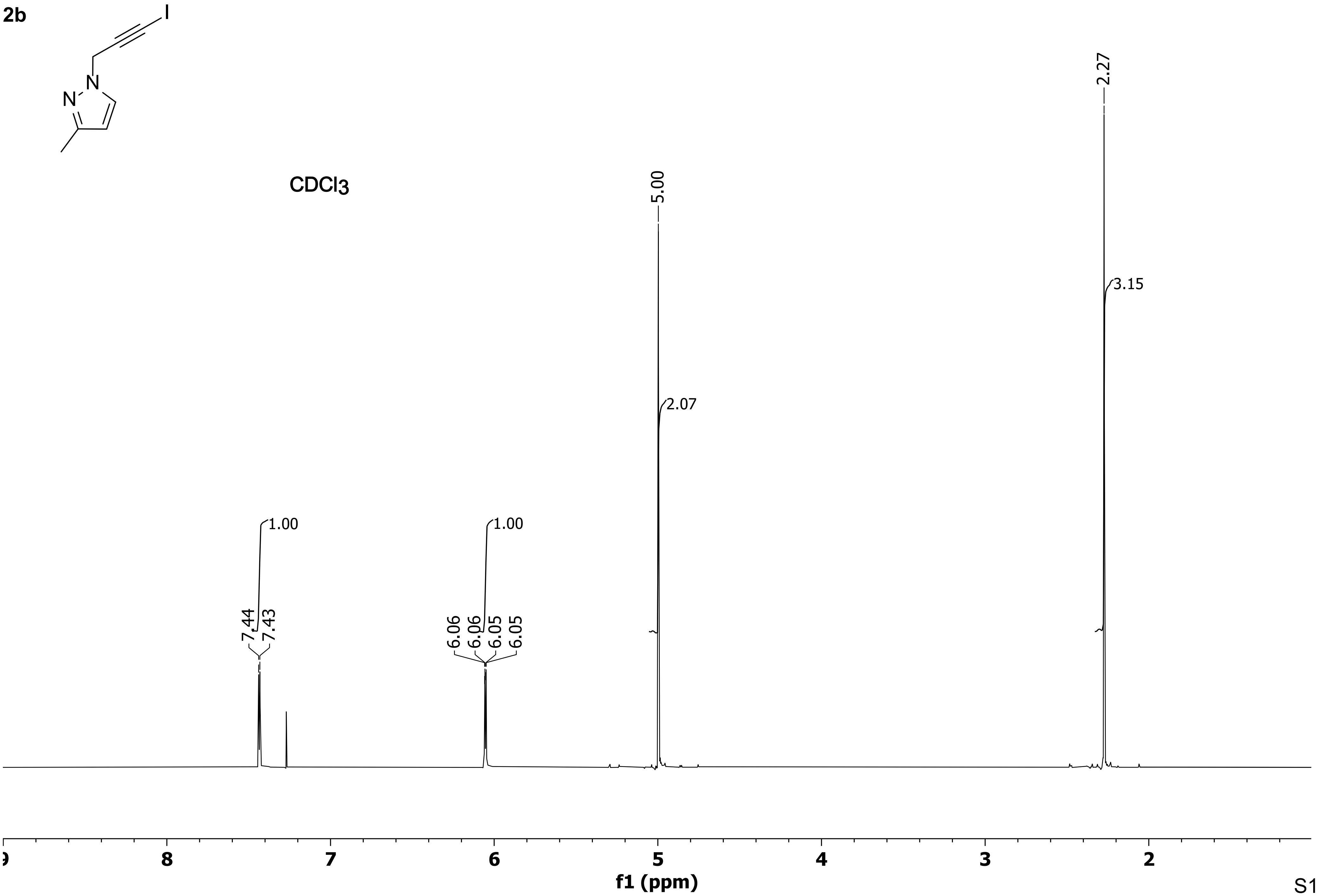
N
N
I
CDCl3
5.00
2.27
3.15
2.07
1.00
1.00
7.44
7.43
6.06
6.06
6.05
6.05
9
8
7
6
5
4
3
2
f1 (ppm)

2b

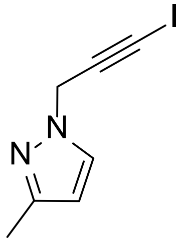
I
N
N

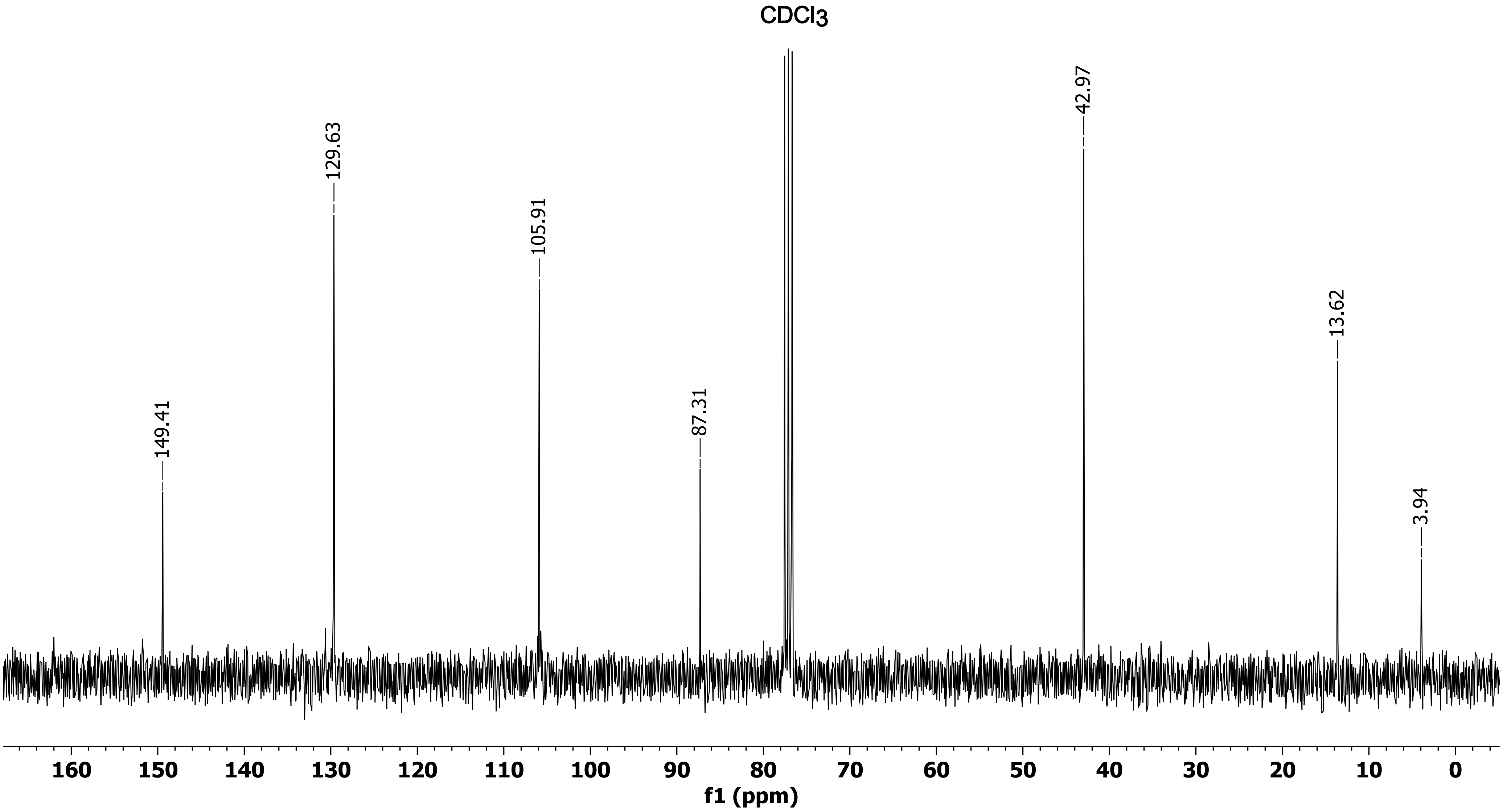
CDCl3
149.41
129.63
105.91
87.31
42.97
13.62
3.94
160 150 140 130 120 110 100 90 80 70 60 50 40 30 20 10 0
f1 (ppm)

2c

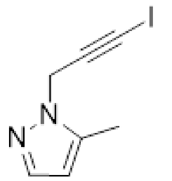
I
N
N

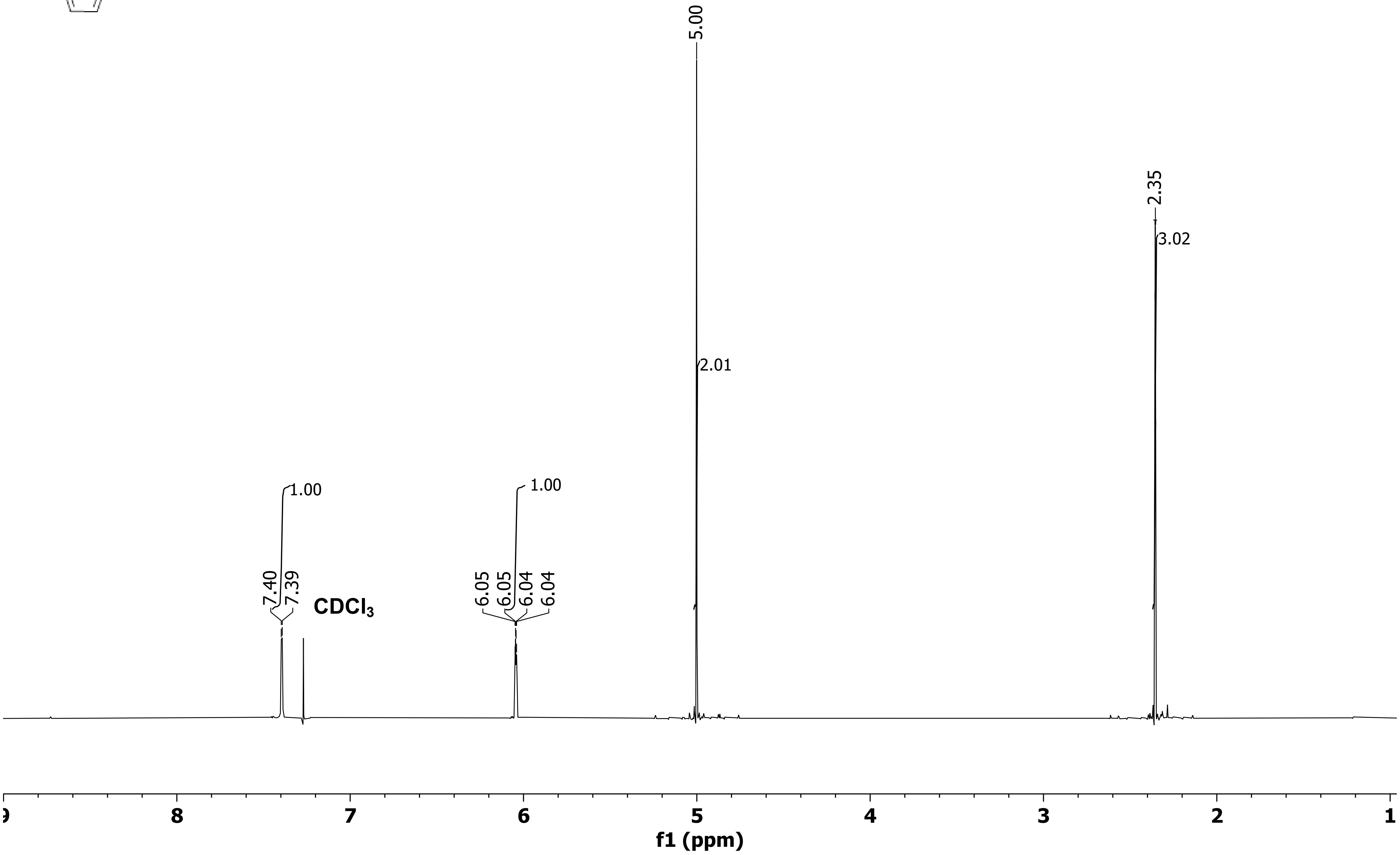
5.00
2.35
3.02
2.01
1.00
1.00
7.40
7.39
CDCl$_3$
6.05
6.05
6.04
6.04
9
8
7
6
5
4
3
2
1
f1 (ppm)

2c

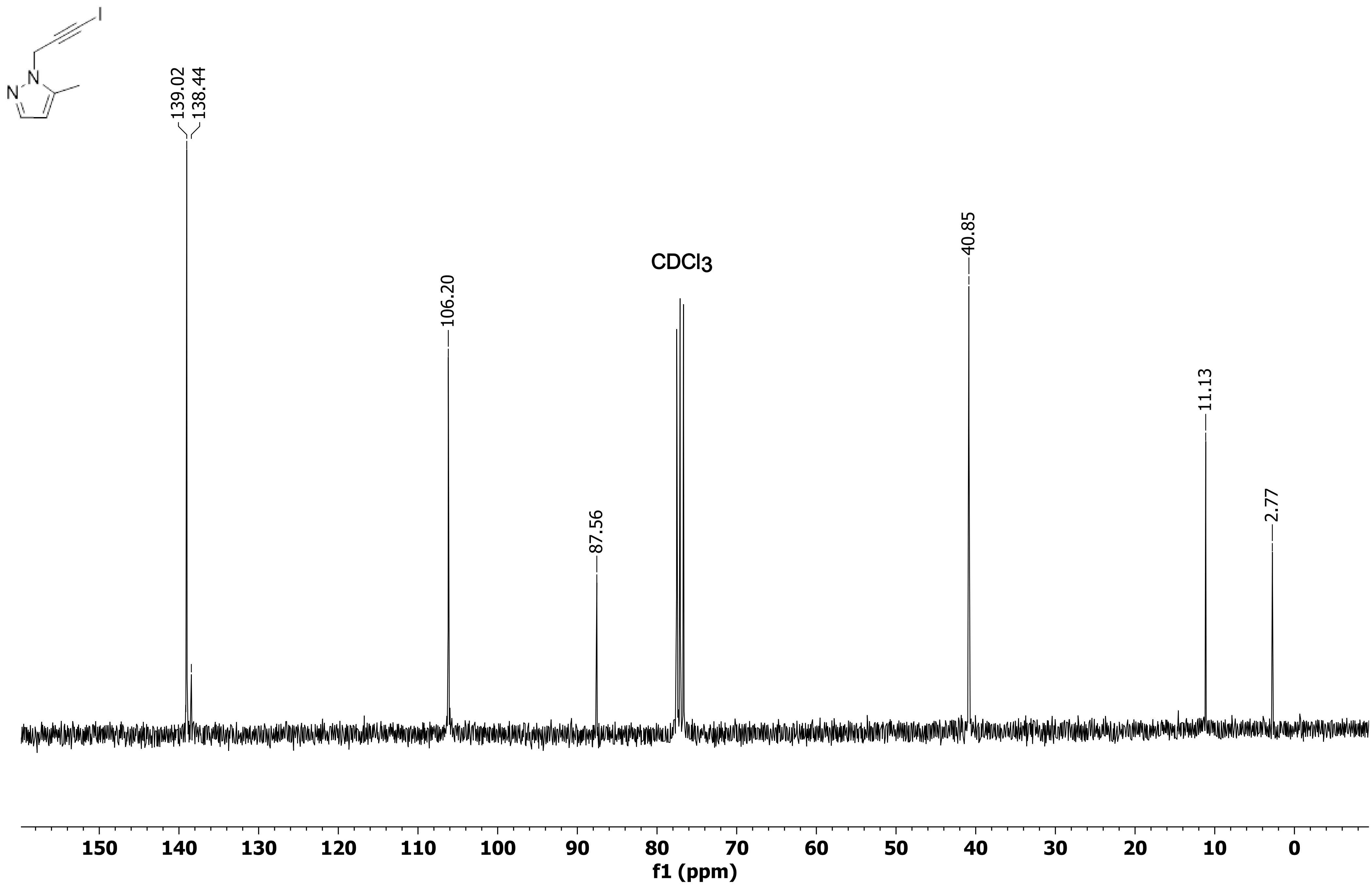

I
N
N
139.02
138.44
106.20
87.56
CDCl3
40.85
11.13
2.77
150
140
130
120
110
100
90
80
70
60
50
40
30
20
10
0
f1 (ppm)

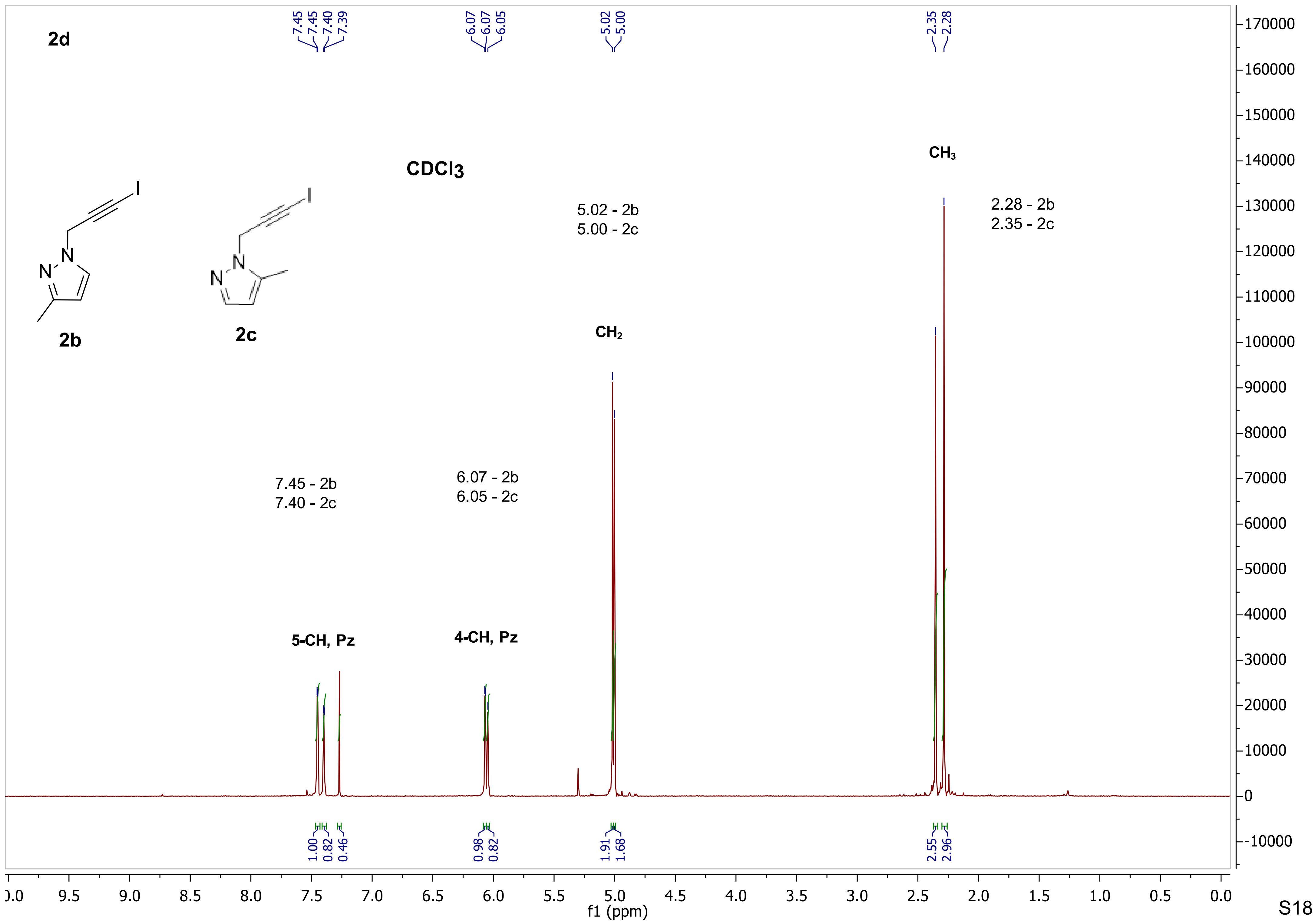
2d
7.45
7.45
7.40
7.39
6.07
6.07
6.05
5.02
5.00
2.35
2.28
CDCl3
CH3
5.02 - 2b
5.00 - 2c
2.28 - 2b
2.35 - 2c
2b
2c
CH2
7.45 - 2b
7.40 - 2c
6.07 - 2b
6.05 - 2c
5-CH, Pz
4-CH, Pz
1.00
0.82
0.46
0.98
0.82
1.91
1.68
2.55
2.96
f1 (ppm)

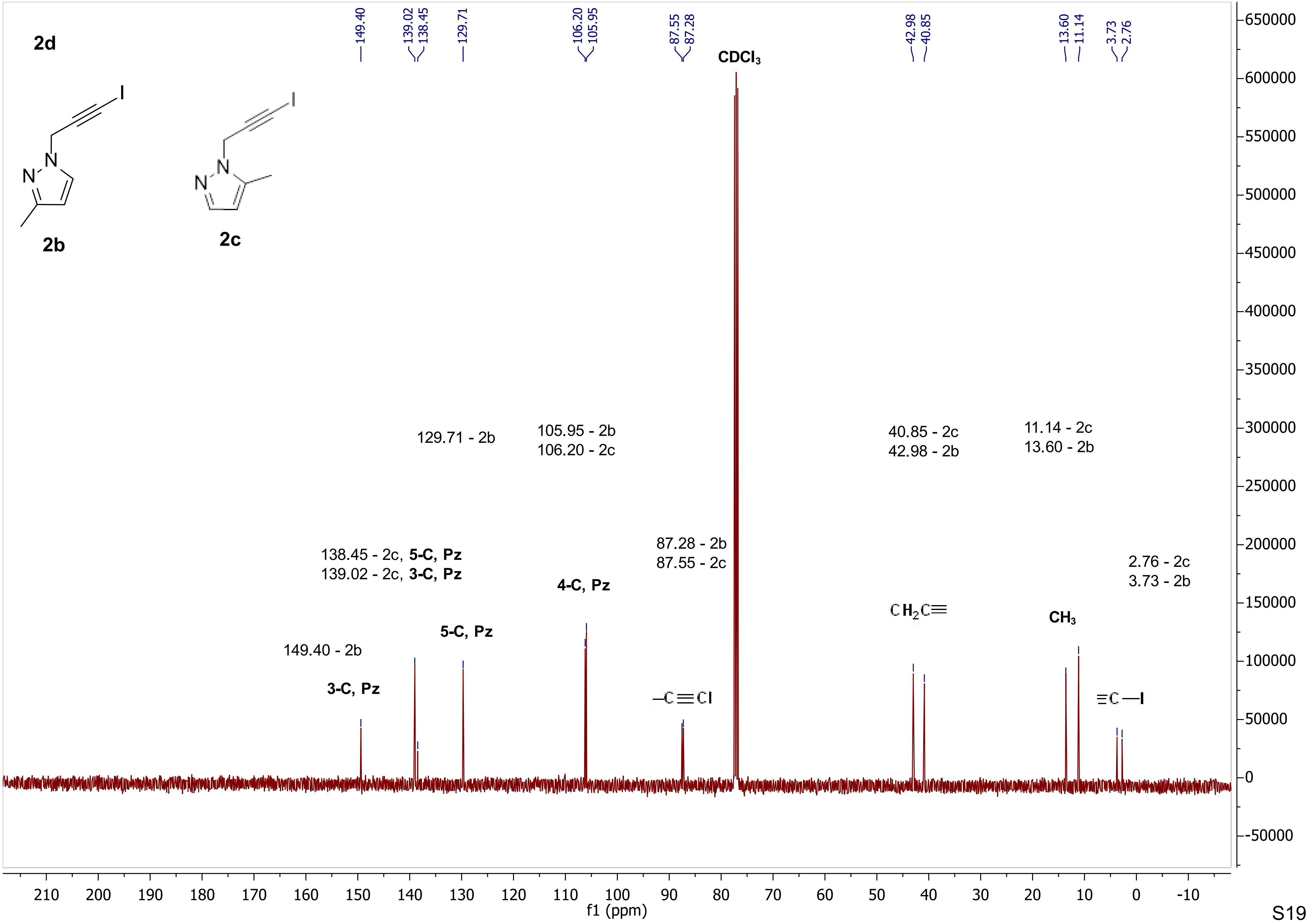
2d
2b
2c
149.40
139.02
138.45
129.71
106.20
105.95
87.55
87.28
CDCl3
42.98
40.85
13.60
11.14
3.73
2.76
129.71 - 2b
105.95 - 2b
106.20 - 2c
40.85 - 2c
42.98 - 2b
11.14 - 2c
13.60 - 2b
138.45 - 2c, 5-C, Pz
139.02 - 2c, 3-C, Pz
87.28 - 2b
87.55 - 2c
2.76 - 2c
3.73 - 2b
4-C, Pz
5-C, Pz
149.40 - 2b
3-C, Pz
CH2C≡
CH3
–C≡CI
≡C—I
f1 (ppm)

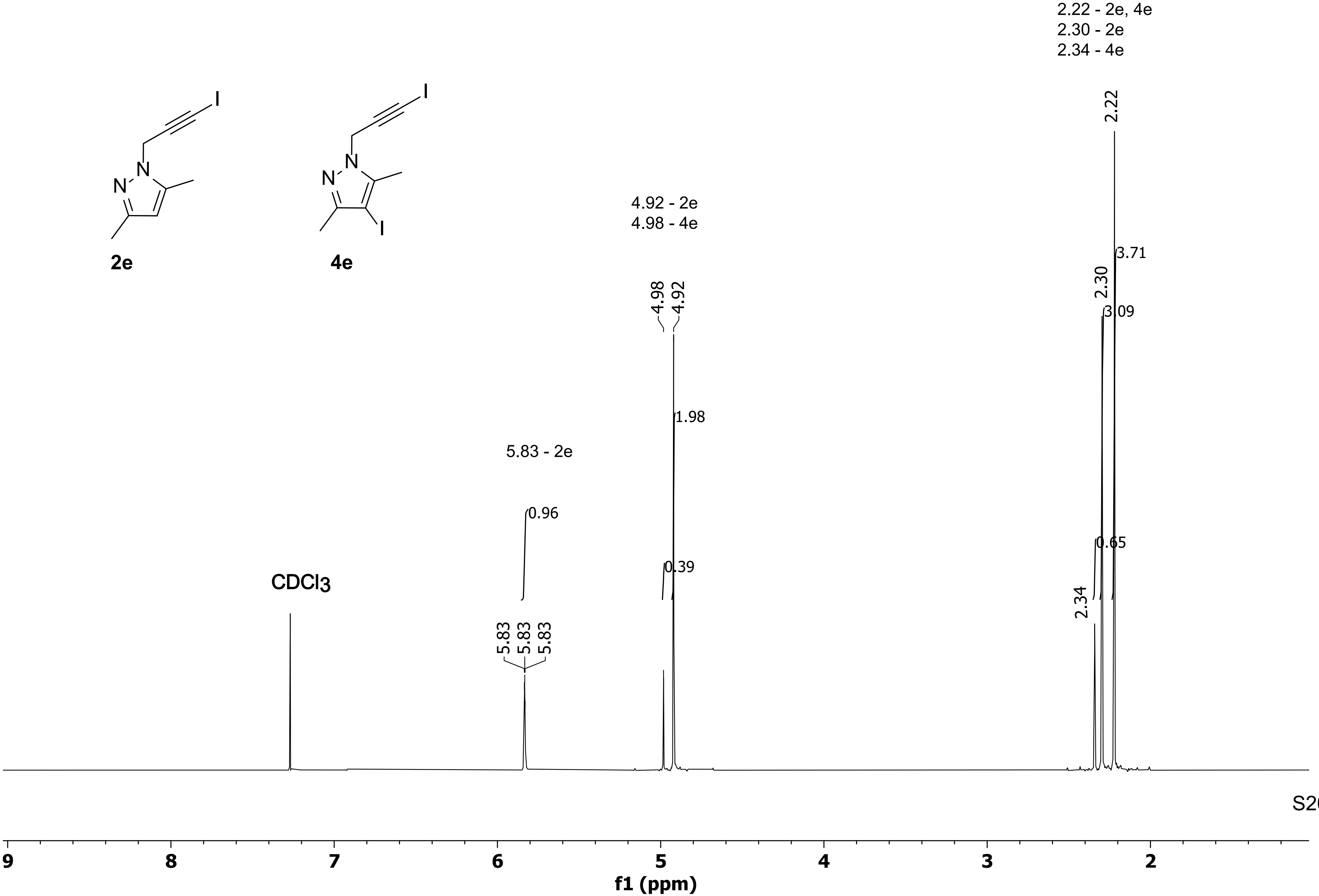
2e
4e
2.22 - 2e, 4e
2.30 - 2e
2.34 - 4e
4.92 - 2e
4.98 - 4e
5.83 - 2e
CDCl3
5.83
5.83
5.83
0.96
4.98
4.92
0.39
1.98
2.22
2.30
2.34
3.71
3.09
0.65
9
8
7
6
5
4
3
2
f1 (ppm)

2e

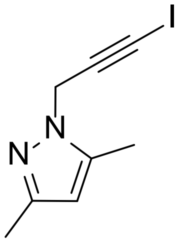

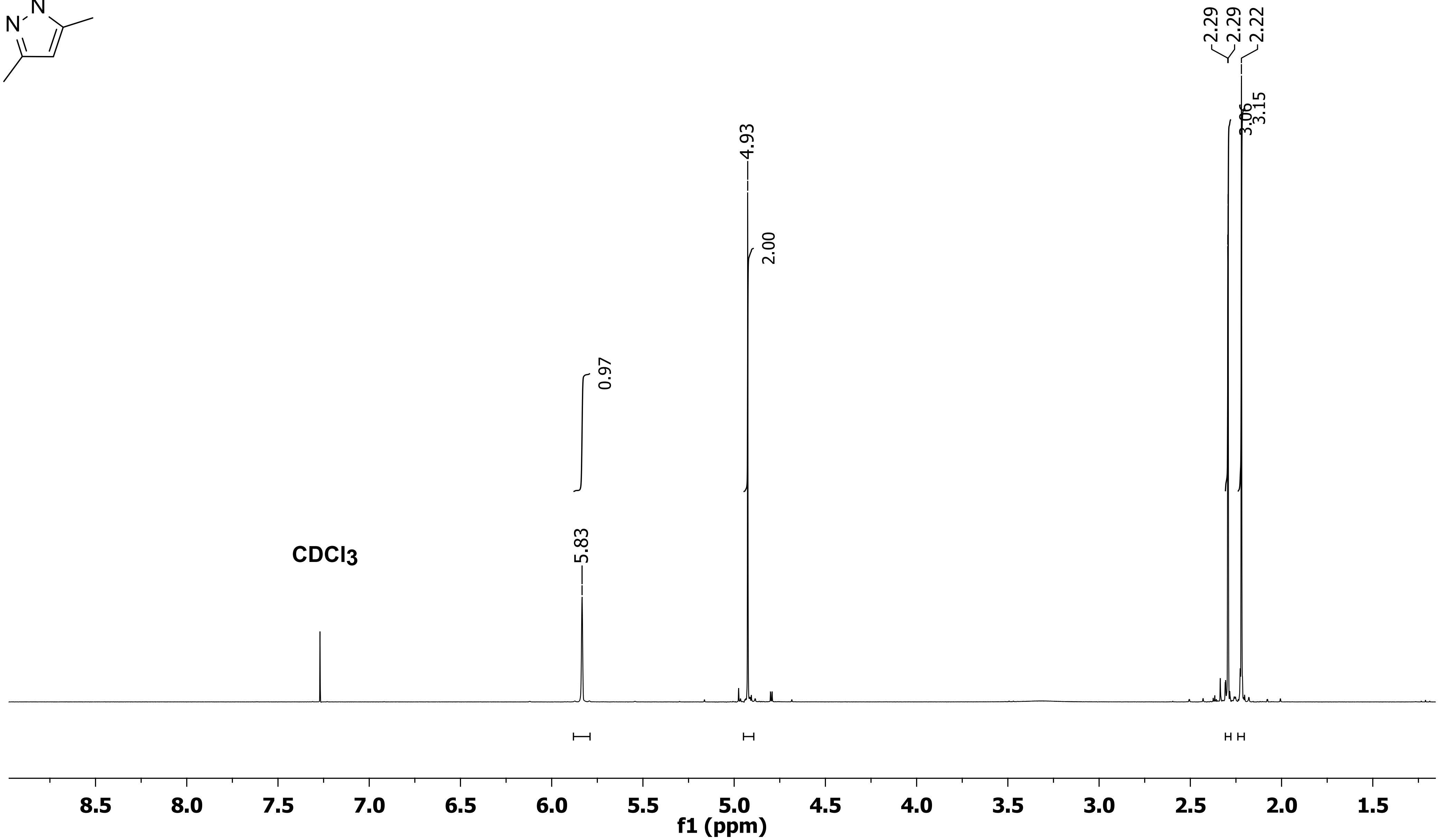
2.29
2.29
2.22
3.06
3.15
4.93
2.00
0.97
5.83
CDCl3
8.5
8.0
7.5
7.0
6.5
6.0
5.5
5.0
4.5
4.0
3.5
3.0
2.5
2.0
1.5
f1 (ppm)

2e

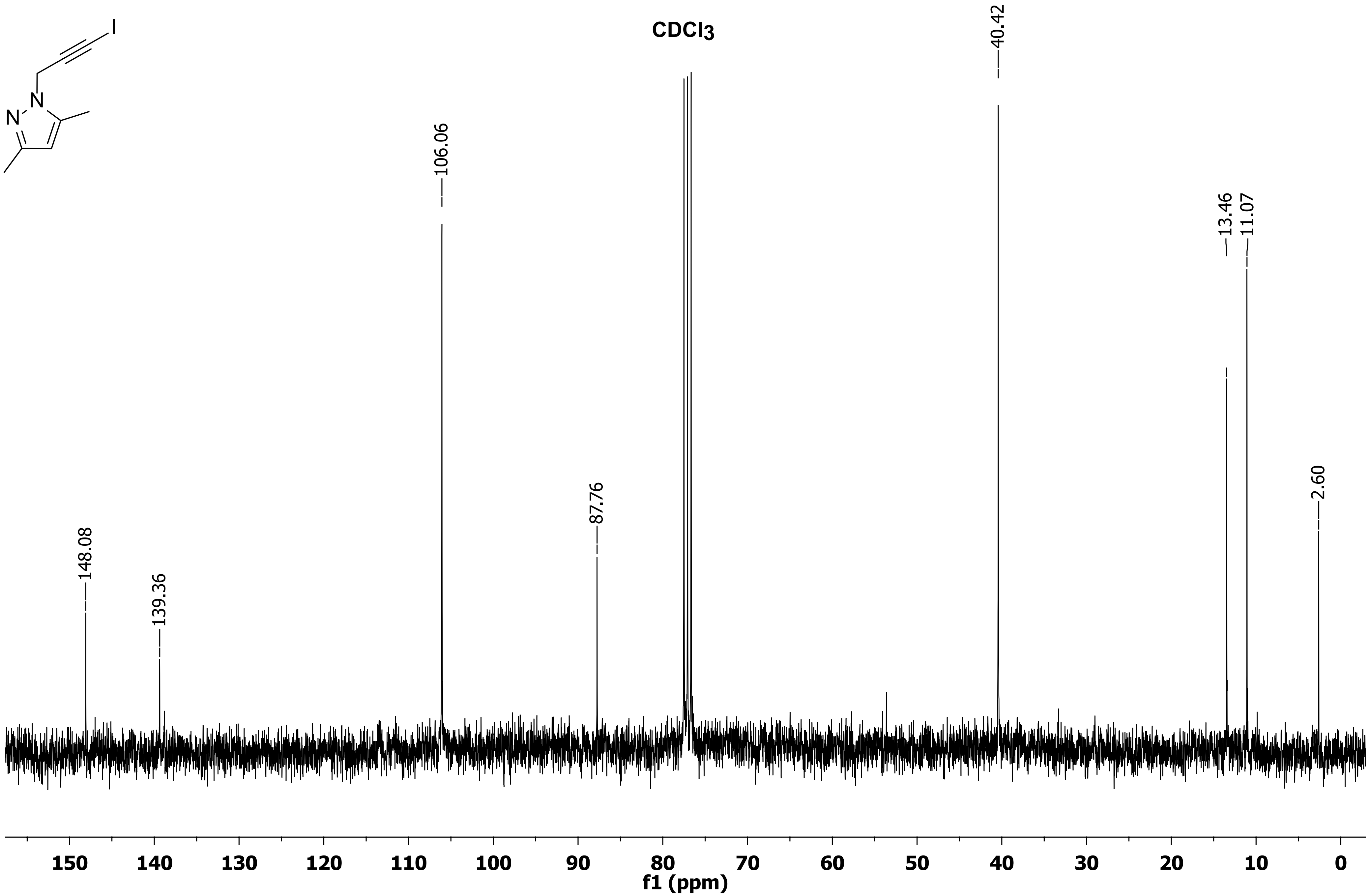

I
N
N
CDCl3
148.08
139.36
106.06
87.76
40.42
13.46
11.07
2.60
150
140
130
120
110
100
90
80
70
60
50
40
30
20
10
0
f1 (ppm)

3a

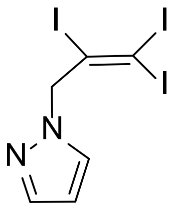

I
I
I
N
N

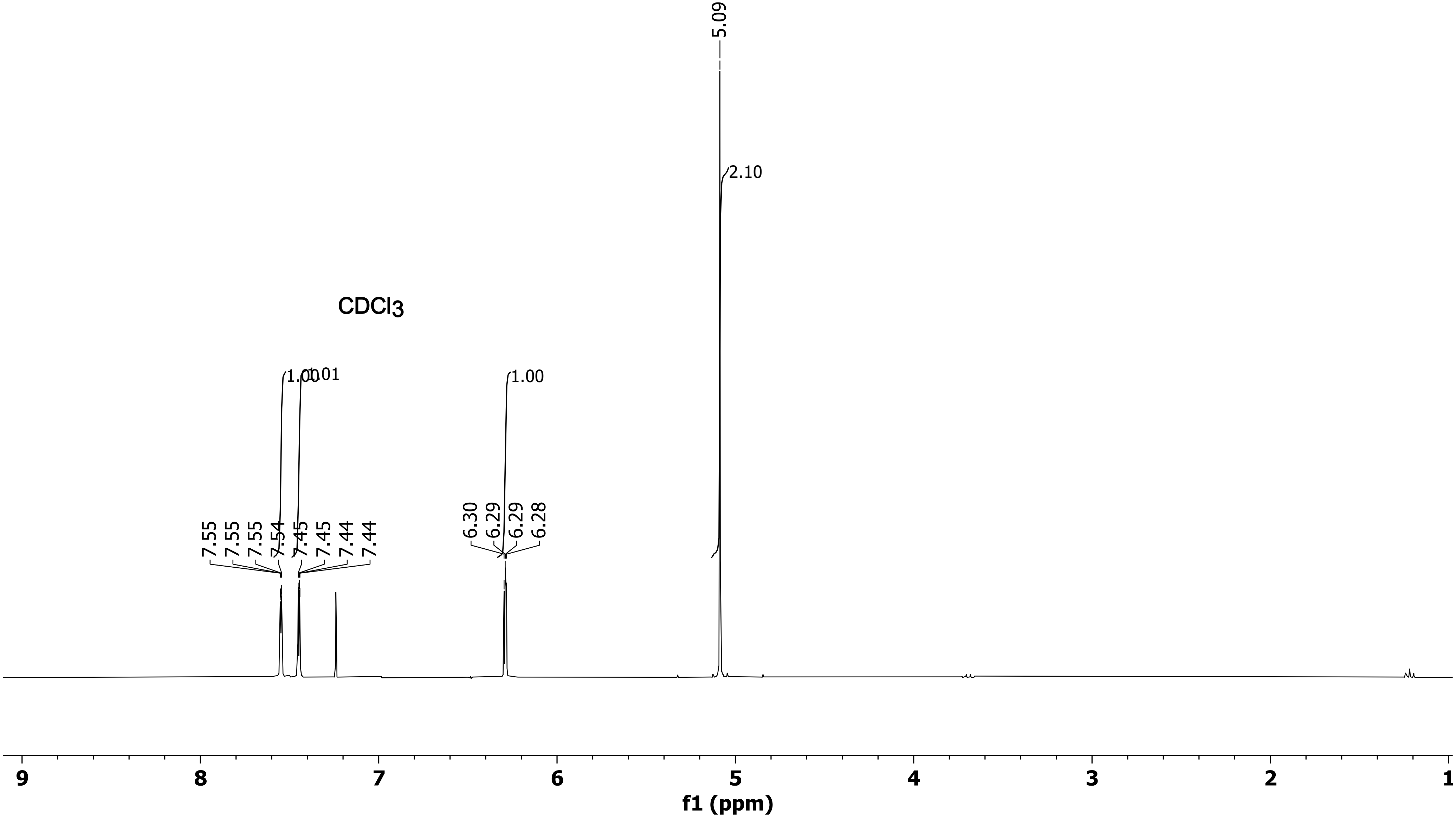

5.09
2.10
CDCl3
1.00
1.01
1.00
7.55
7.55
7.55
7.54
7.45
7.45
7.44
7.44
6.30
6.29
6.29
6.28
9
8
7
6
5
4
3
2
1
f1 (ppm)

3a

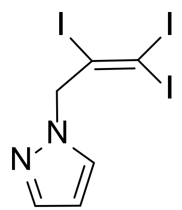

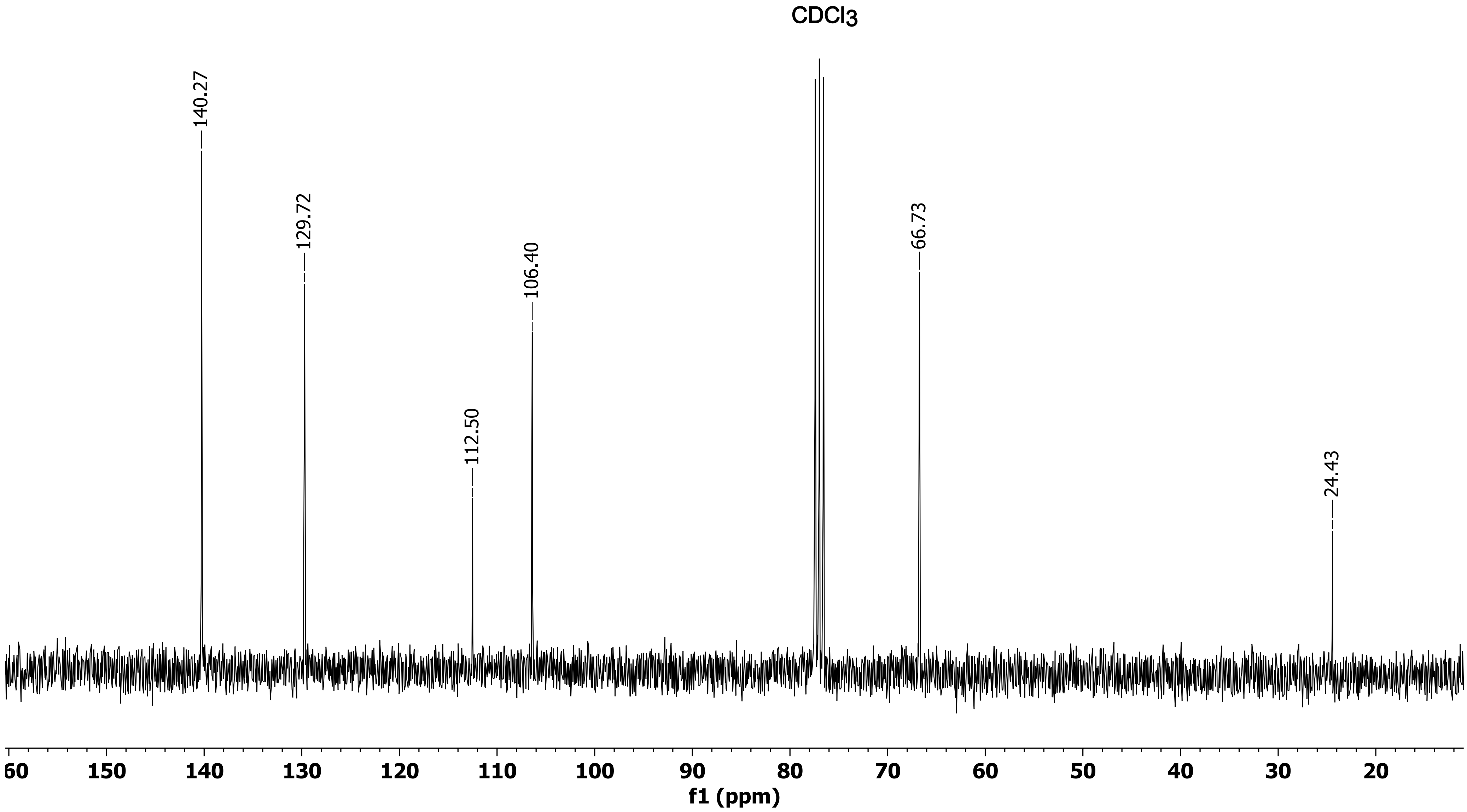

CDCl3
140.27
129.72
112.50
106.40
66.73
24.43
60
150
140
130
120
110
100
90
80
70
60
50
40
30
20
f1 (ppm)

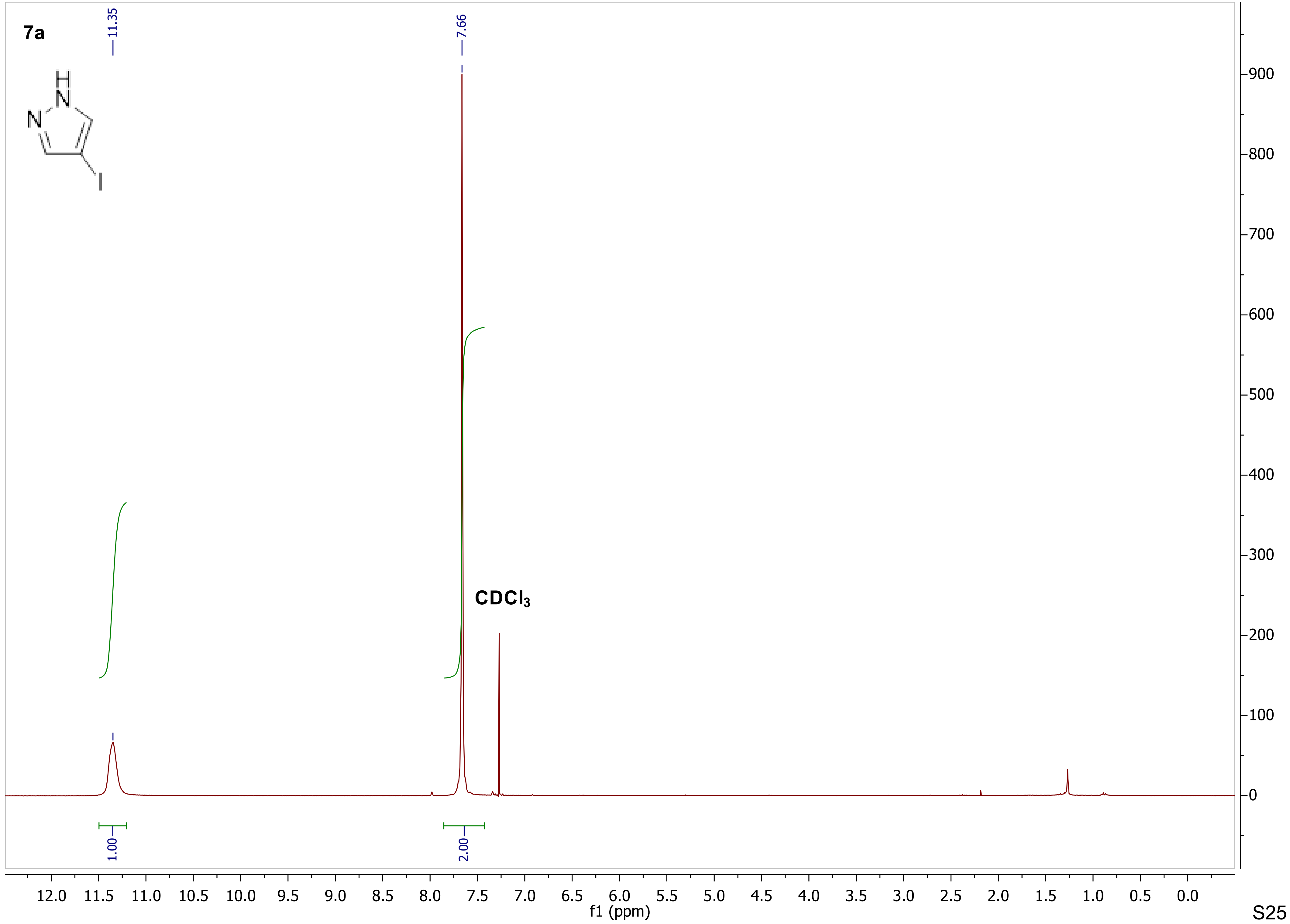
7a
11.35
7.66
CDCl3
1.00
2.00
f1 (ppm)

7a

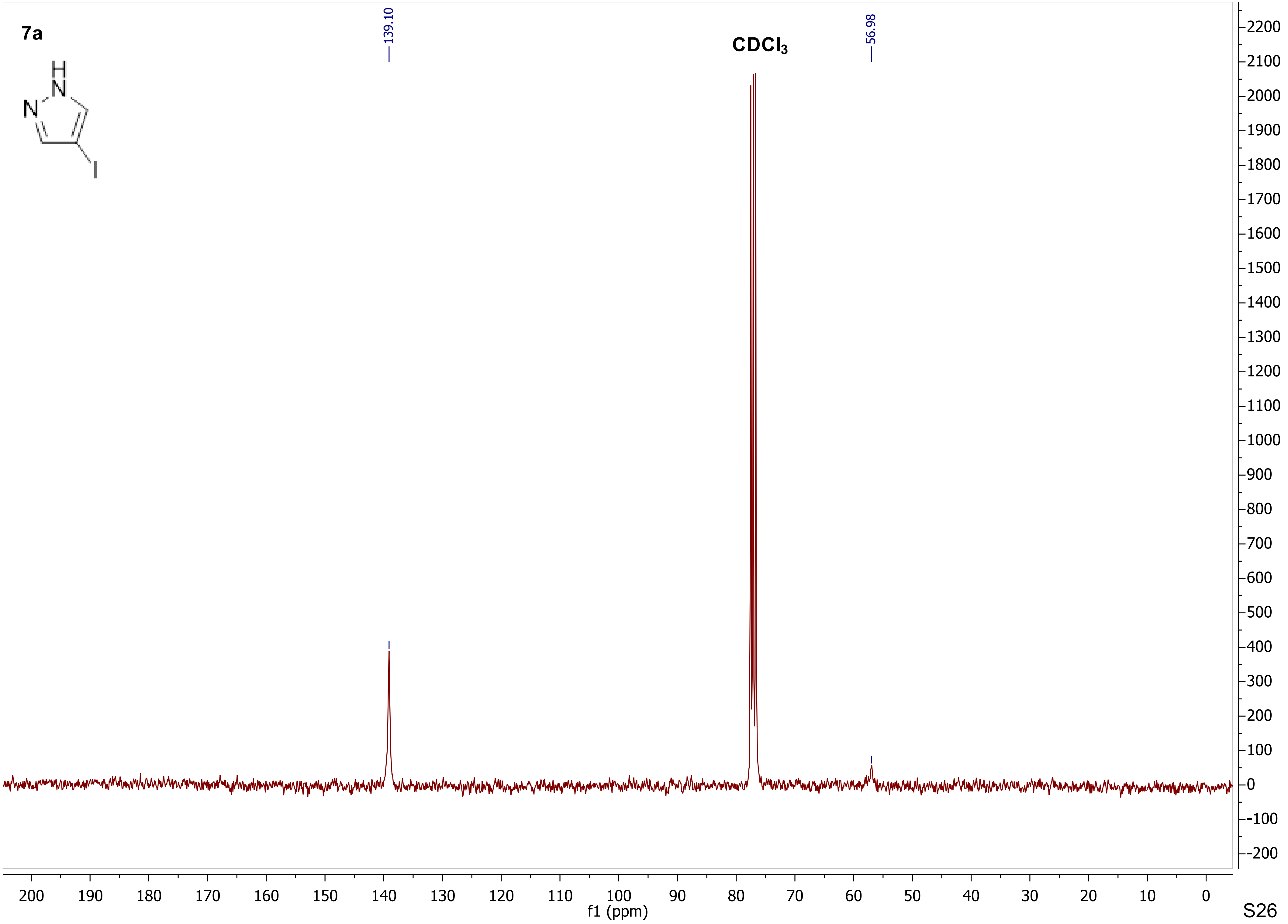

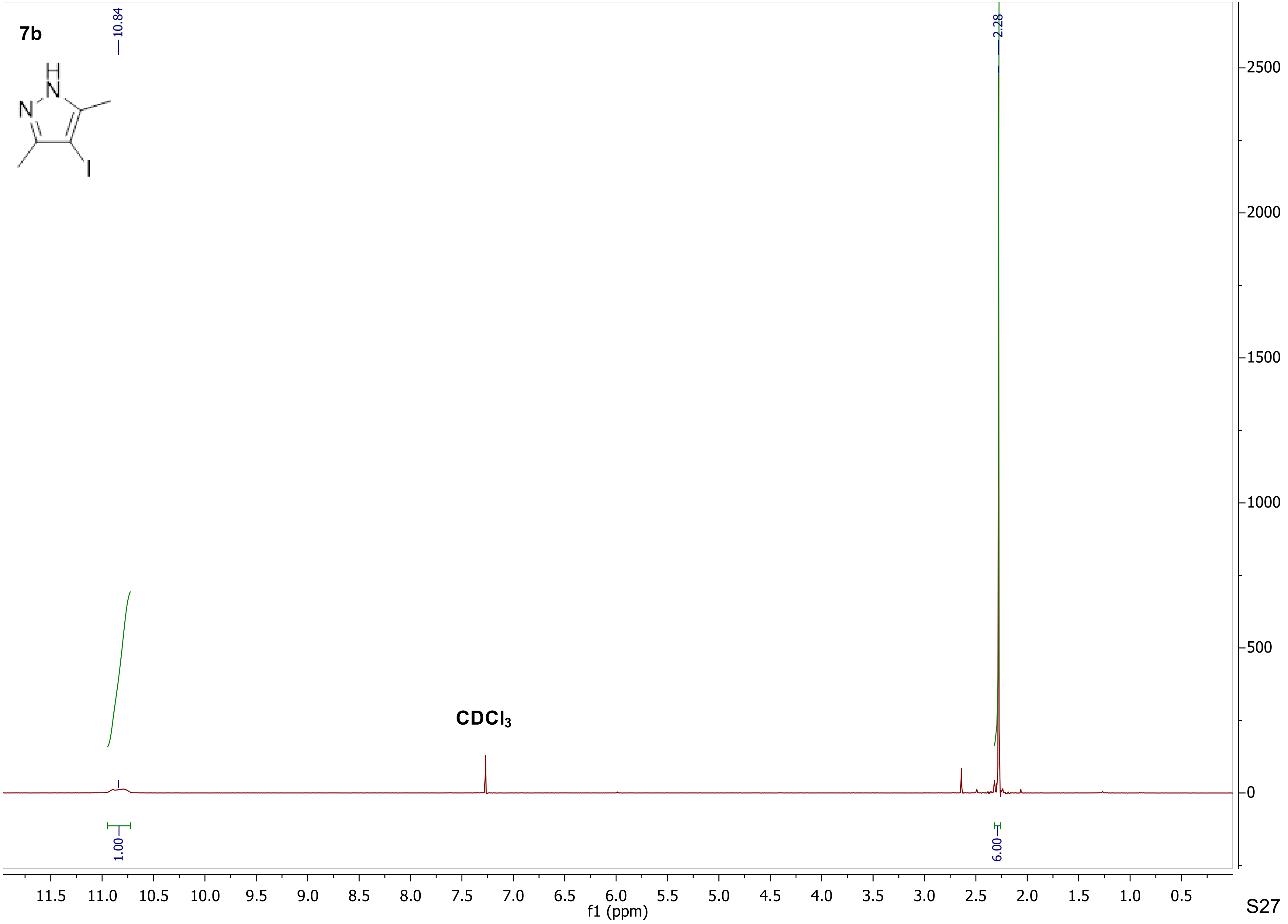
7b
10.84
2.28
CDCl3
1.00
6.00
11.5 11.0 10.5 10.0 9.5 9.0 8.5 8.0 7.5 7.0 6.5 6.0 5.5 5.0 4.5 4.0 3.5 3.0 2.5 2.0 1.5 1.0 0.5
f1 (ppm)
2500 2000 1500 1000 500 0

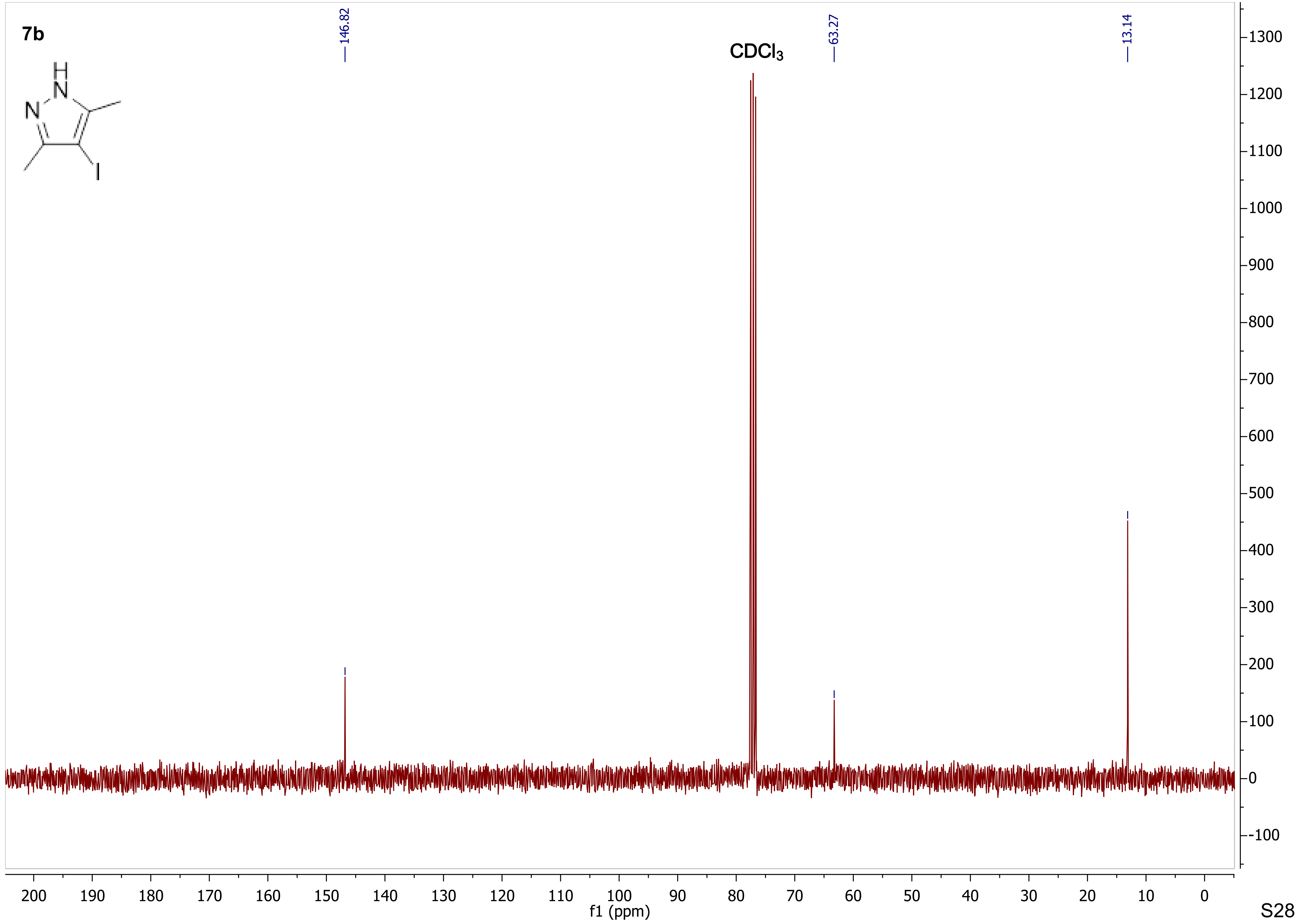

7b
CDCl3
146.82
63.27
13.14
f1 (ppm)

7c

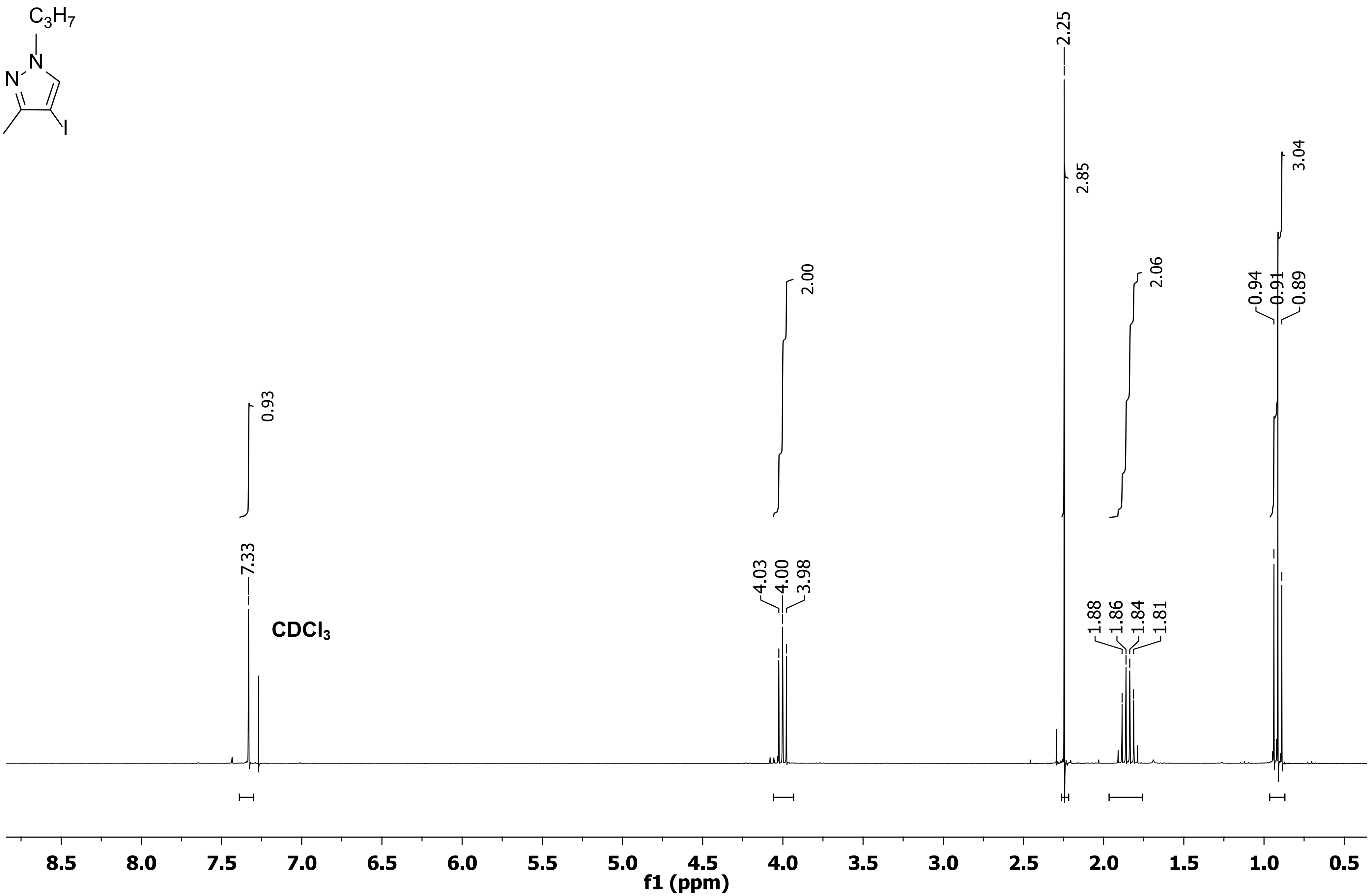

C_3H_7
N
N
I
2.25
2.85
3.04
2.00
2.06
0.94
0.91
0.89
0.93
7.33
4.03
4.00
3.98
1.88
1.86
1.84
1.81
$CDCl_3$
8.5 8.0 7.5 7.0 6.5 6.0 5.5 5.0 4.5 4.0 3.5 3.0 2.5 2.0 1.5 1.0 0.5
f1 (ppm)

7c

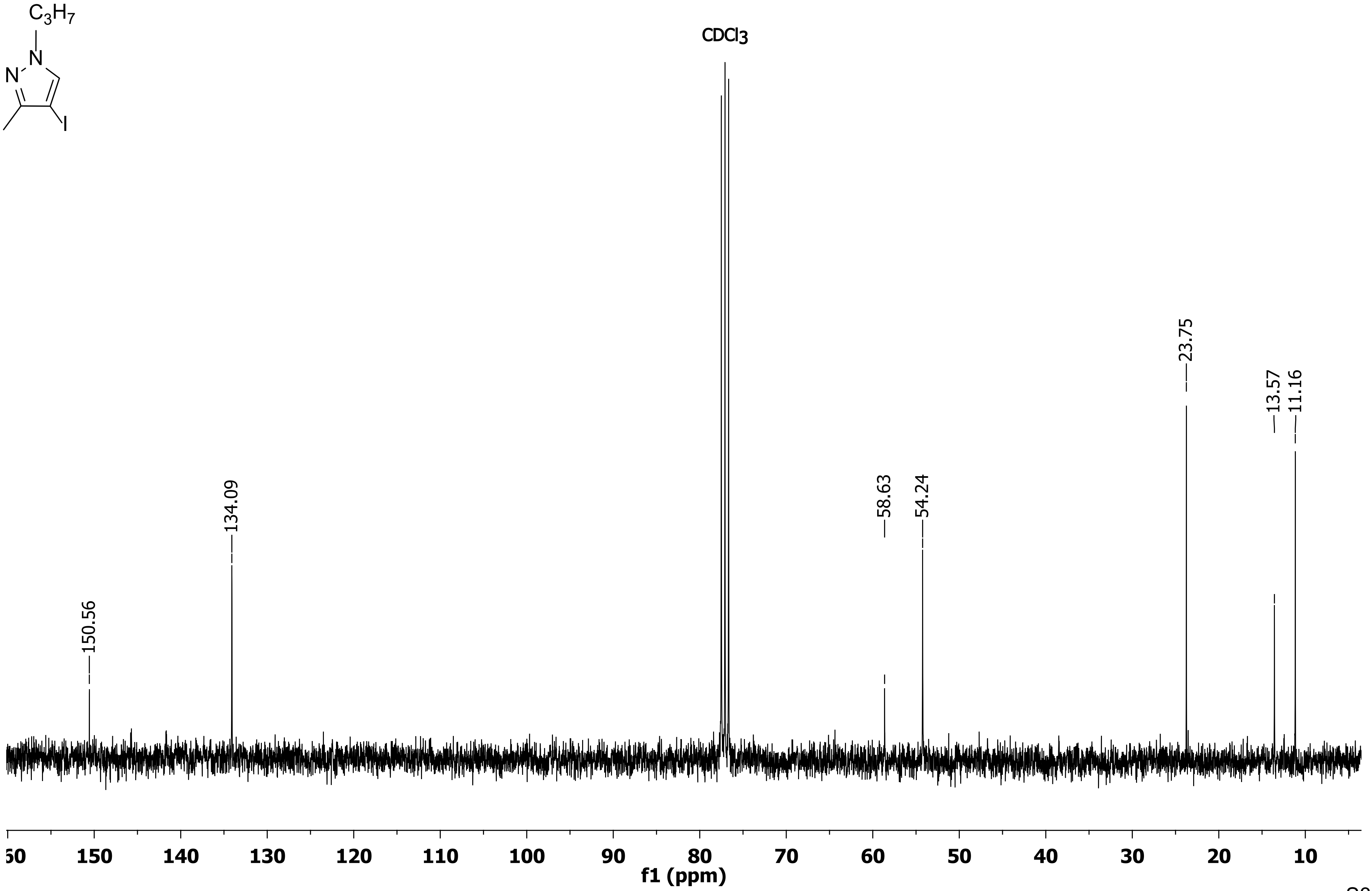
C3H7
N
N
I
CDCl3
150.56
134.09
58.63
54.24
23.75
13.57
11.16
150
140
130
120
110
100
90
80
70
60
50
40
30
20
10
f1 (ppm)

7d

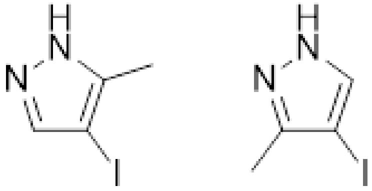

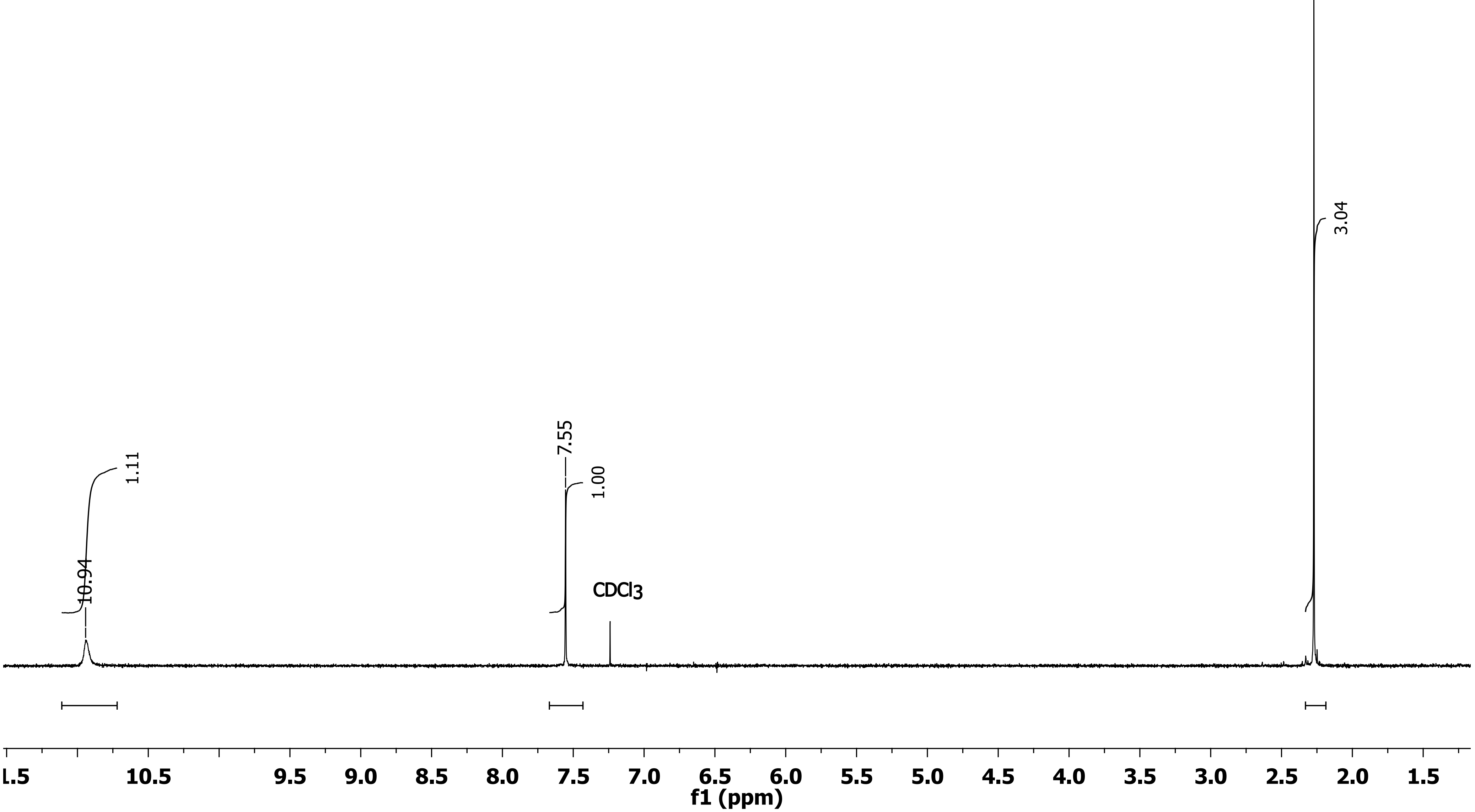

2.27
3.04
7.55
1.00
1.11
10.94
CDCl3
1.5 10.5 9.5 9.0 8.5 8.0 7.5 7.0 6.5 6.0 5.5 5.0 4.5 4.0 3.5 3.0 2.5 2.0 1.5
f1 (ppm)

7d

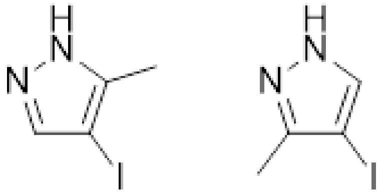

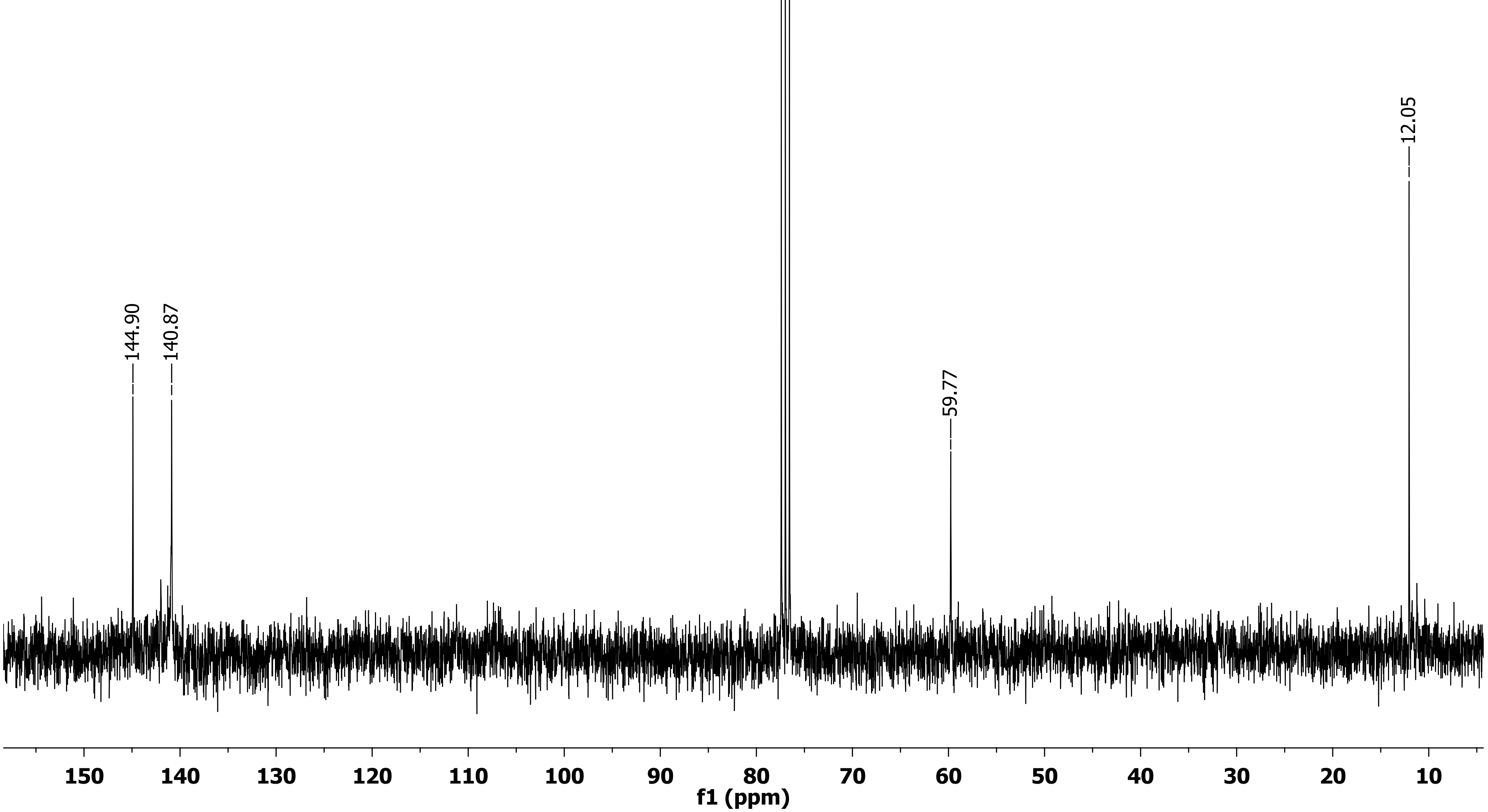
CDCl3
144.90
140.87
59.77
12.05
150
140
130
120
110
100
90
80
70
60
50
40
30
20
10
f1 (ppm)

7e

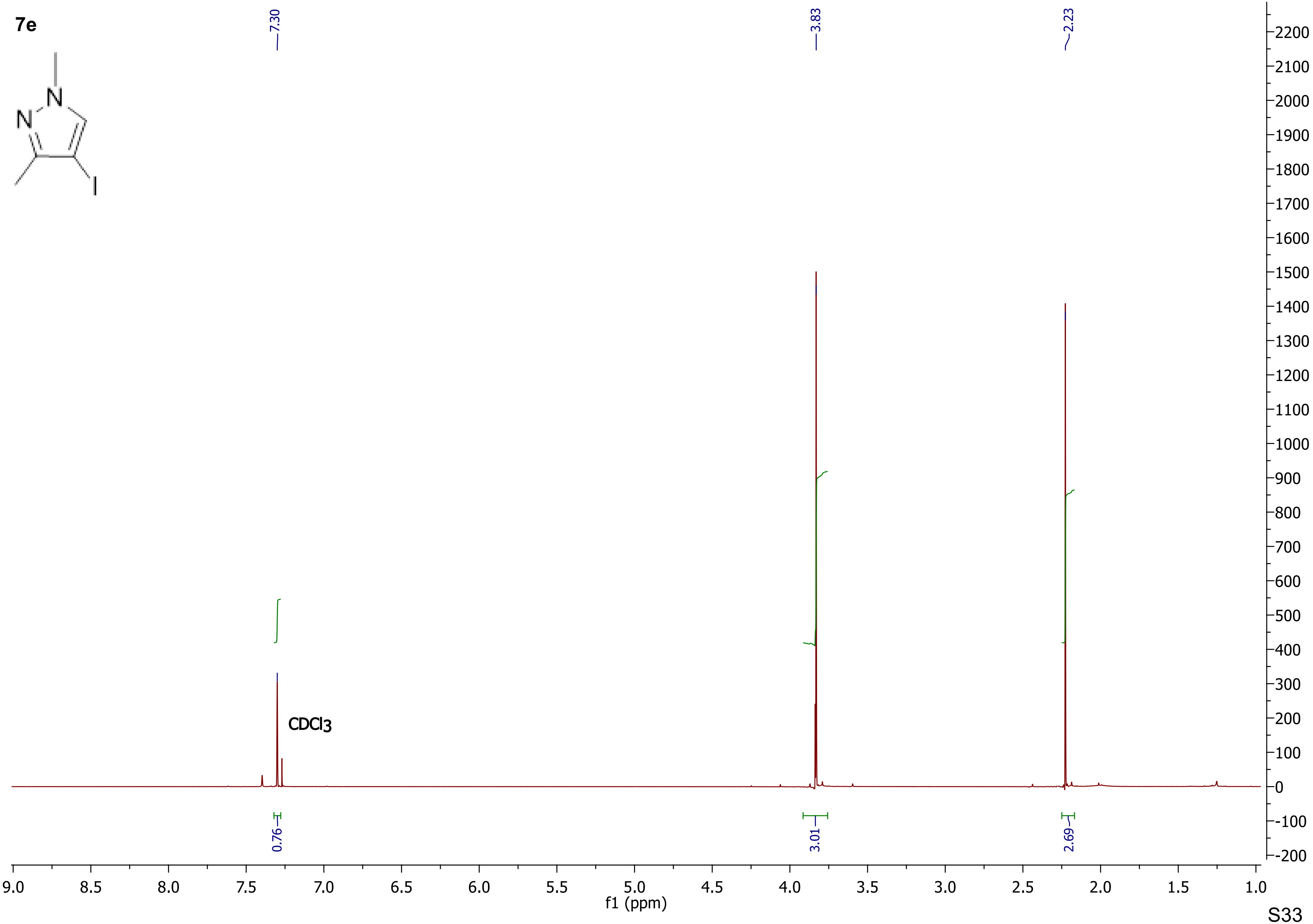

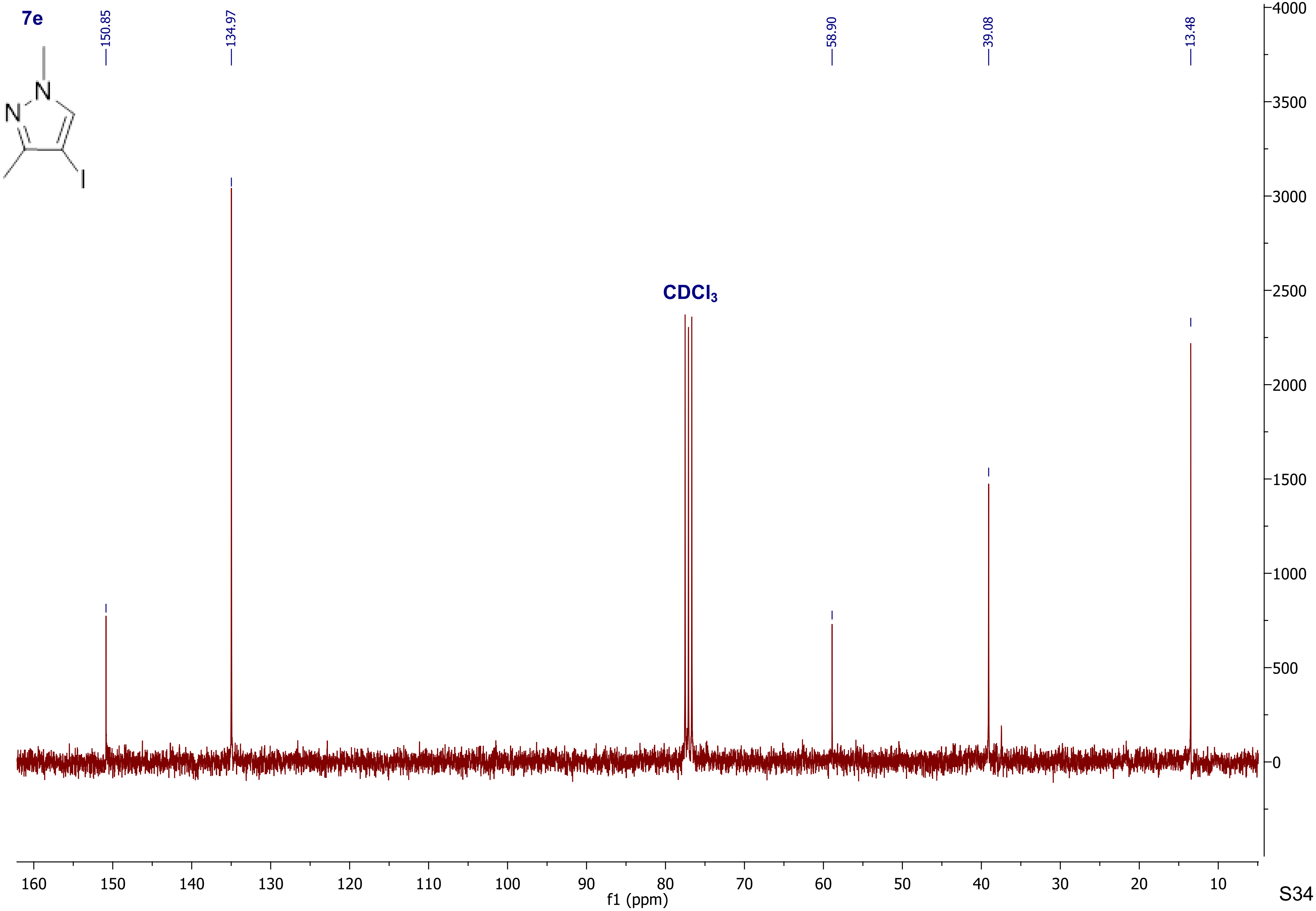
7e
N
N
I
150.85
134.97
58.90
39.08
13.48
CDCl3
4000
3500
3000
2500
2000
1500
1000
500
0
160
150
140
130
120
110
100
90
80
70
60
50
40
30
20
10
f1 (ppm)

7f

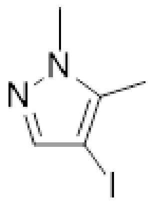

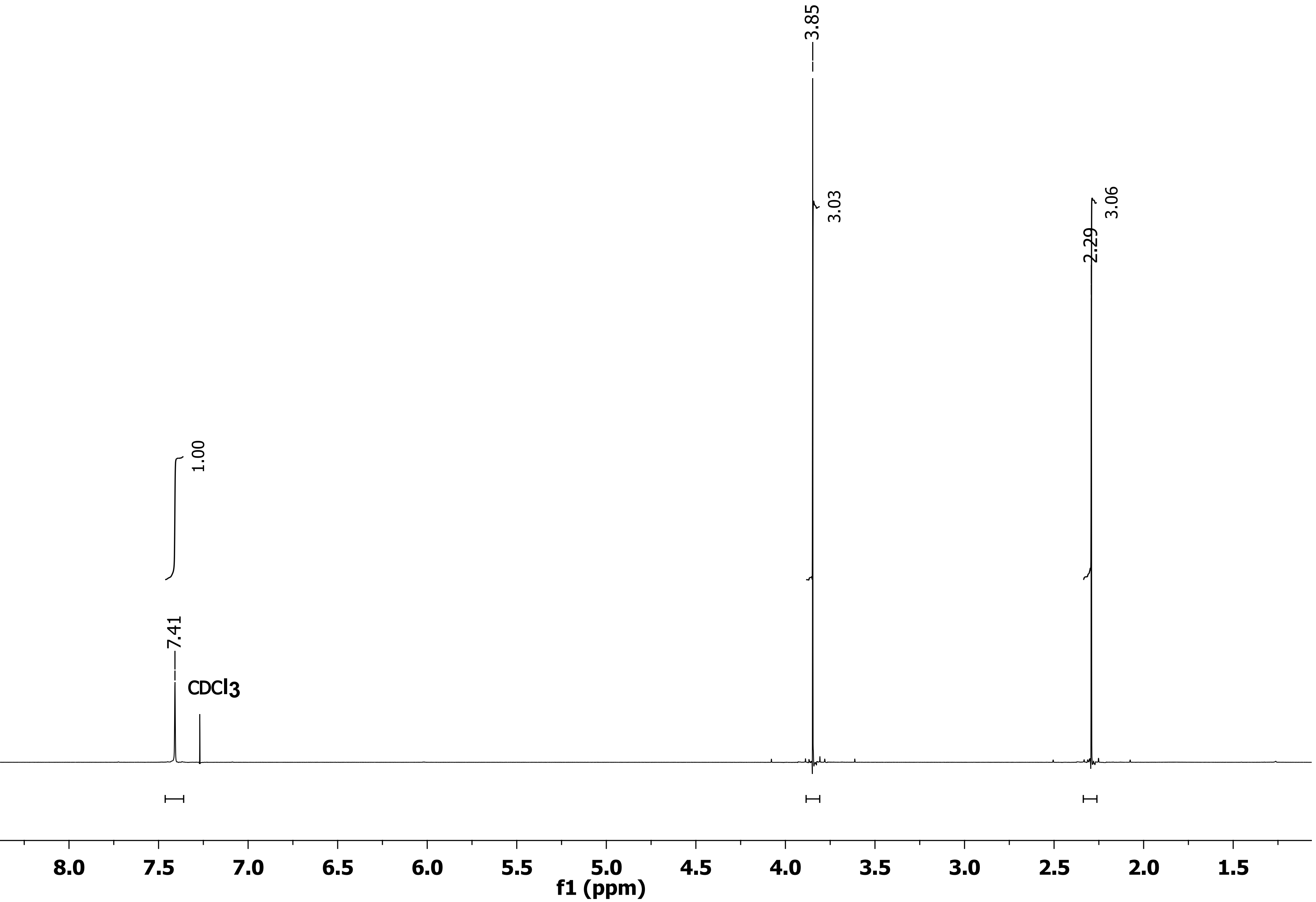

3.85
3.03
3.06
2.29
1.00
7.41
CDCl3
8.5
8.0
7.5
7.0
6.5
6.0
5.5
5.0
4.5
4.0
3.5
3.0
2.5
2.0
1.5
f1 (ppm)

7f

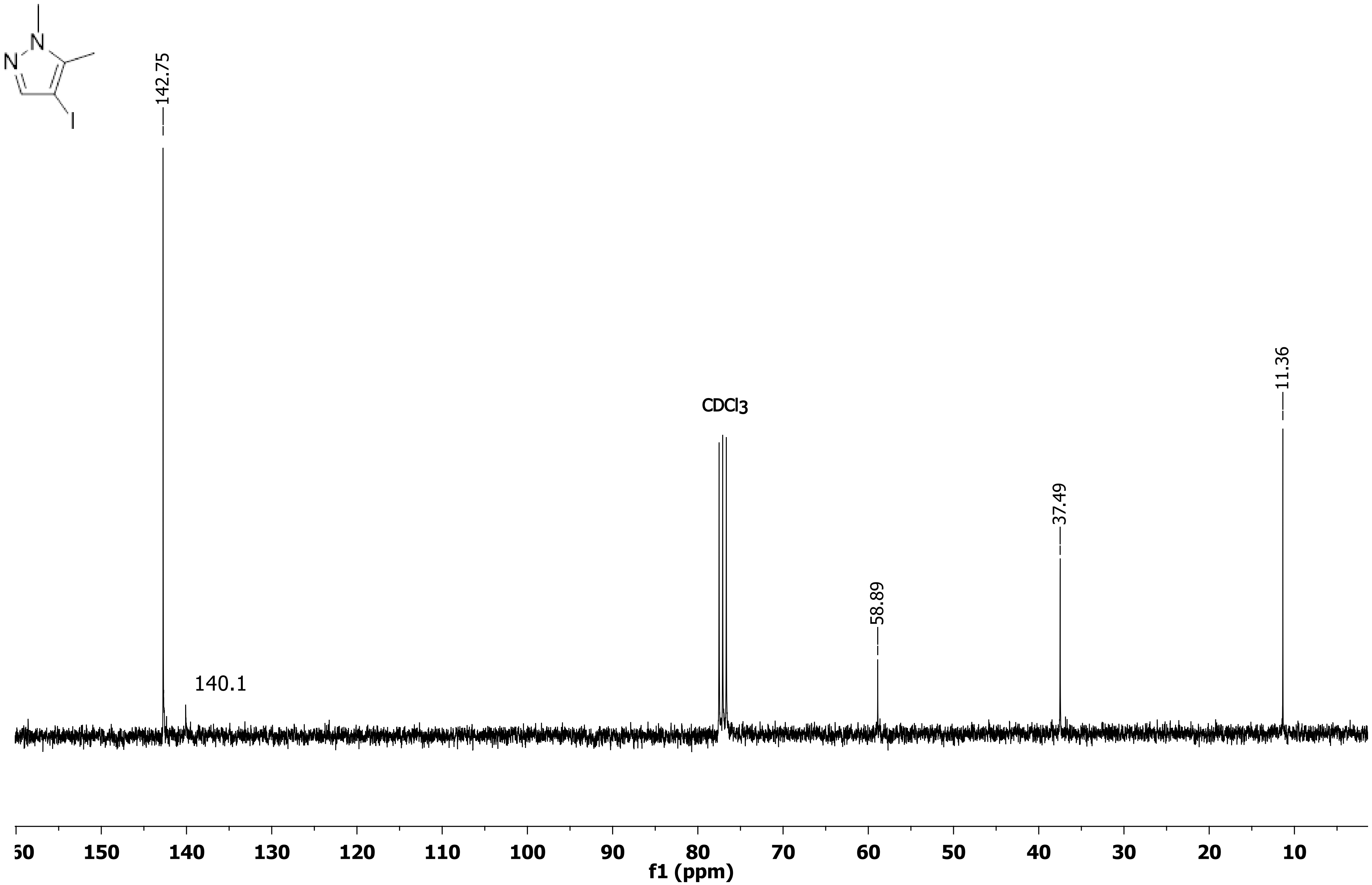

N
N
I
142.75
140.1
CDCl3
58.89
37.49
11.36
60
150
140
130
120
110
100
90
80
70
60
50
40
30
20
10
f1 (ppm)

4e

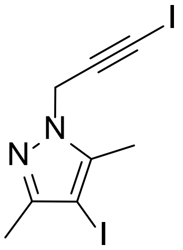

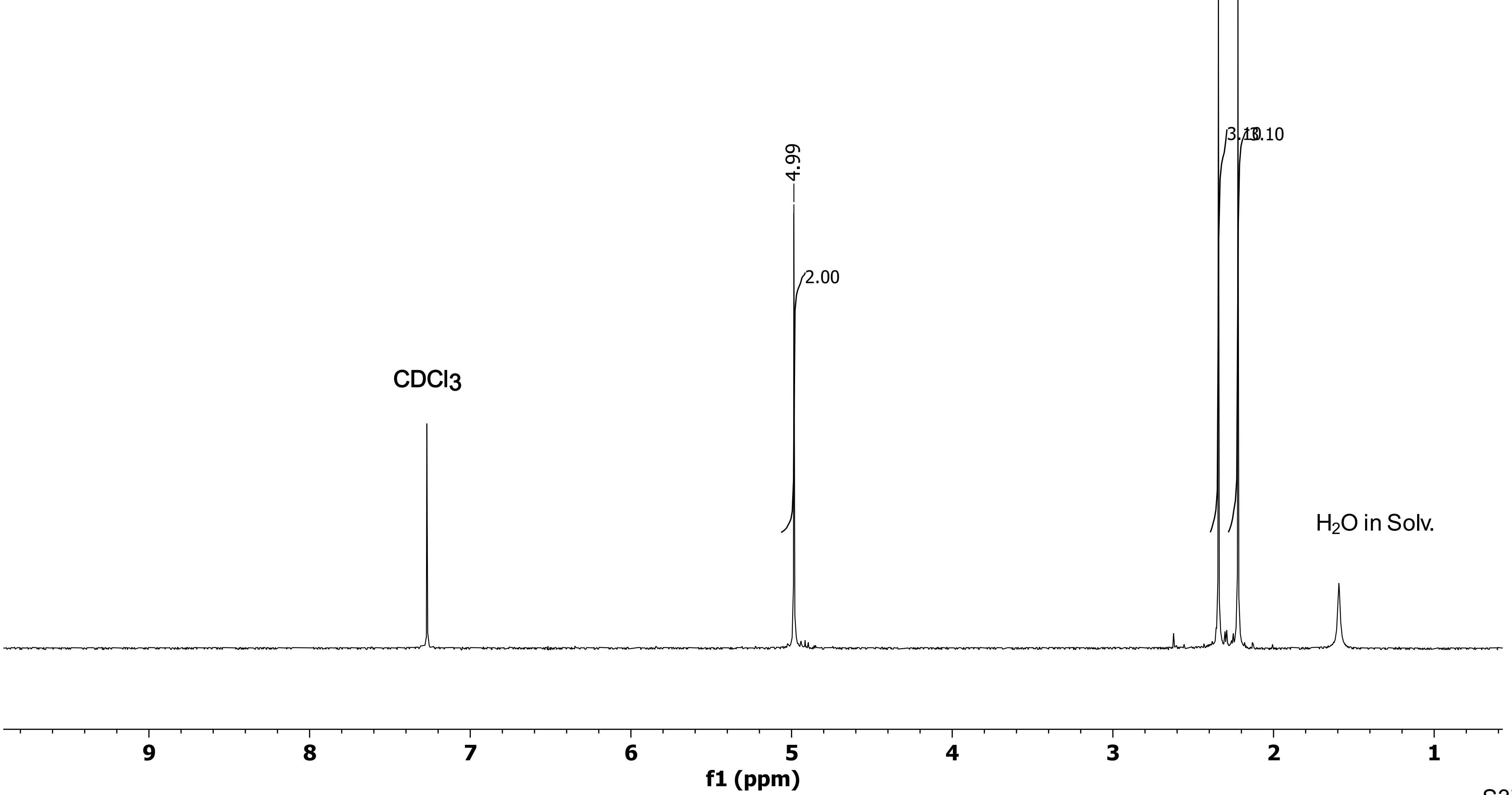

4e

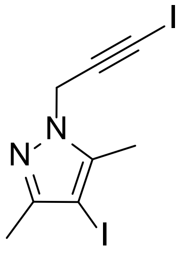

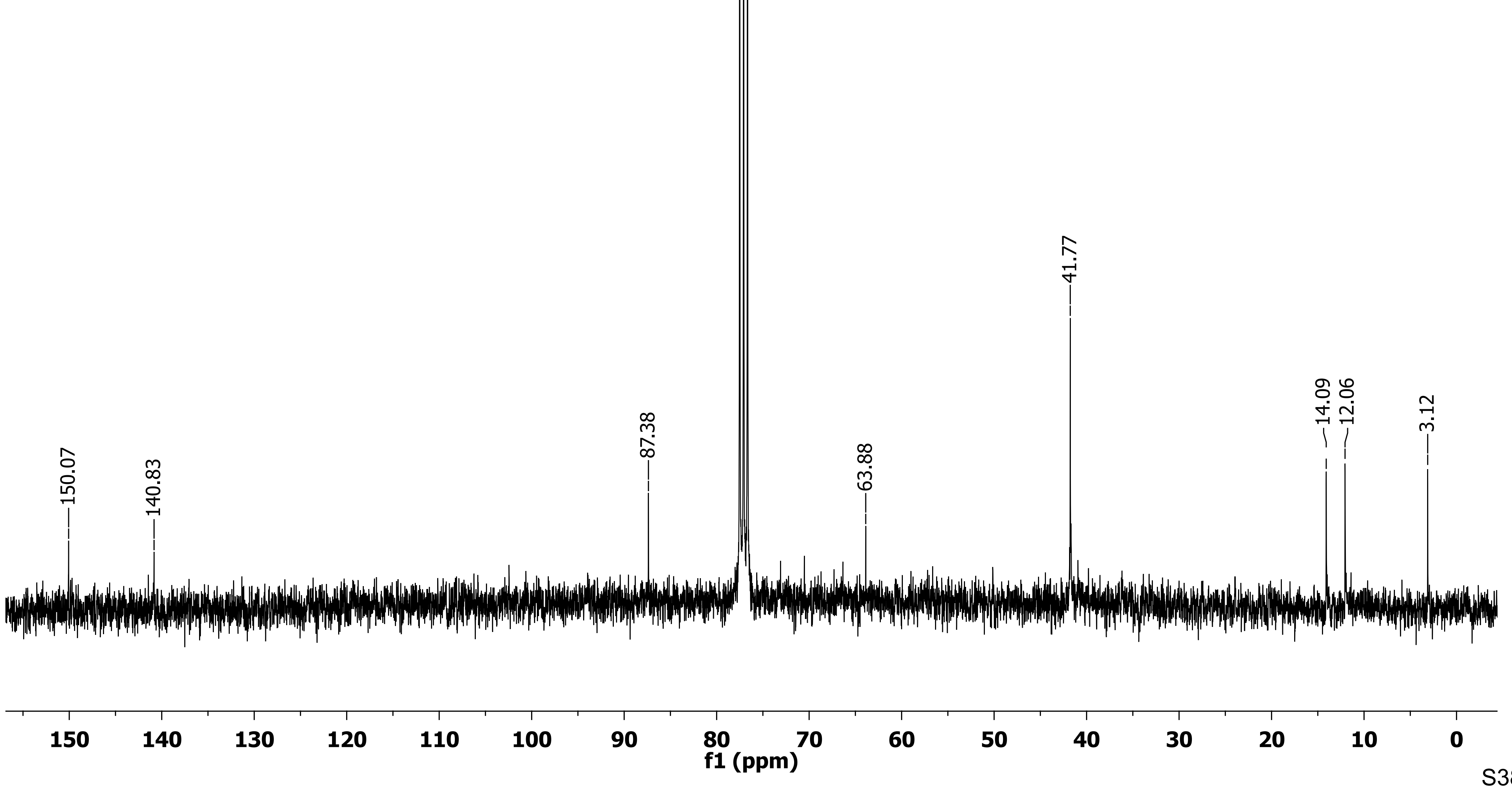
CDCl3
150.07
140.83
87.38
63.88
41.77
14.09
12.06
3.12
150
140
130
120
110
100
90
80
70
60
50
40
30
20
10
0
f1 (ppm)

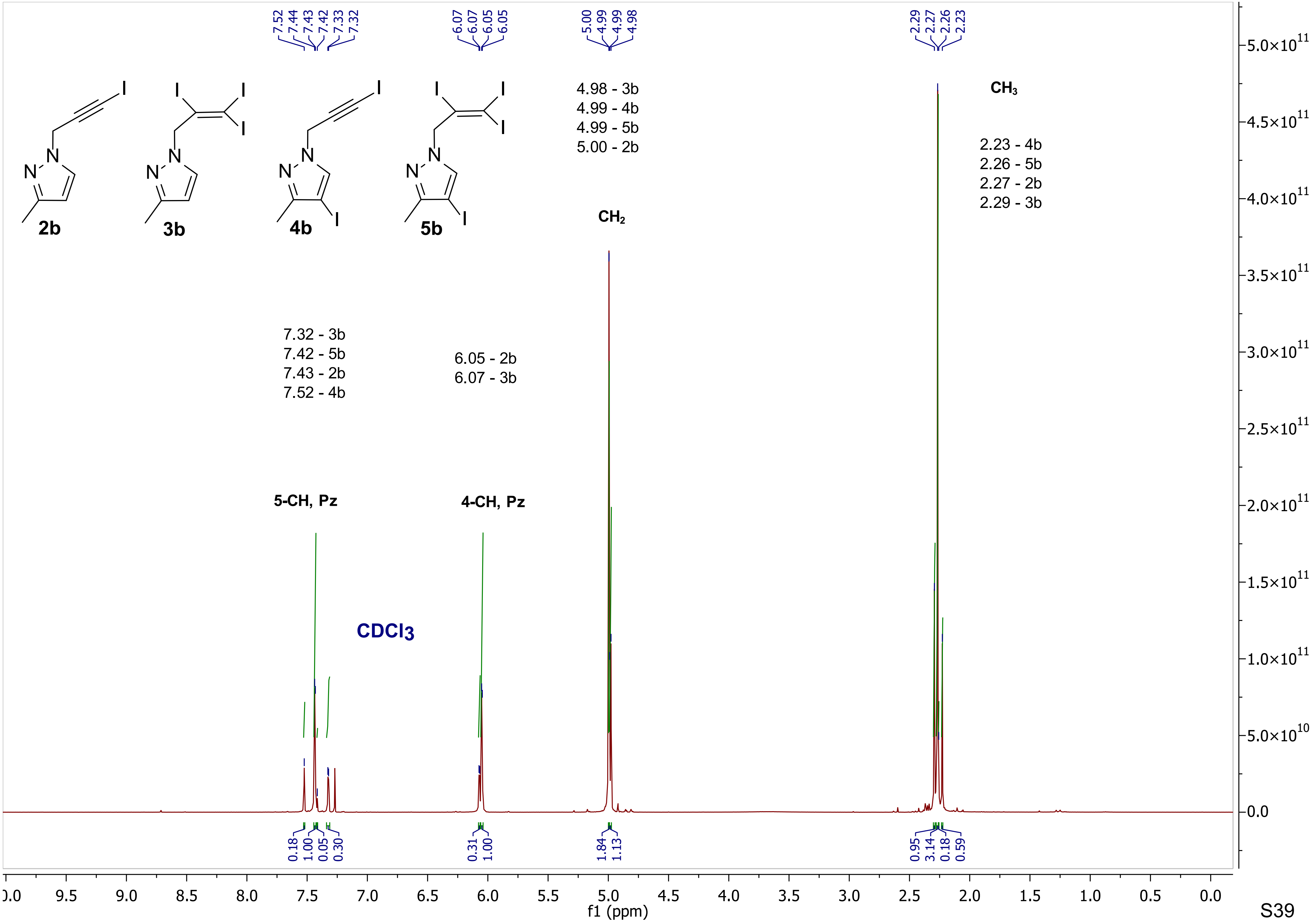

7.52
7.44
7.43
7.42
7.33
7.32
6.07
6.07
6.05
6.05
5.00
4.99
4.99
4.98
2.29
2.27
2.26
2.23
2b
3b
4b
5b
4.98 - 3b
4.99 - 4b
4.99 - 5b
5.00 - 2b
CH₂
CH₃
2.23 - 4b
2.26 - 5b
2.27 - 2b
2.29 - 3b
7.32 - 3b
7.42 - 5b
7.43 - 2b
7.52 - 4b
6.05 - 2b
6.07 - 3b
5-CH, Pz
4-CH, Pz
CDCl3
0.18
1.00
0.05
0.30
0.31
1.00
1.84
1.13
0.95
3.14
0.18
0.59
f1 (ppm)

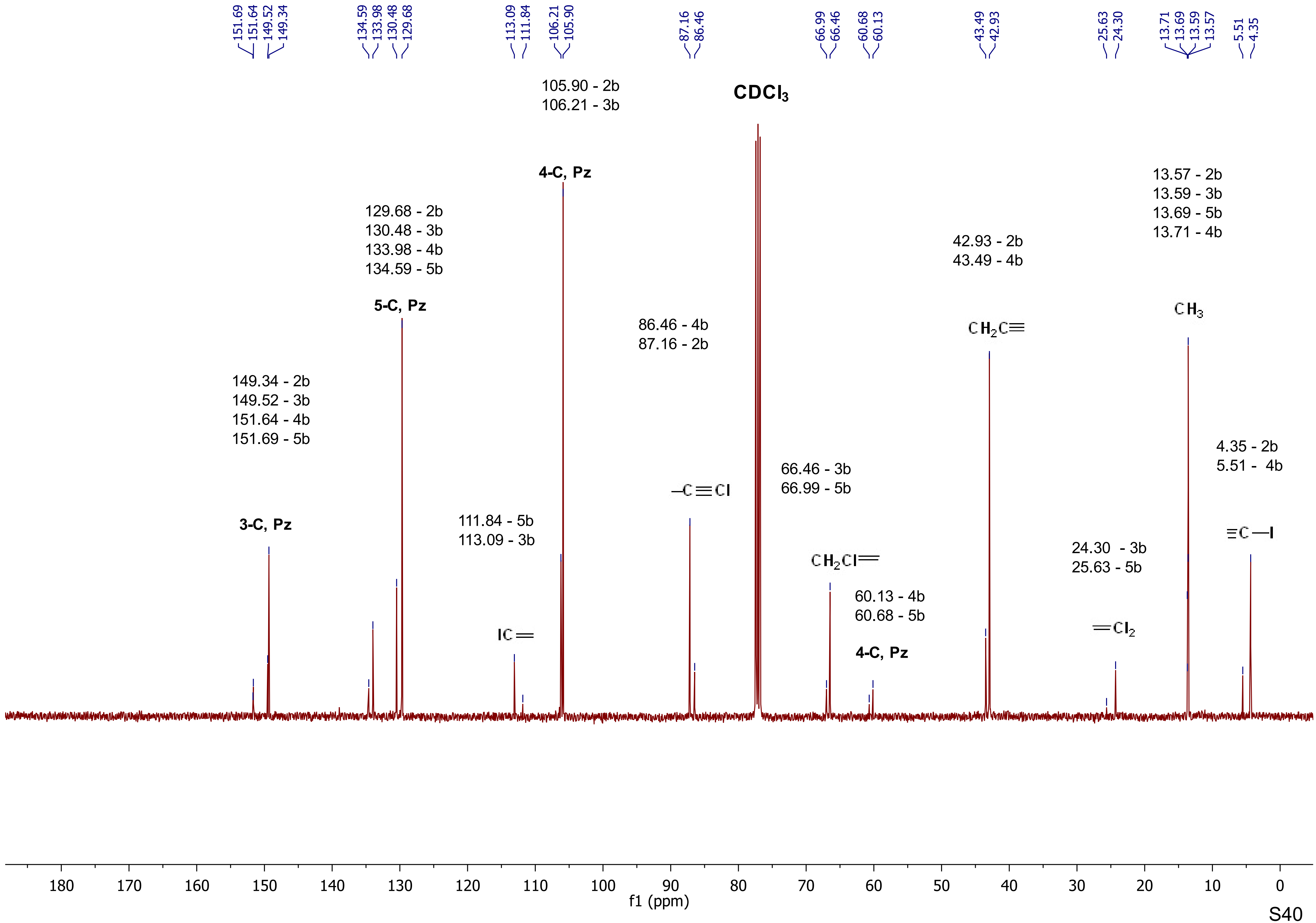
151.69
151.64
149.52
149.34
134.59
133.98
130.48
129.68
113.09
111.84
106.21
105.90
87.16
86.46
66.99
66.46
60.68
60.13
43.49
42.93
25.63
24.30
13.71
13.69
13.59
13.57
5.51
4.35
105.90 - 2b
106.21 - 3b
CDCl3
4-C, Pz
129.68 - 2b
130.48 - 3b
133.98 - 4b
134.59 - 5b
5-C, Pz
149.34 - 2b
149.52 - 3b
151.64 - 4b
151.69 - 5b
3-C, Pz
86.46 - 4b
87.16 - 2b
–C≡CI
111.84 - 5b
113.09 - 3b
IC═
66.46 - 3b
66.99 - 5b
CH2CI═
60.13 - 4b
60.68 - 5b
4-C, Pz
42.93 - 2b
43.49 - 4b
CH2C≡
13.57 - 2b
13.59 - 3b
13.69 - 5b
13.71 - 4b
CH3
24.30 - 3b
25.63 - 5b
═CI2
4.35 - 2b
5.51 - 4b
≡C—I
180 170 160 150 140 130 120 110 100 90 80 70 60 50 40 30 20 10 0
f1 (ppm)

2a

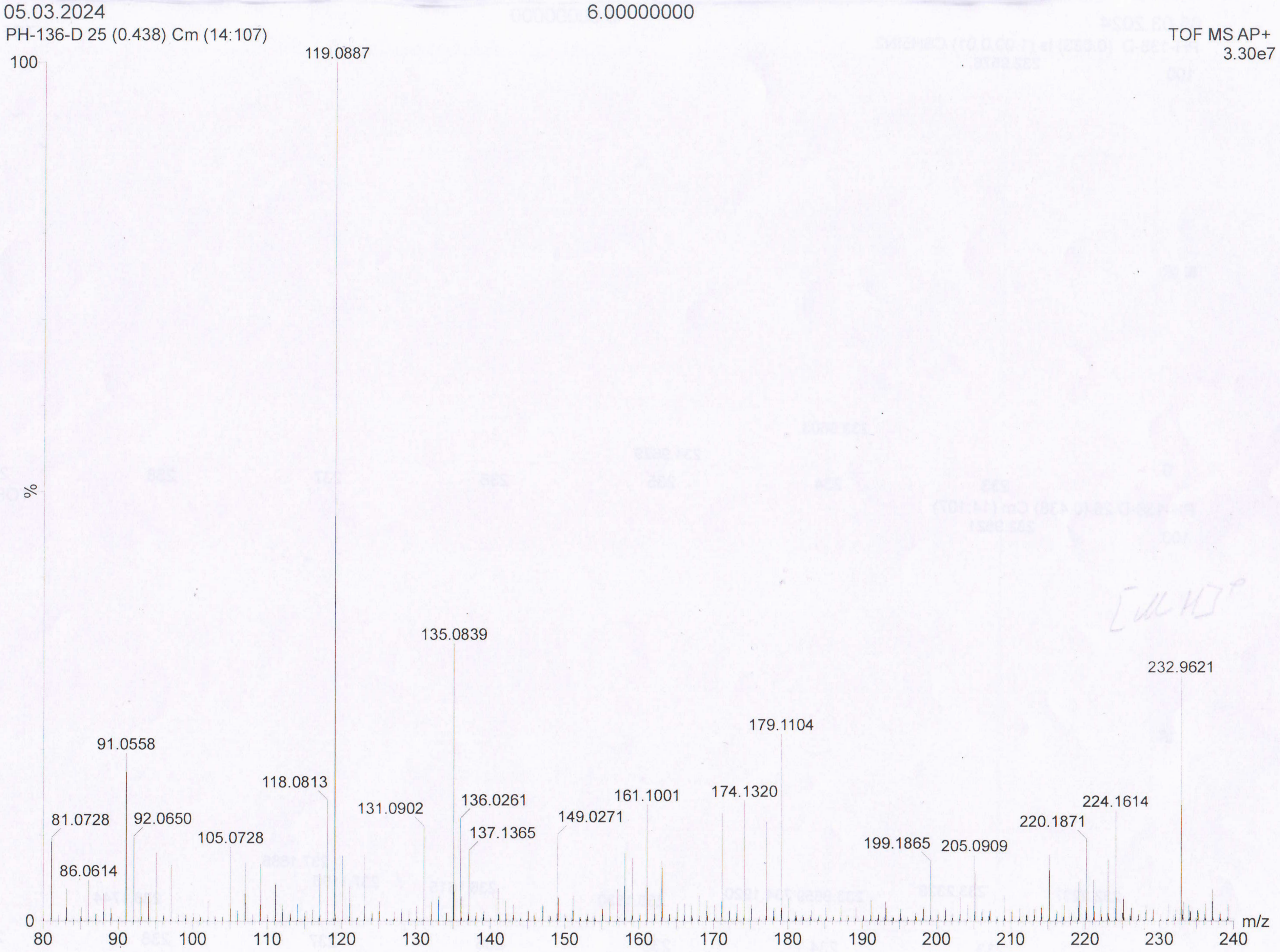
05.03.2024
6.00000000
PH-136-D 25 (0.438) Cm (14:107)
TOF MS AP+
3.30e7
100
%
0
119.0887
135.0839
232.9621
179.1104
91.0558
118.0813
174.1320
161.1001
224.1614
136.0261
131.0902
81.0728
92.0650
149.0271
220.1871
137.1365
105.0728
199.1865
205.0909
86.0614
80
90
100
110
120
130
140
150
160
170
180
190
200
210
220
230
240
m/z

2a

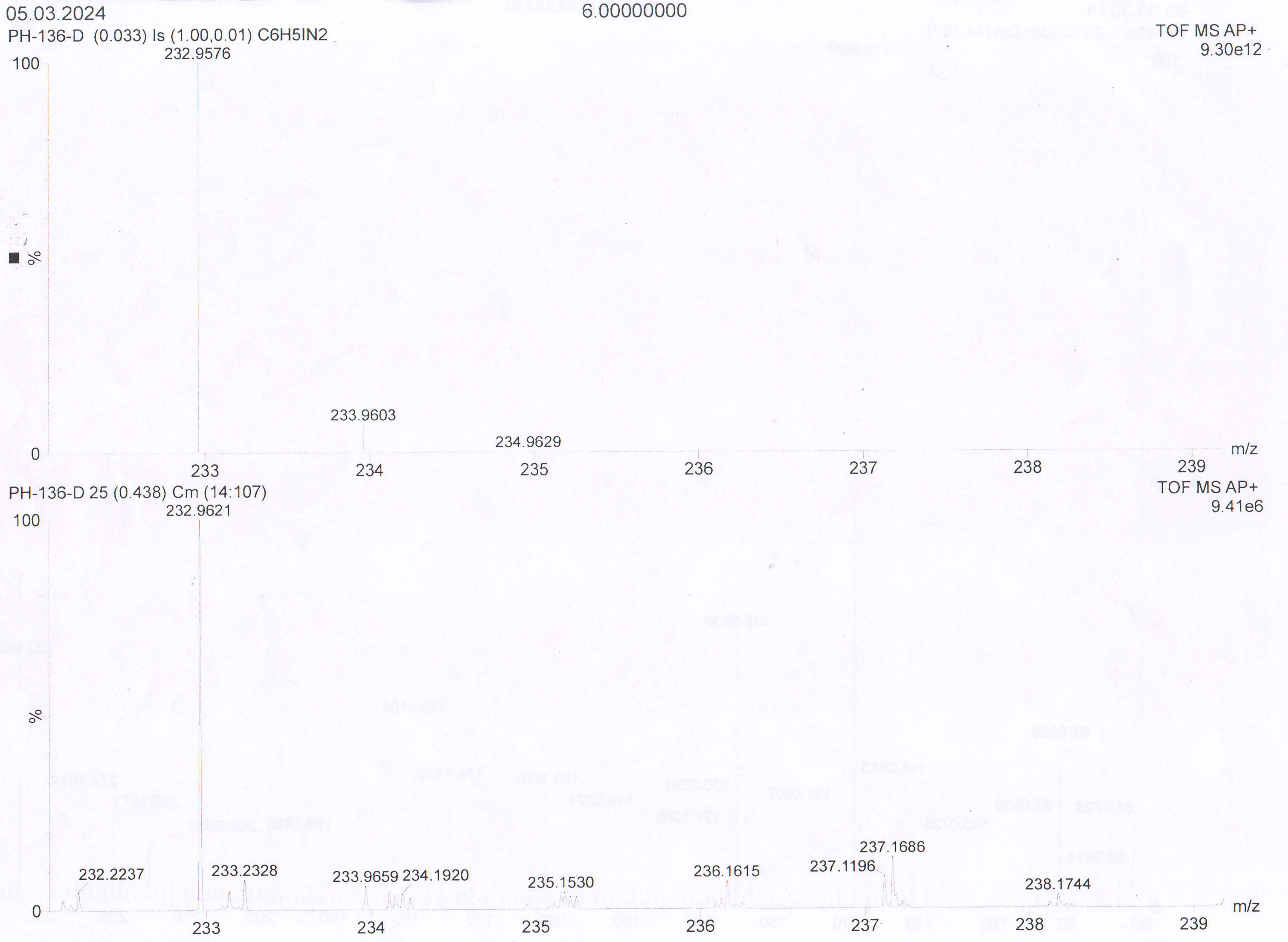
05.03.2024
6.00000000
PH-136-D (0.033) Is (1.00,0.01) C6H5IN2
TOF MS AP+
9.30e12
100
%
0
232.9576
233.9603
234.9629
233
234
235
236
237
238
239
m/z
PH-136-D 25 (0.438) Cm (14:107)
TOF MS AP+
9.41e6
232.9621
232.2237
233.2328
233.9659
234.1920
235.1530
236.1615
237.1196
237.1686
238.1744

2b

22.12.2023

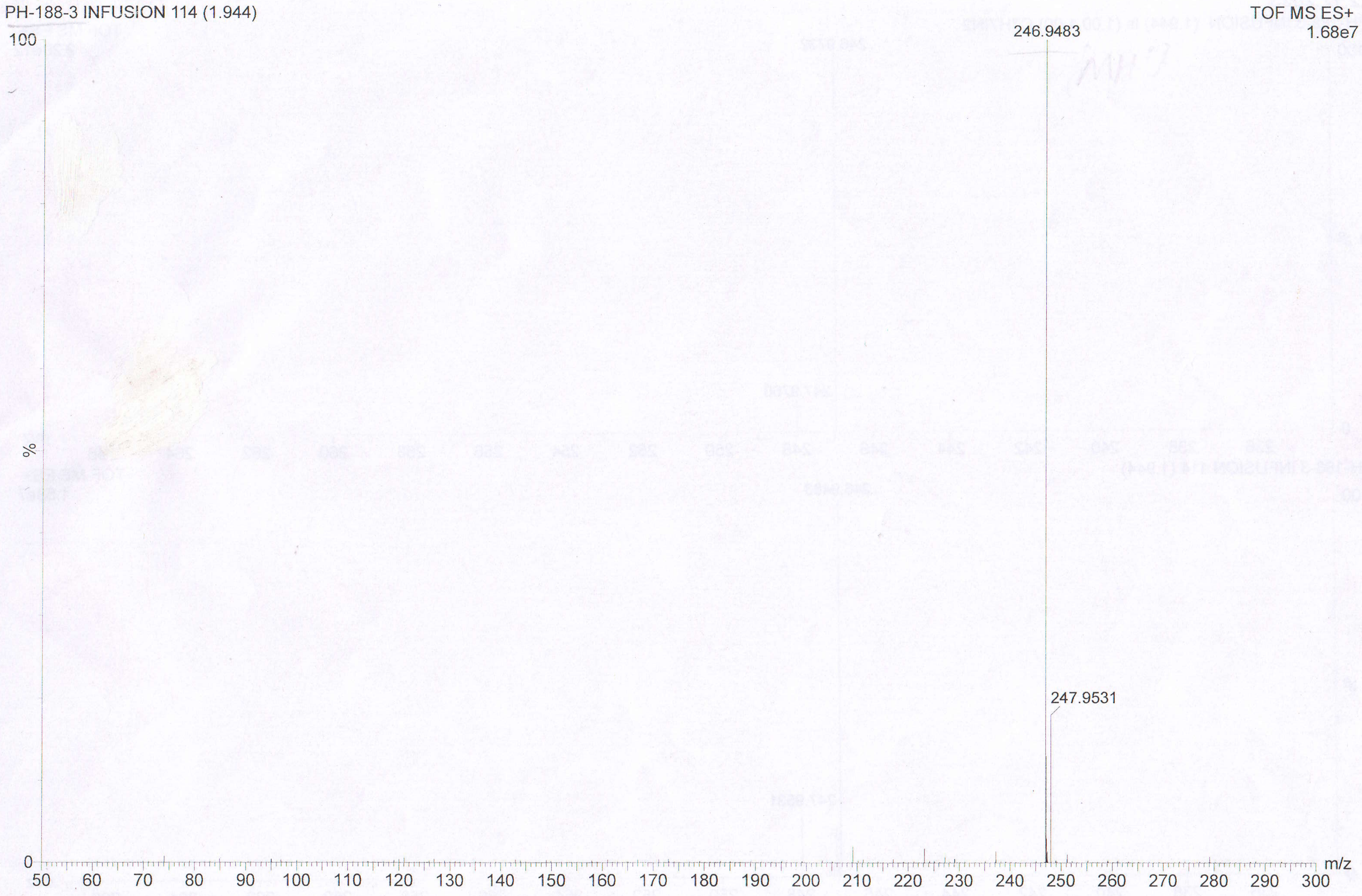
PH-188-3 INFUSION 114 (1.944)
TOF MS ES+
1.68e7
246.9483
247.9531
100
%
0
m/z
50
60
70
80
90
100
110
120
130
140
150
160
170
180
190
200
210
220
230
240
250
260
270
280
290
300

2b

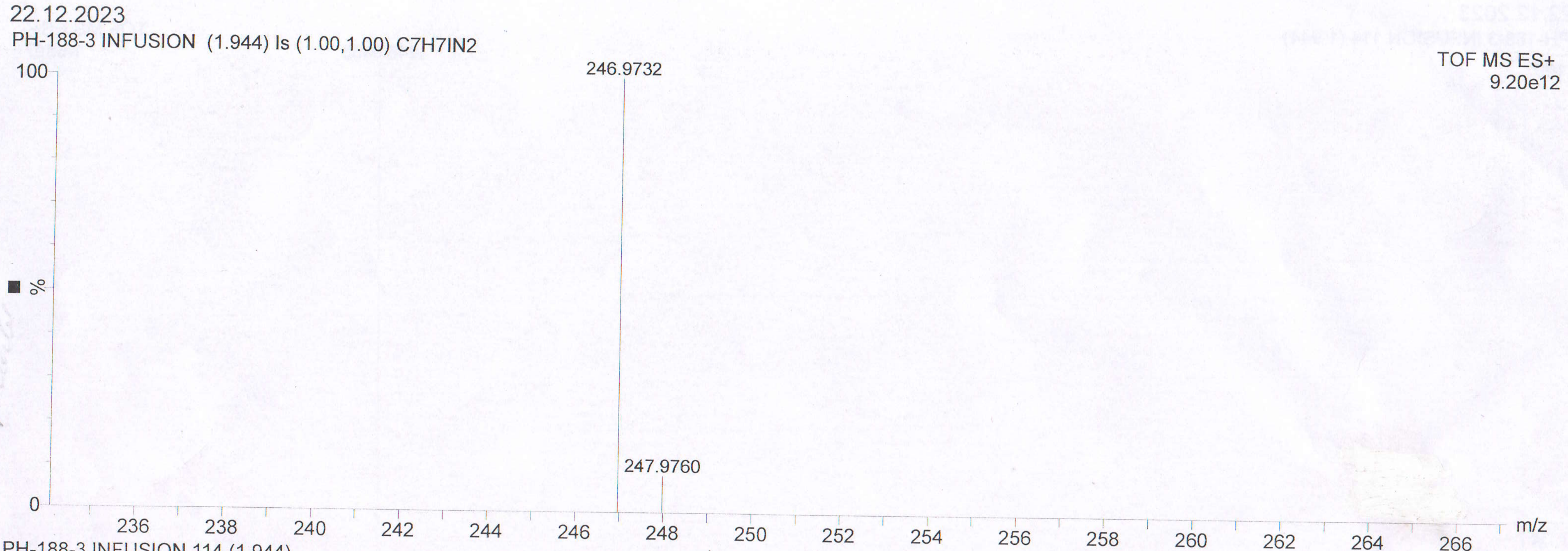
22.12.2023
PH-188-3 INFUSION (1.944) Is (1.00,1.00) C7H7IN2
TOF MS ES+
9.20e12
246.9732
247.9760
100
%
0
236
238
240
242
244
246
248
250
252
254
256
258
260
262
264
266
m/z

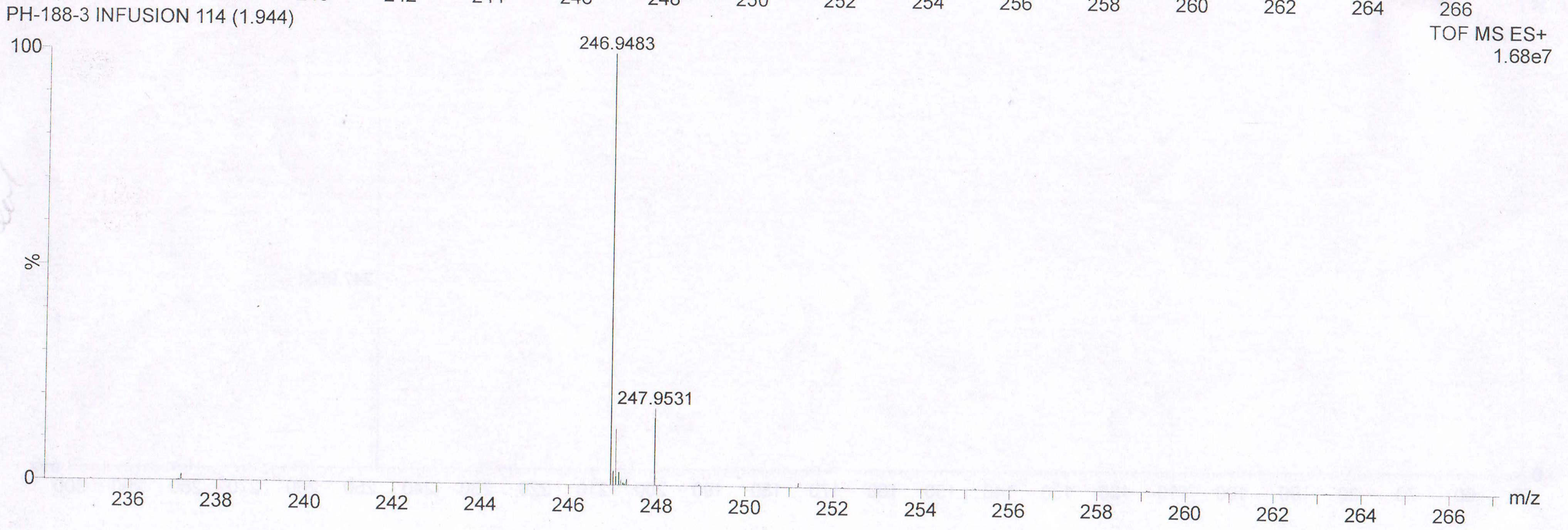
PH-188-3 INFUSION 114 (1.944)
TOF MS ES+
1.68e7
246.9483
247.9531
100
%
0
236
238
240
242
244
246
248
250
252
254
256
258
260
262
264
266
m/z

2c

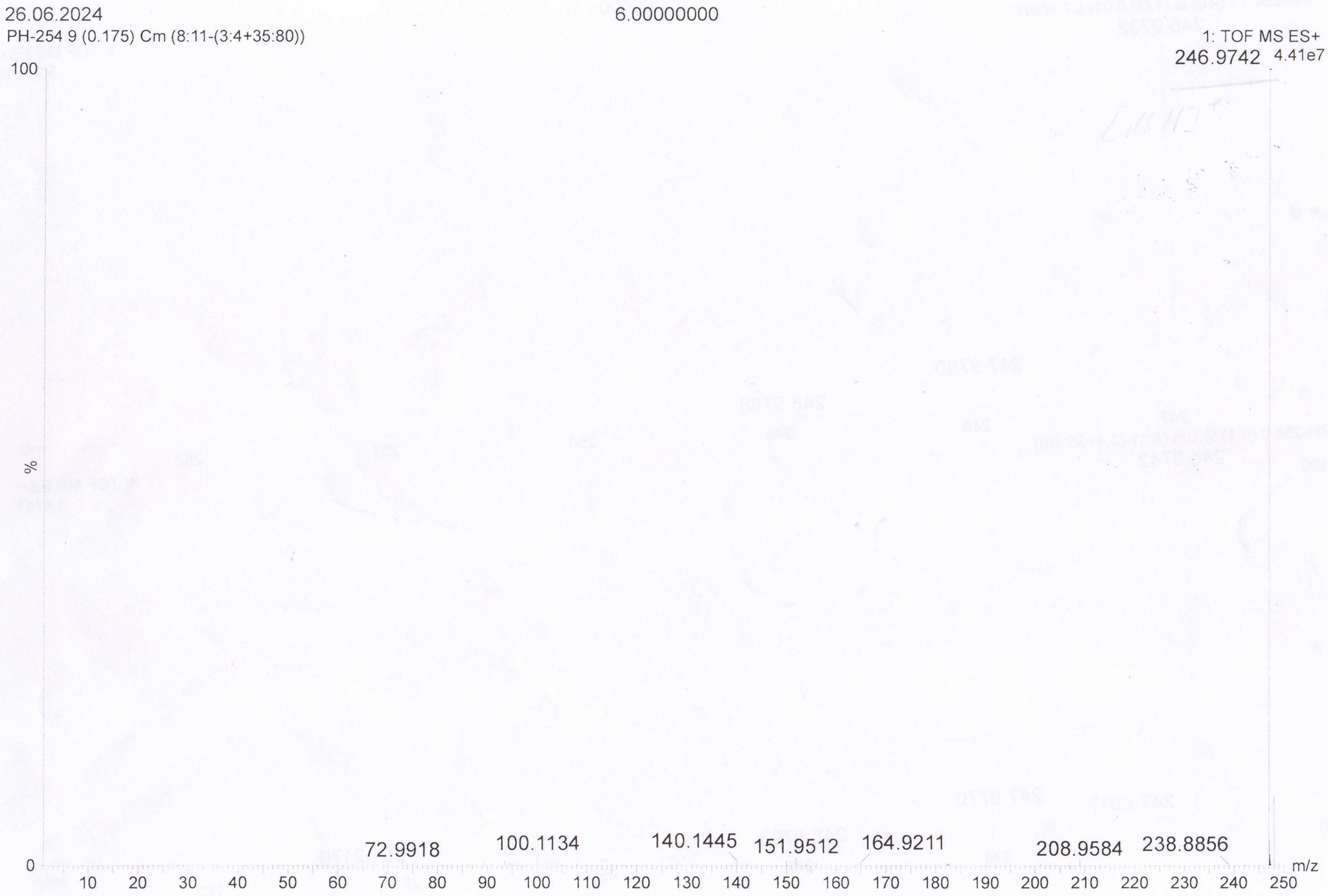
26.06.2024
6.00000000
PH-254 9 (0.175) Cm (8:11-(3:4+35:80))
1: TOF MS ES+
246.9742
4.41e7
100
%
0
72.9918
100.1134
140.1445
151.9512
164.9211
208.9584
238.8856
m/z
10 20 30 40 50 60 70 80 90 100 110 120 130 140 150 160 170 180 190 200 210 220 230 240 250

2c

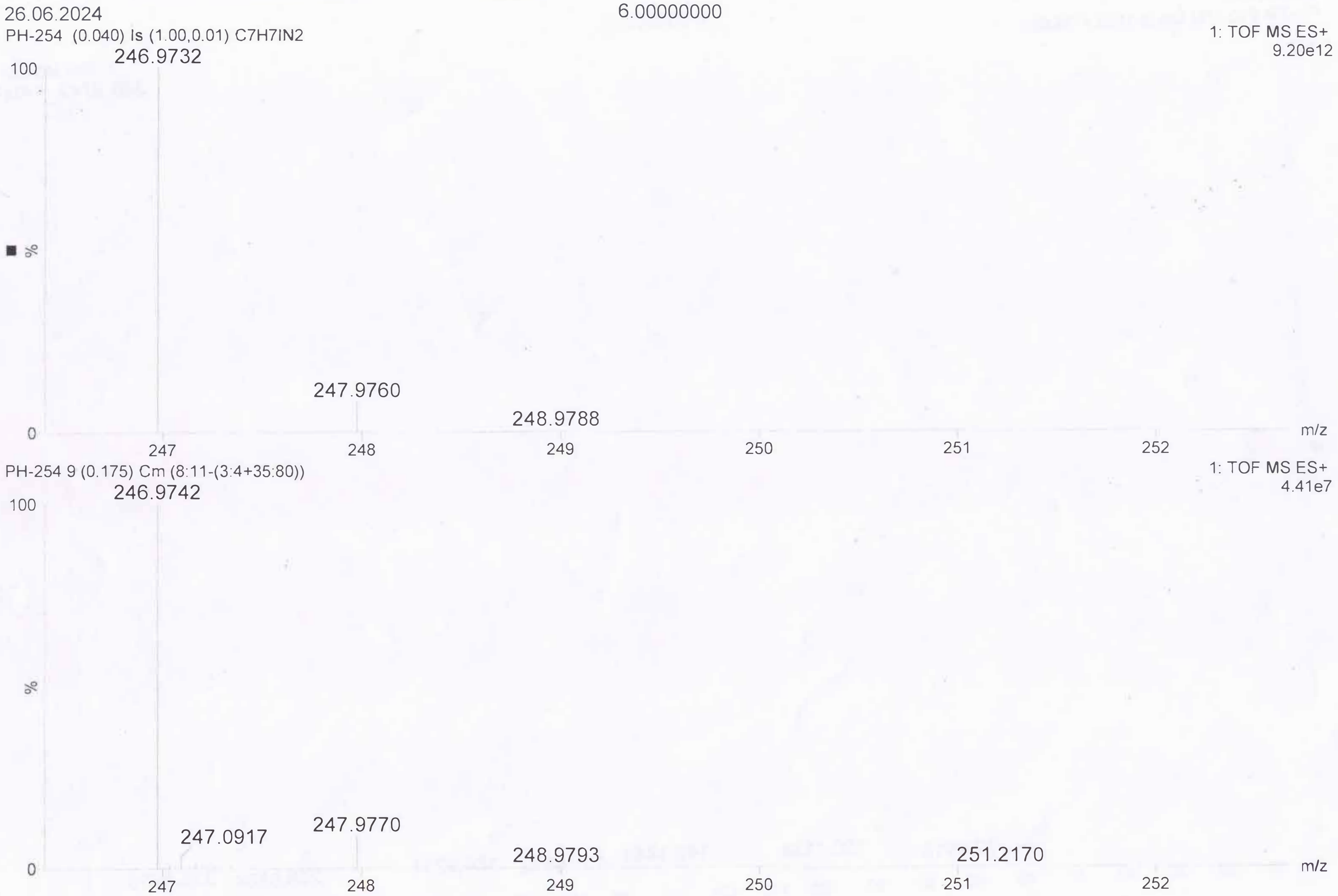
26.06.2024
6.00000000
PH-254 (0.040) Is (1.00,0.01) C7H7IN2
1: TOF MS ES+
9.20e12
246.9732
100
%
247.9760
248.9788
0
m/z
247
248
249
250
251
252
PH-254 9 (0.175) Cm (8:11-(3:4+35:80))
1: TOF MS ES+
4.41e7
246.9742
100
%
247.0917
247.9770
248.9793
251.2170
0
m/z
247
248
249
250
251
252

3a

25.12.2023

PH-137D 92 (1.571) AM (Cen,4, 80.00, Ar,10000.0,0.00,0.00); Cm (43:116)

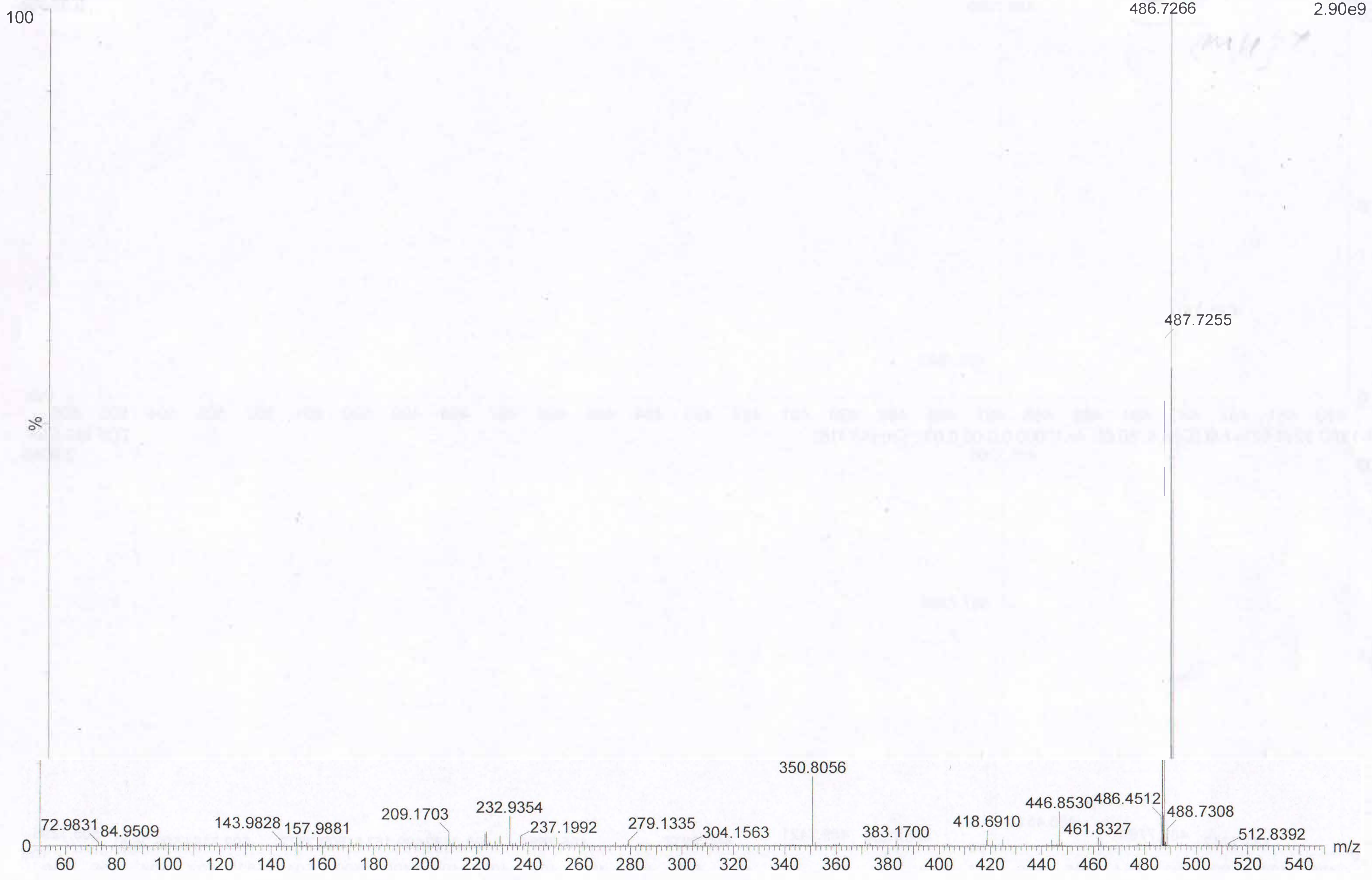

3a

25.12.2023

PH-137D (0.033) Is (1.00,1.00) C6H5I3N2

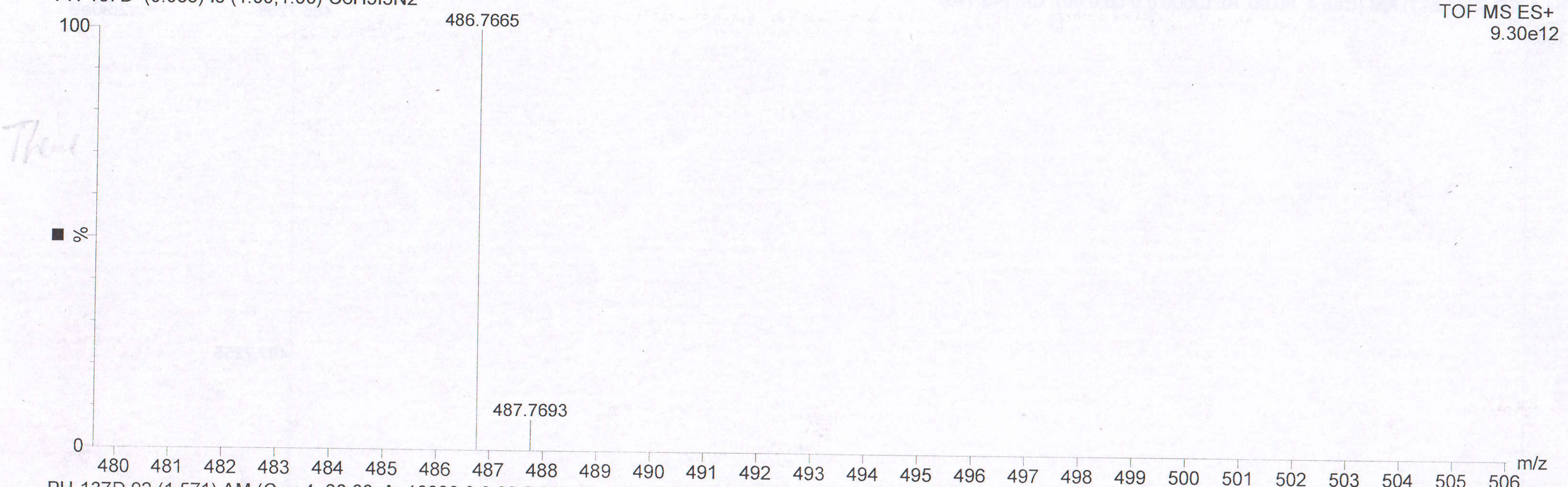

PH-137D 92 (1.571) AM (Cen,4, 80.00, Ar,10000.0,0.00,0.00); Cm (43:116)

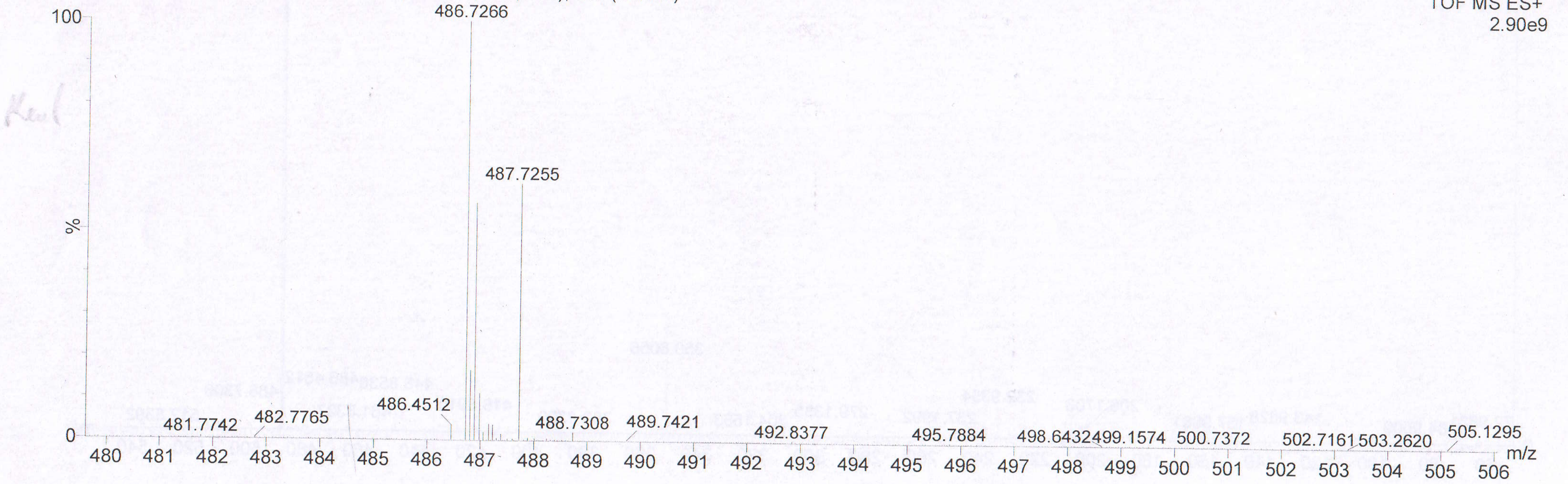

4e

20.12.2023

PH-159 n 27 (0.480) AM (Cen,4, 80.00, Ar,10000.0,0.00,0.00); Cm (27:49)

1: TOF MS ES+
386.8839 1.44e8

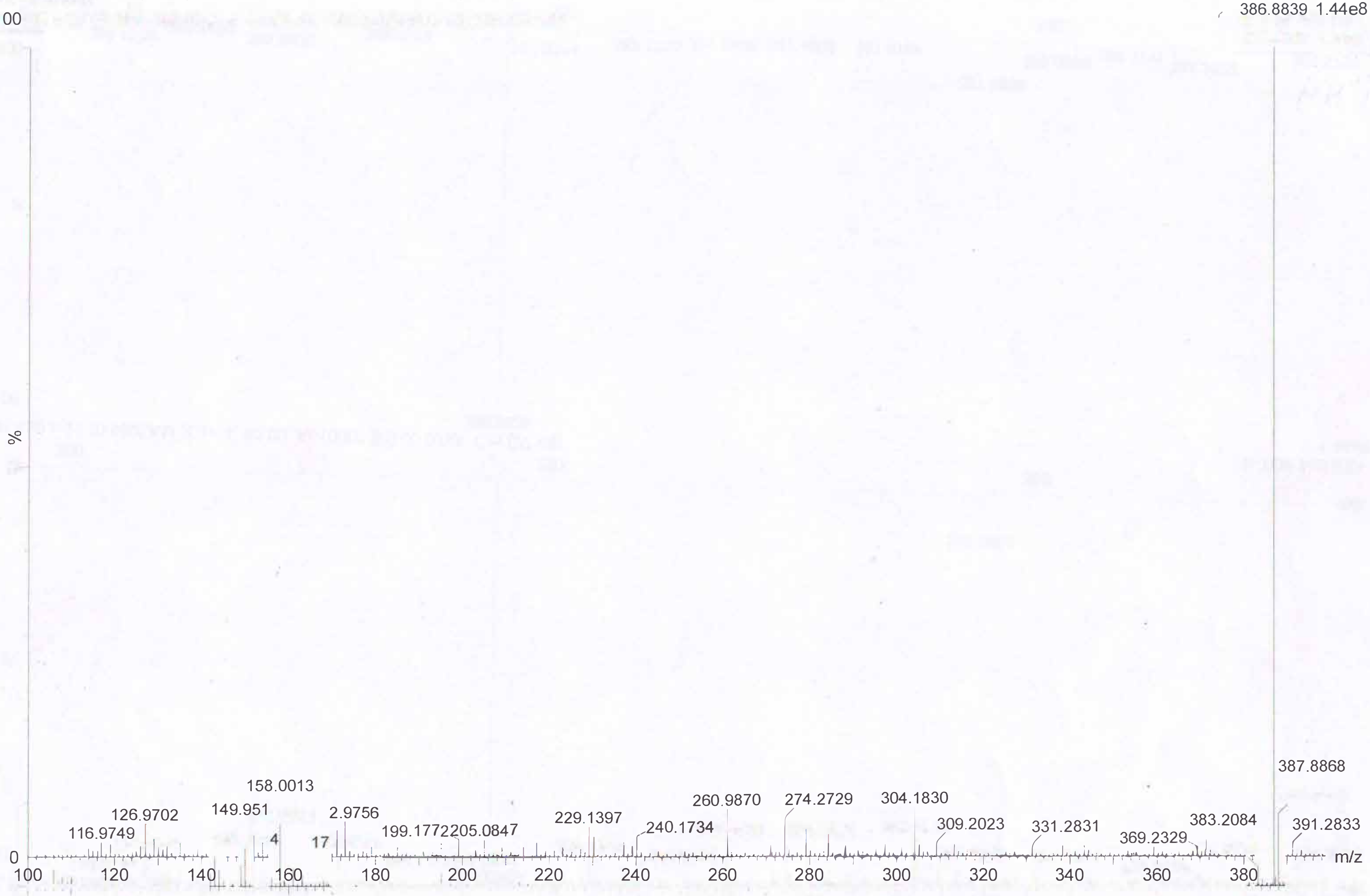

4e

20.12.2023

PH-159 n (0.040) Is (1.00,1.00) C8H8I2N2

1: TOF MS ES+ 9.10e12

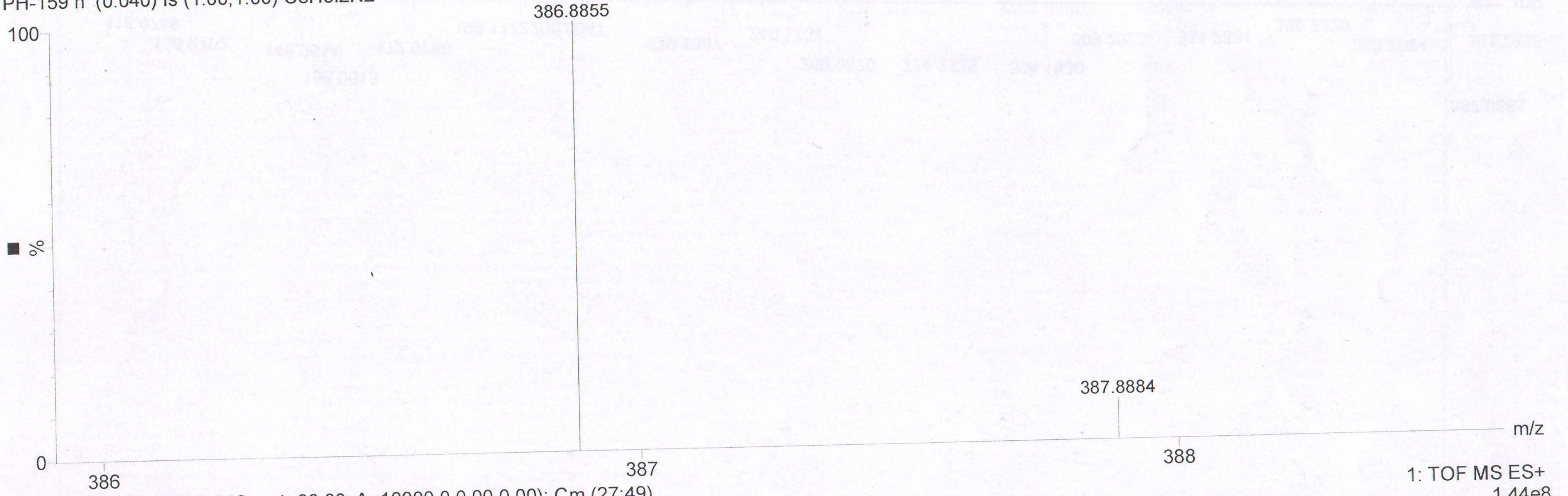

PH-159 n 27 (0.480) AM (Cen,4, 80.00, Ar,10000.0,0.00,0.00); Cm (27:49)

1: TOF MS ES+ 1.44e8

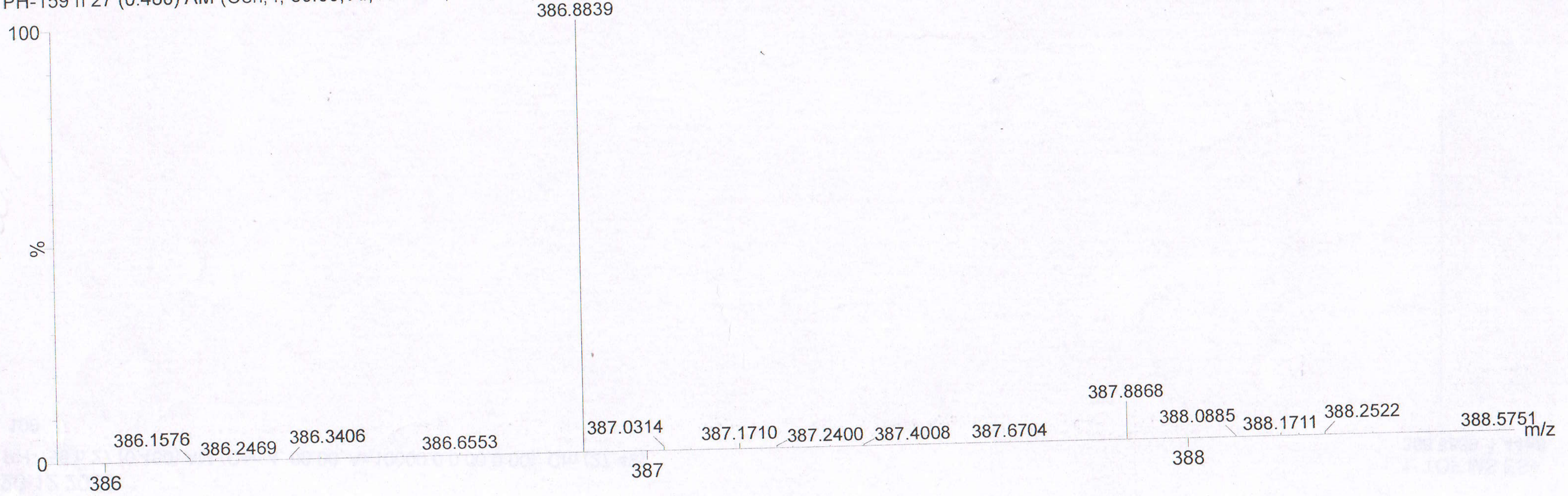

7a

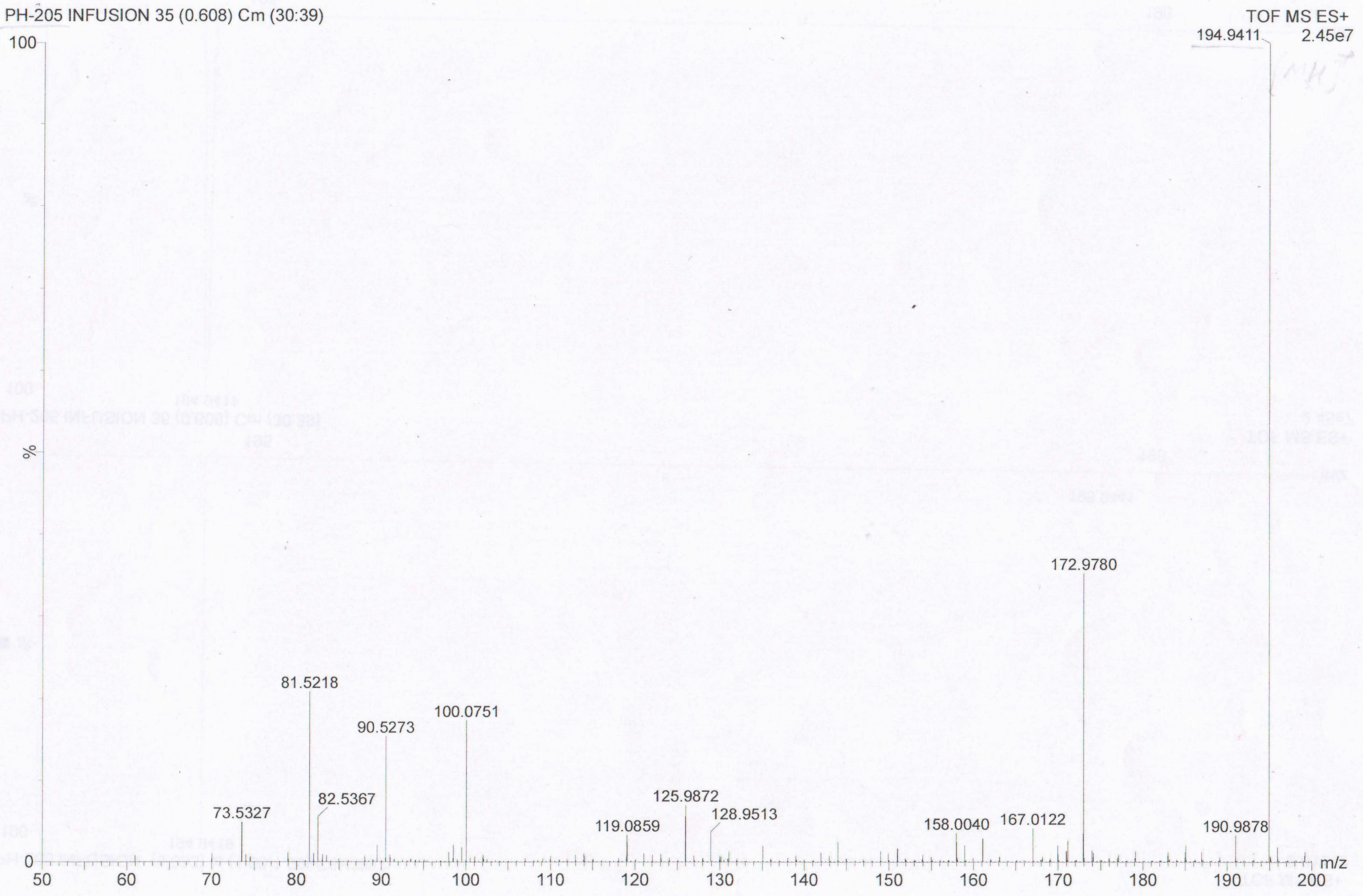

PH-205 INFUSION 35 (0.608) Cm (30:39)
TOF MS ES+
2.45e7
194.9411
172.9780
81.5218
100.0751
90.5273
82.5367
73.5327
125.9872
128.9513
119.0859
158.0040
167.0122
190.9878
100
%
0
50
60
70
80
90
100
110
120
130
140
150
160
170
180
190
200
m/z

7a

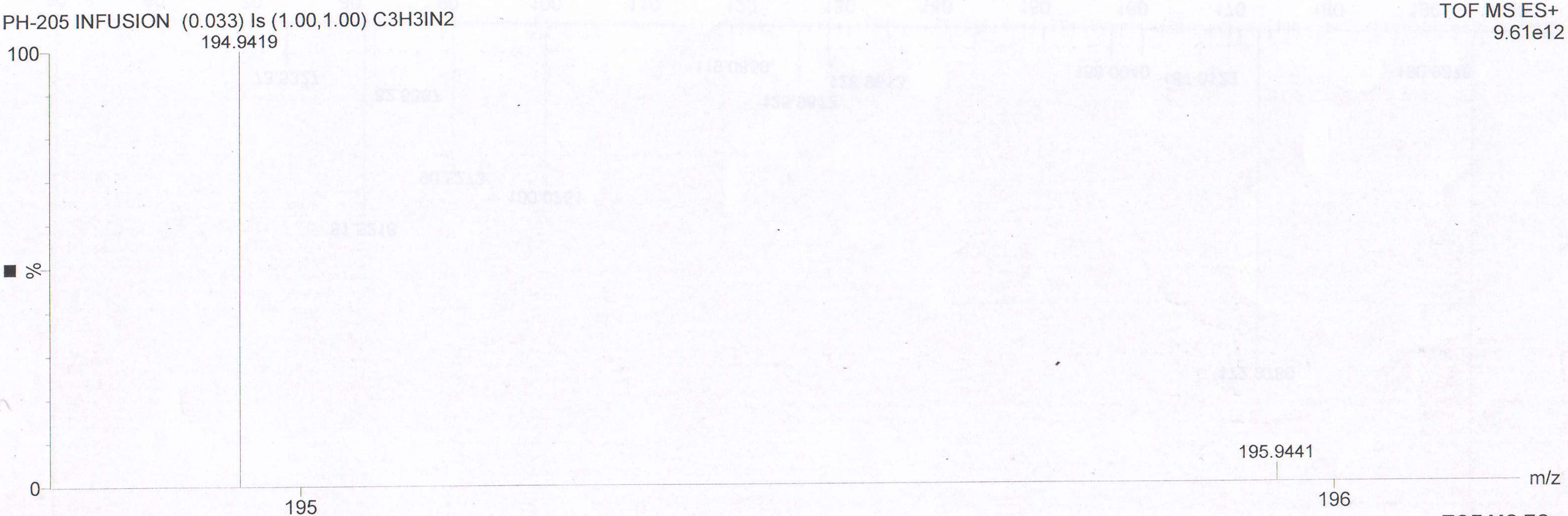
PH-205 INFUSION (0.033) Is (1.00,1.00) C3H3IN2
TOF MS ES+
9.61e12
194.9419
195.9441
100
%
0
195
196
m/z

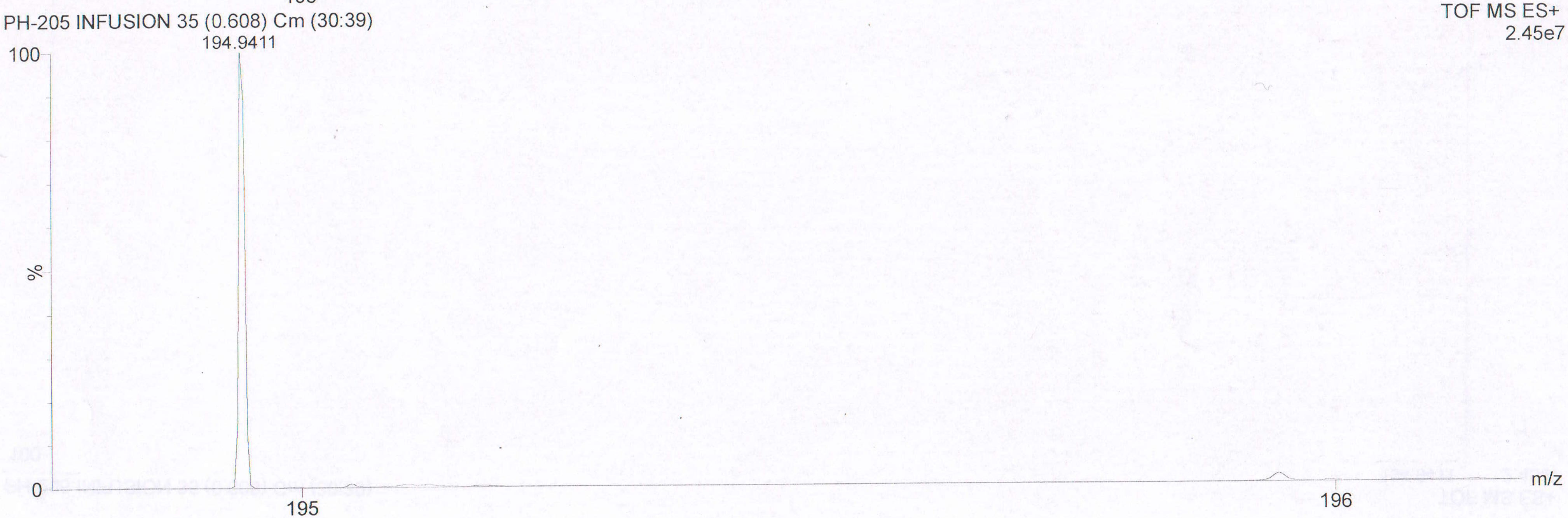
PH-205 INFUSION 35 (0.608) Cm (30:39)
TOF MS ES+
2.45e7
194.9411
100
%
0
195
196
m/z

7b

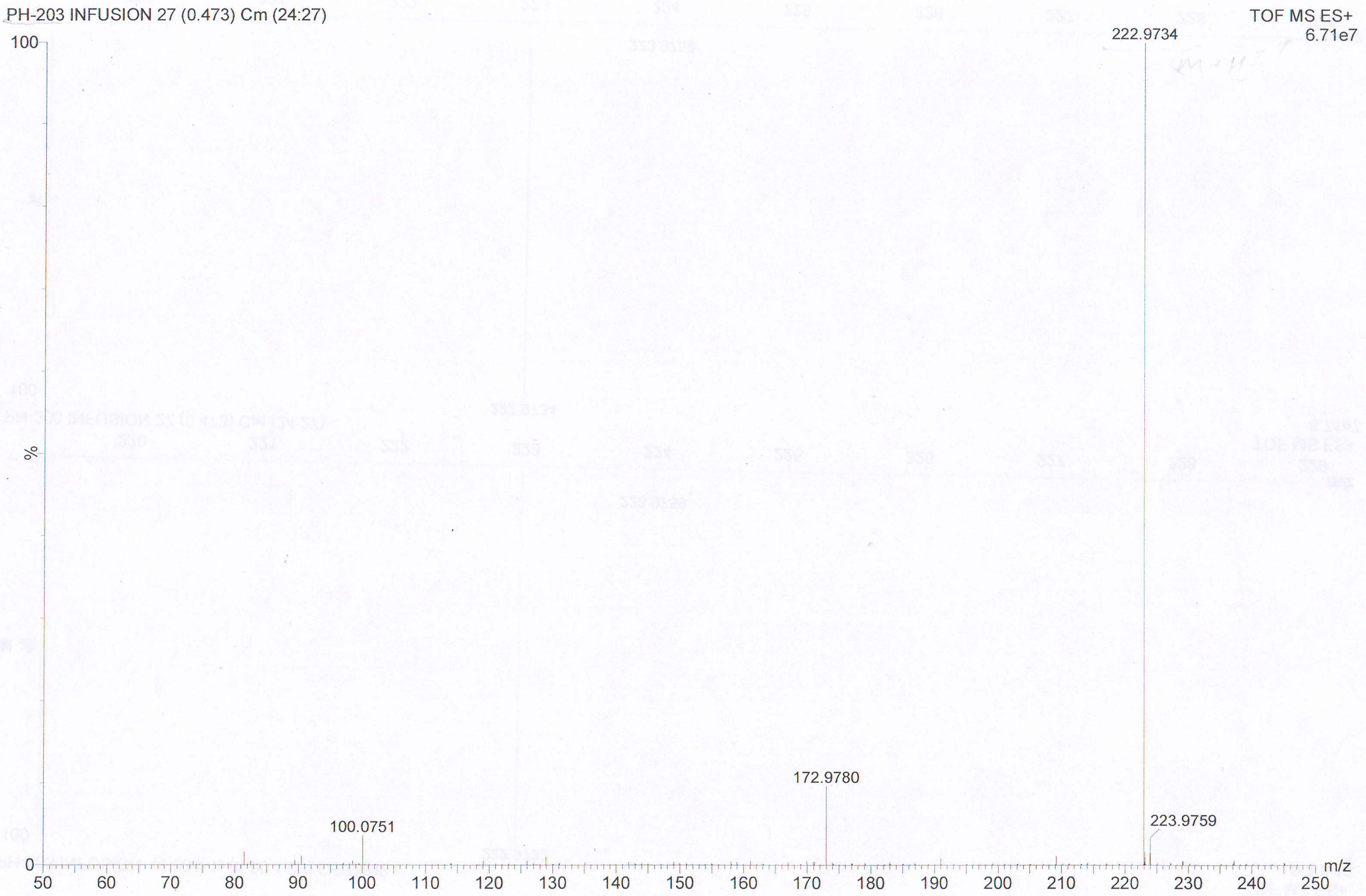
PH-203 INFUSION 27 (0.473) Cm (24:27)
TOF MS ES+
6.71e7
222.9734
172.9780
100.0751
223.9759
%
m/z

7b

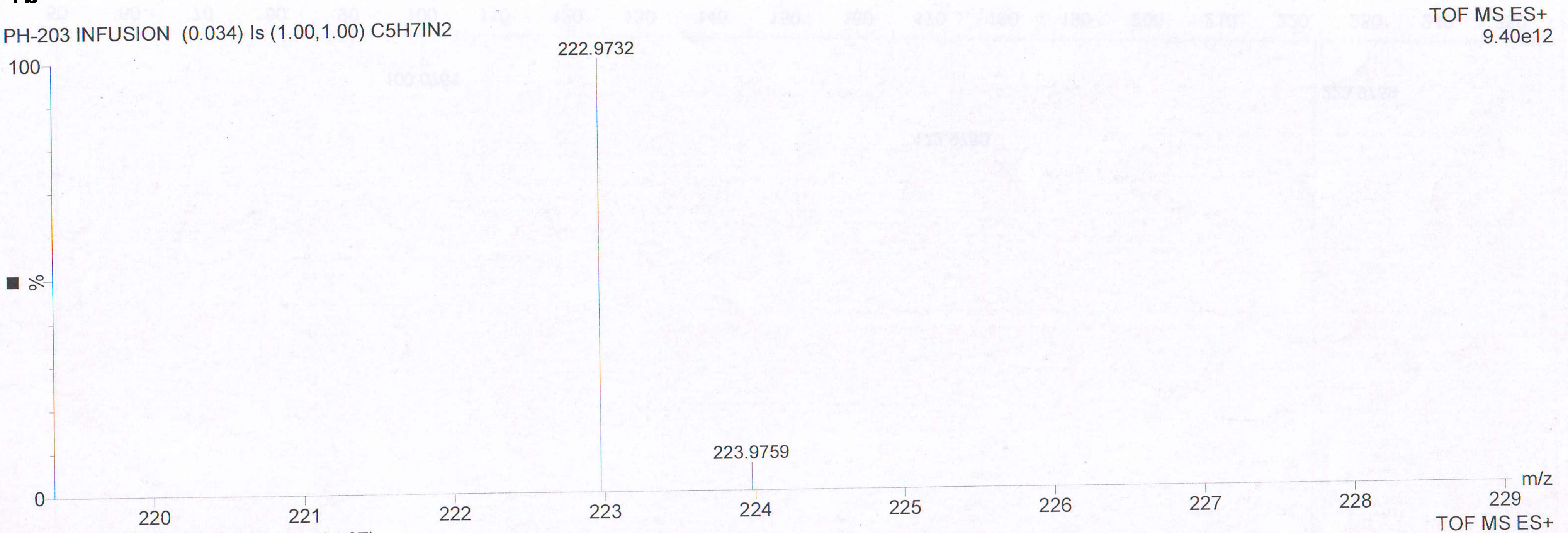

PH-203 INFUSION (0.034) Is (1.00,1.00) C5H7IN2
TOF MS ES+
9.40e12
222.9732
223.9759
100
%
0
220
221
222
223
224
225
226
227
228
229
m/z

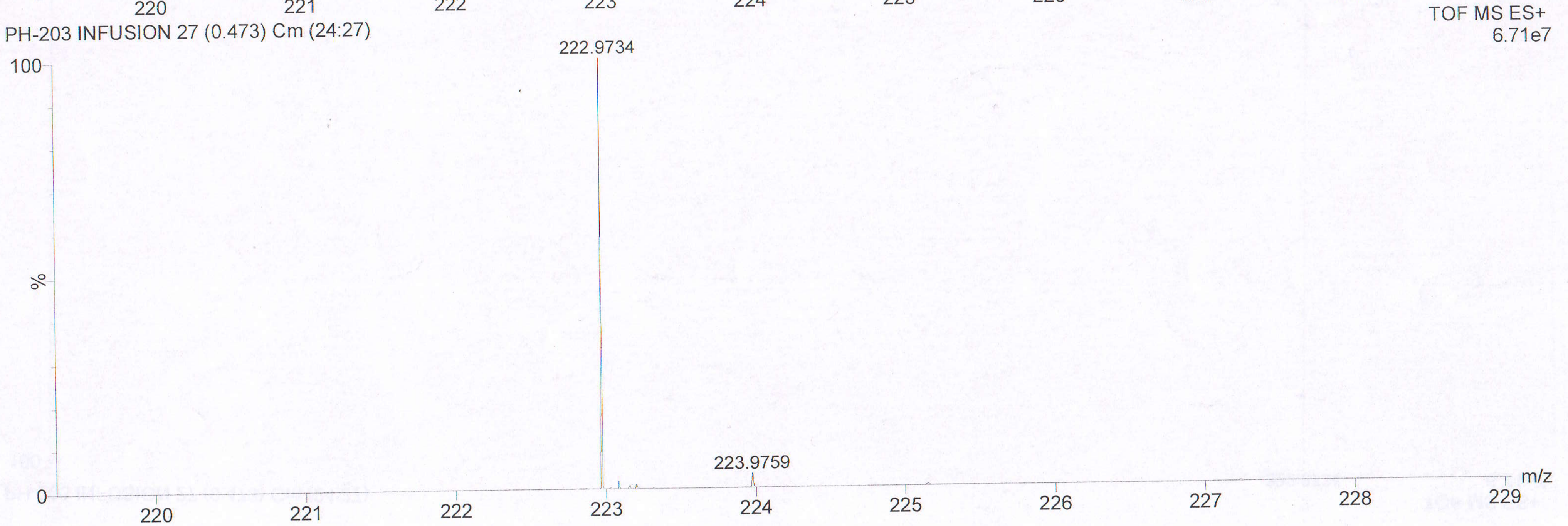

PH-203 INFUSION 27 (0.473) Cm (24:27)
TOF MS ES+
6.71e7
222.9734
223.9759
100
%
0
220
221
222
223
224
225
226
227
228
229
m/z

7c

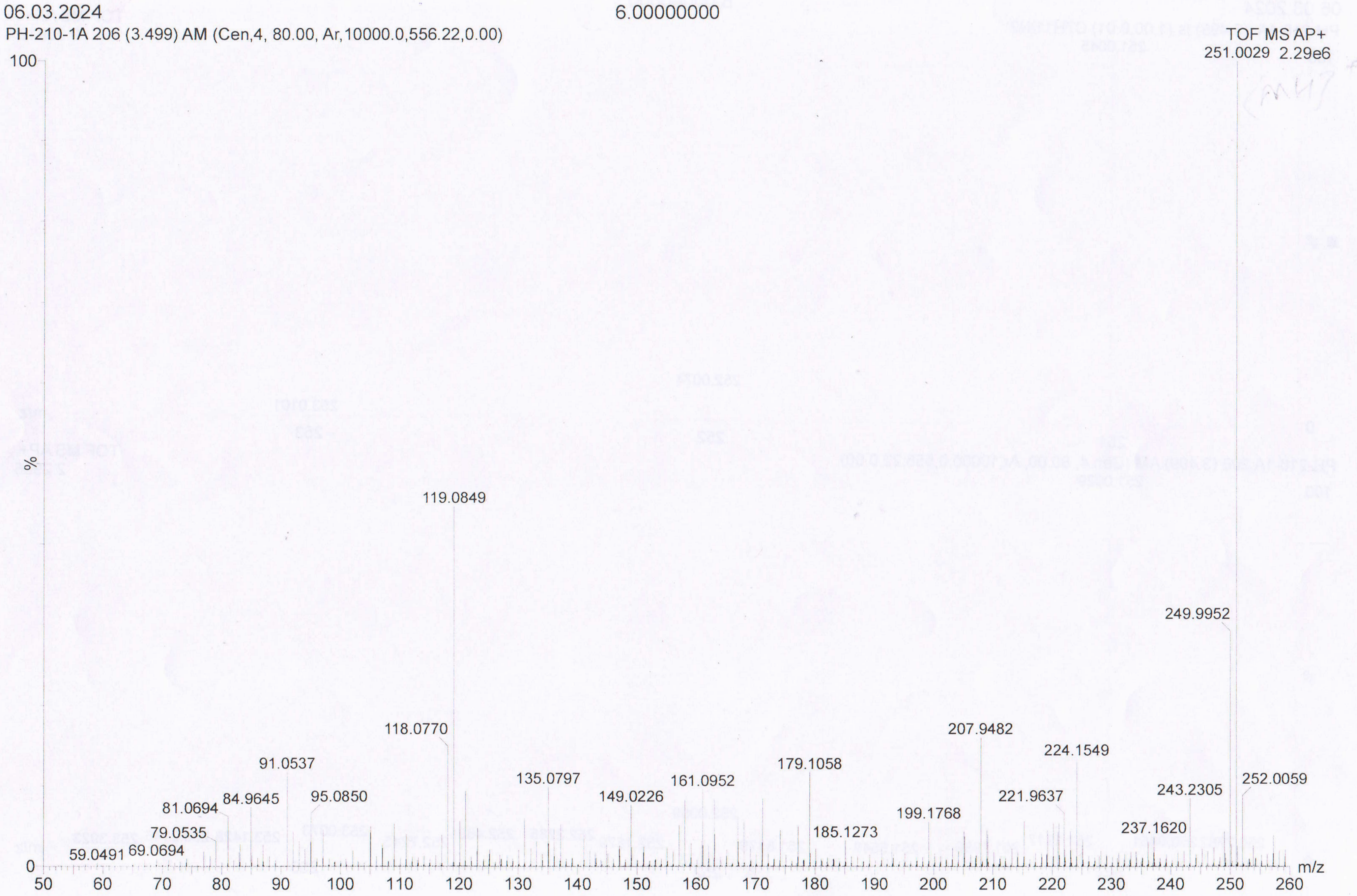

06.03.2024
6.00000000
PH-210-1A 206 (3.499) AM (Cen,4, 80.00, Ar,10000.0,556.22,0.00)
TOF MS AP+
251.0029 2.29e6
(MH)+
100
%
0
119.0849
249.9952
118.0770
207.9482
224.1549
91.0537
179.1058
135.0797
161.0952
252.0059
84.9645
95.0850
149.0226
221.9637
243.2305
81.0694
199.1768
79.0535
185.1273
237.1620
59.0491
69.0694
50
60
70
80
90
100
110
120
130
140
150
160
170
180
190
200
210
220
230
240
250
260
m/z

7c

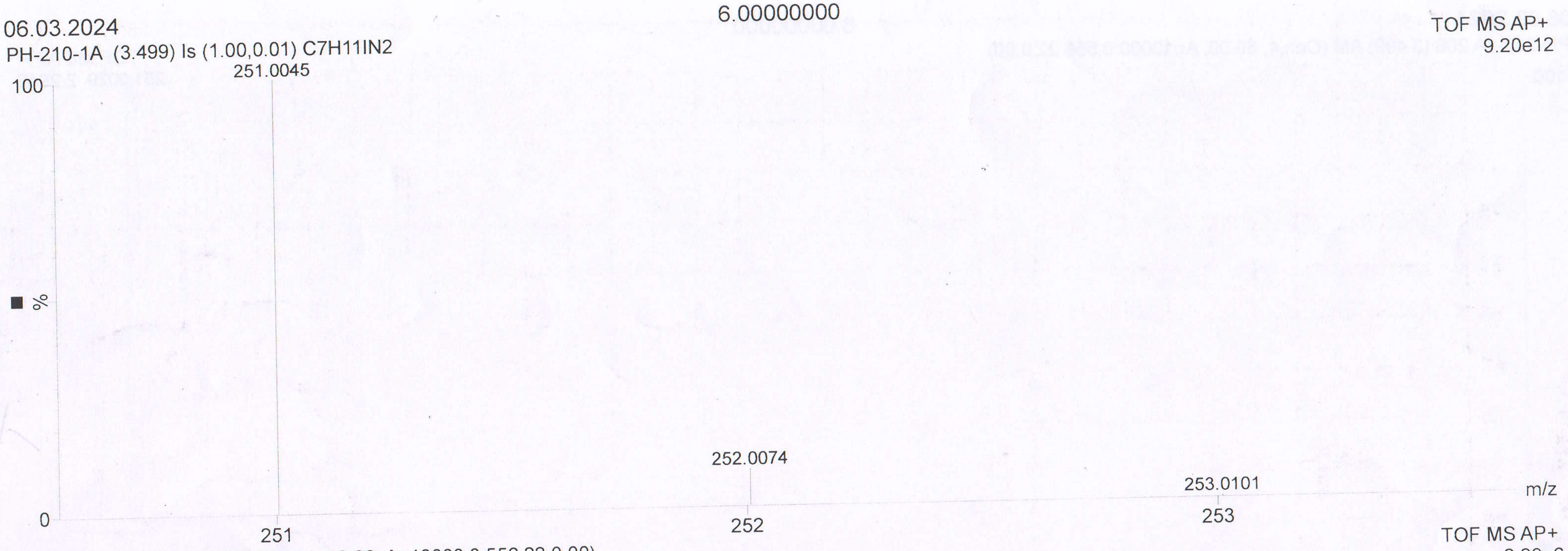

06.03.2024
6.00000000
PH-210-1A (3.499) Is (1.00,0.01) C7H11IN2
TOF MS AP+
9.20e12
251.0045
252.0074
253.0101
100
%
0
251
252
253
m/z

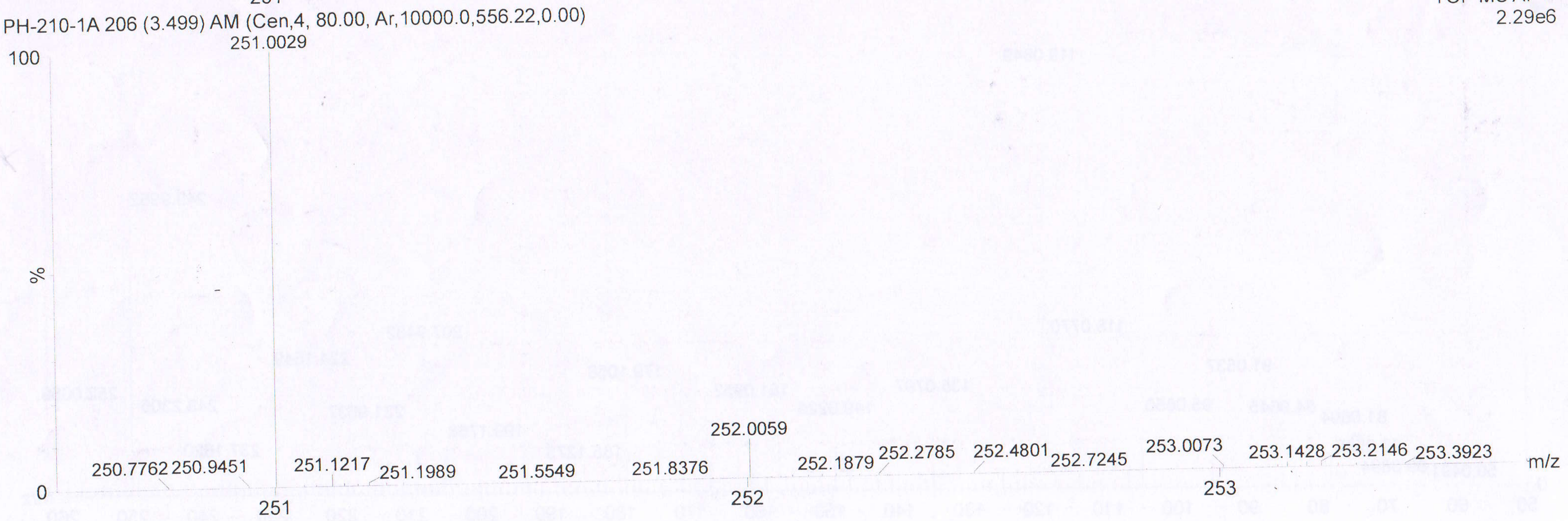

PH-210-1A 206 (3.499) AM (Cen,4, 80.00, Ar,10000.0,556.22,0.00)
TOF MS AP+
2.29e6
251.0029
100
%
0
250.7762
250.9451
251.1217
251.1989
251.5549
251.8376
252.0059
252.1879
252.2785
252.4801
252.7245
253.0073
253.1428
253.2146
253.3923
251
252
253
m/z

7d

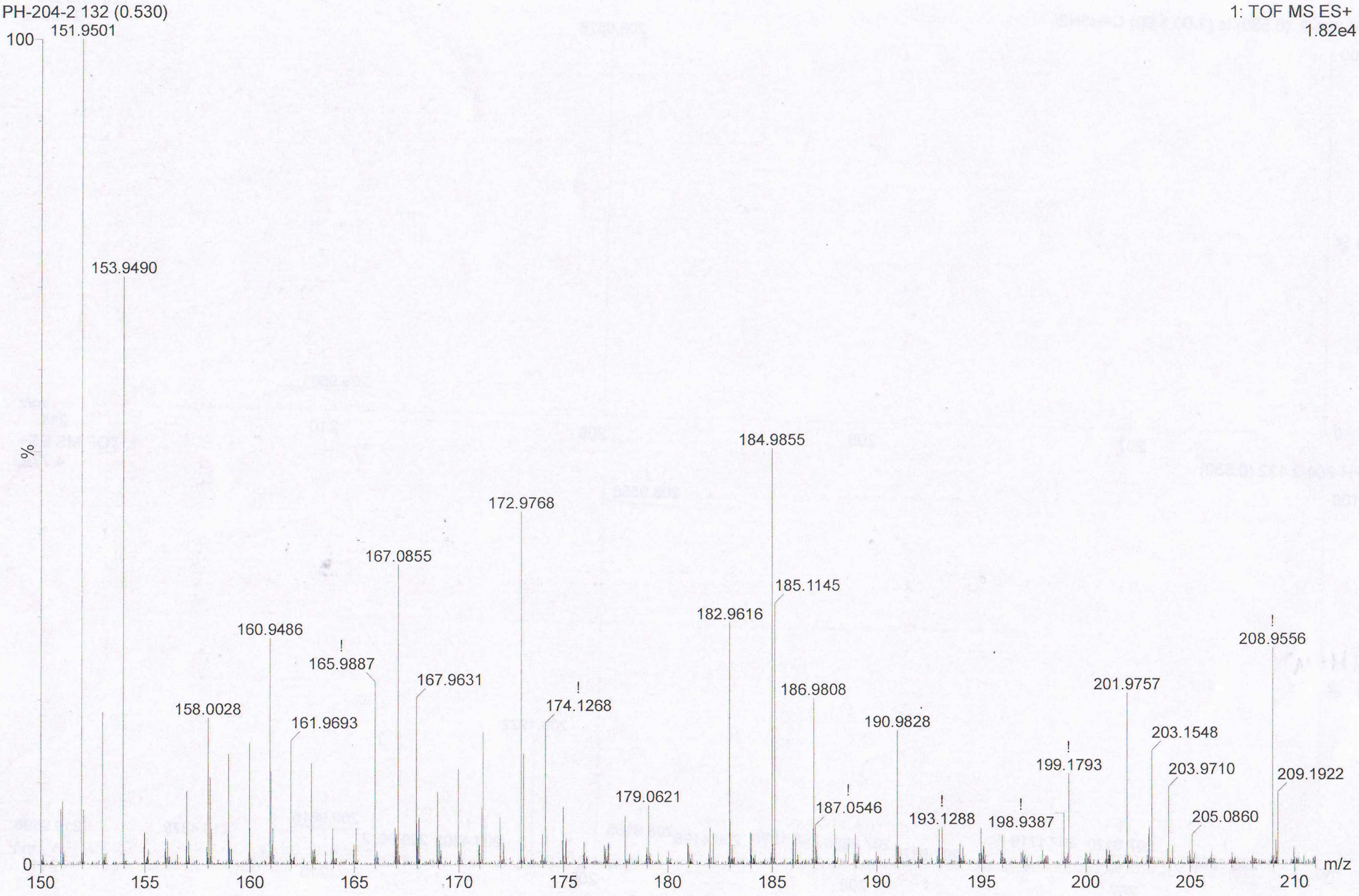
PH-204-2 132 (0.530)
1: TOF MS ES+
1.82e4
100
%
0
151.9501
153.9490
158.0028
160.9486
161.9693
165.9887
167.0855
167.9631
172.9768
174.1268
179.0621
182.9616
184.9855
185.1145
186.9808
187.0546
190.9828
193.1288
198.9387
199.1793
201.9757
203.1548
203.9710
205.0860
208.9556
209.1922
150
155
160
165
170
175
180
185
190
195
200
205
210
m/z

7d

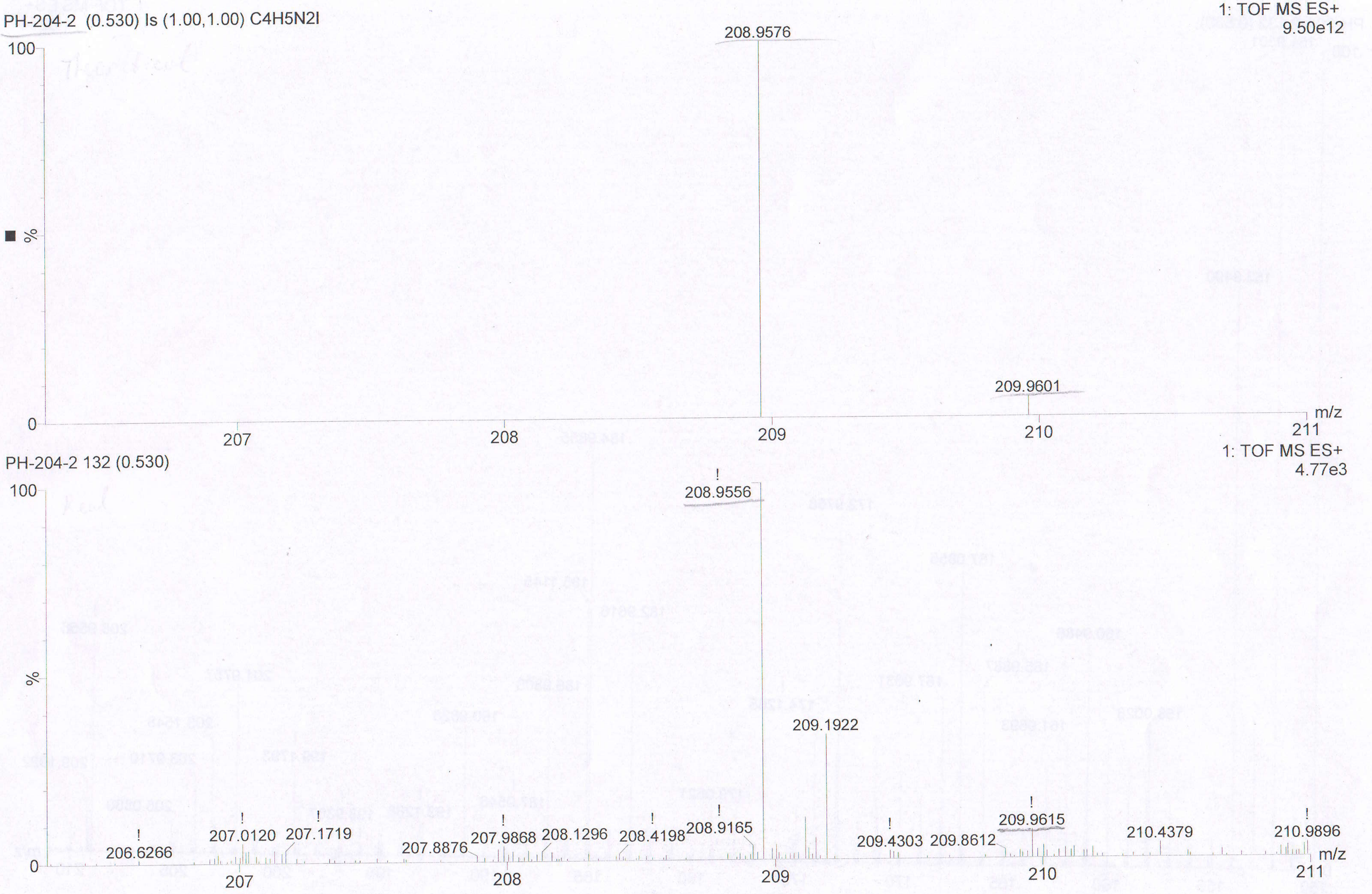
PH-204-2 (0.530) Is (1.00,1.00) C4H5N2I
1: TOF MS ES+
9.50e12
100
%
0
208.9576
209.9601
207
208
209
210
211
m/z
PH-204-2 132 (0.530)
1: TOF MS ES+
4.77e3
208.9556
209.1922
206.6266
207.0120
207.1719
207.8876
207.9868
208.1296
208.4198
208.9165
209.4303
209.8612
209.9615
210.4379
210.9896

7e

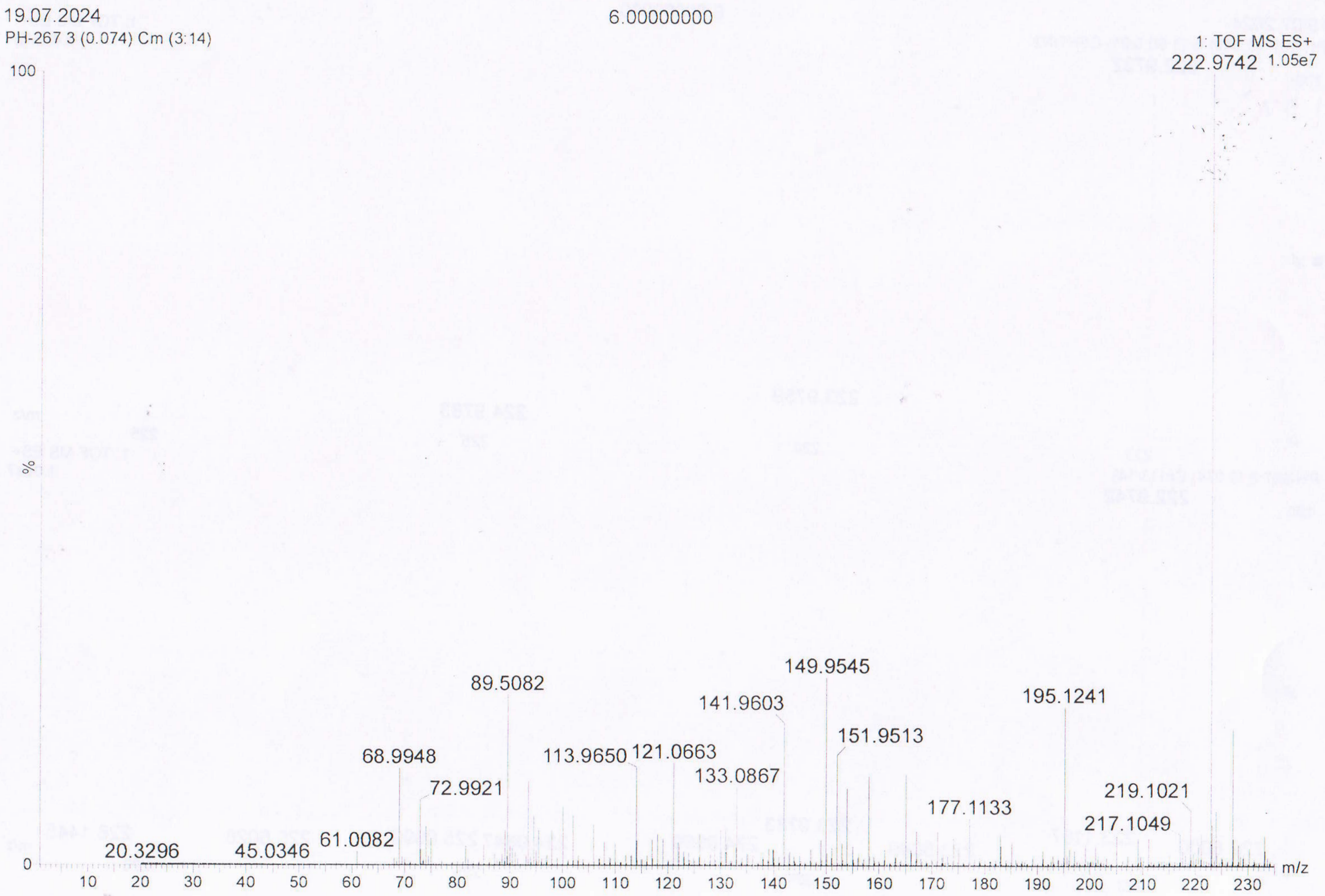
19.07.2024
PH-267 3 (0.074) Cm (3:14)
6.00000000
1: TOF MS ES+
1.05e7
222.9742
100
%
0
149.9545
89.5082
141.9603
195.1241
151.9513
68.9948
113.9650
121.0663
133.0867
72.9921
219.1021
177.1133
217.1049
61.0082
20.3296
45.0346
m/z
10
20
30
40
50
60
70
80
90
100
110
120
130
140
150
160
170
180
190
200
210
220
230

7e

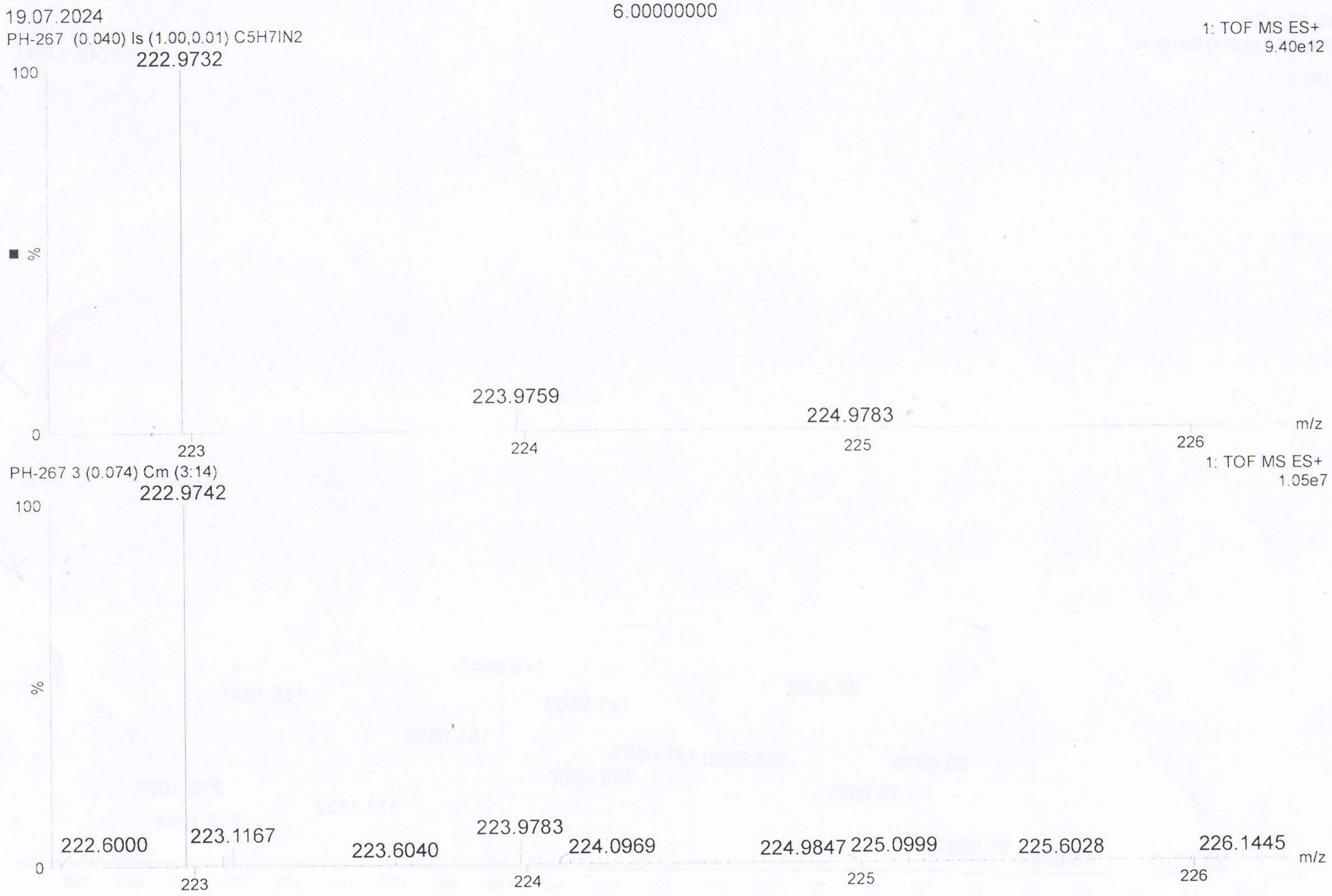

19.07.2024
6.00000000
PH-267 (0.040) Is (1.00,0.01) C5H7IN2
1: TOF MS ES+
9.40e12
222.9732
223.9759
224.9783
100
%
0
223
224
225
226
m/z
PH-267 3 (0.074) Cm (3:14)
1: TOF MS ES+
1.05e7
222.9742
222.6000
223.1167
223.6040
223.9783
224.0969
224.9847
225.0999
225.6028
226.1445

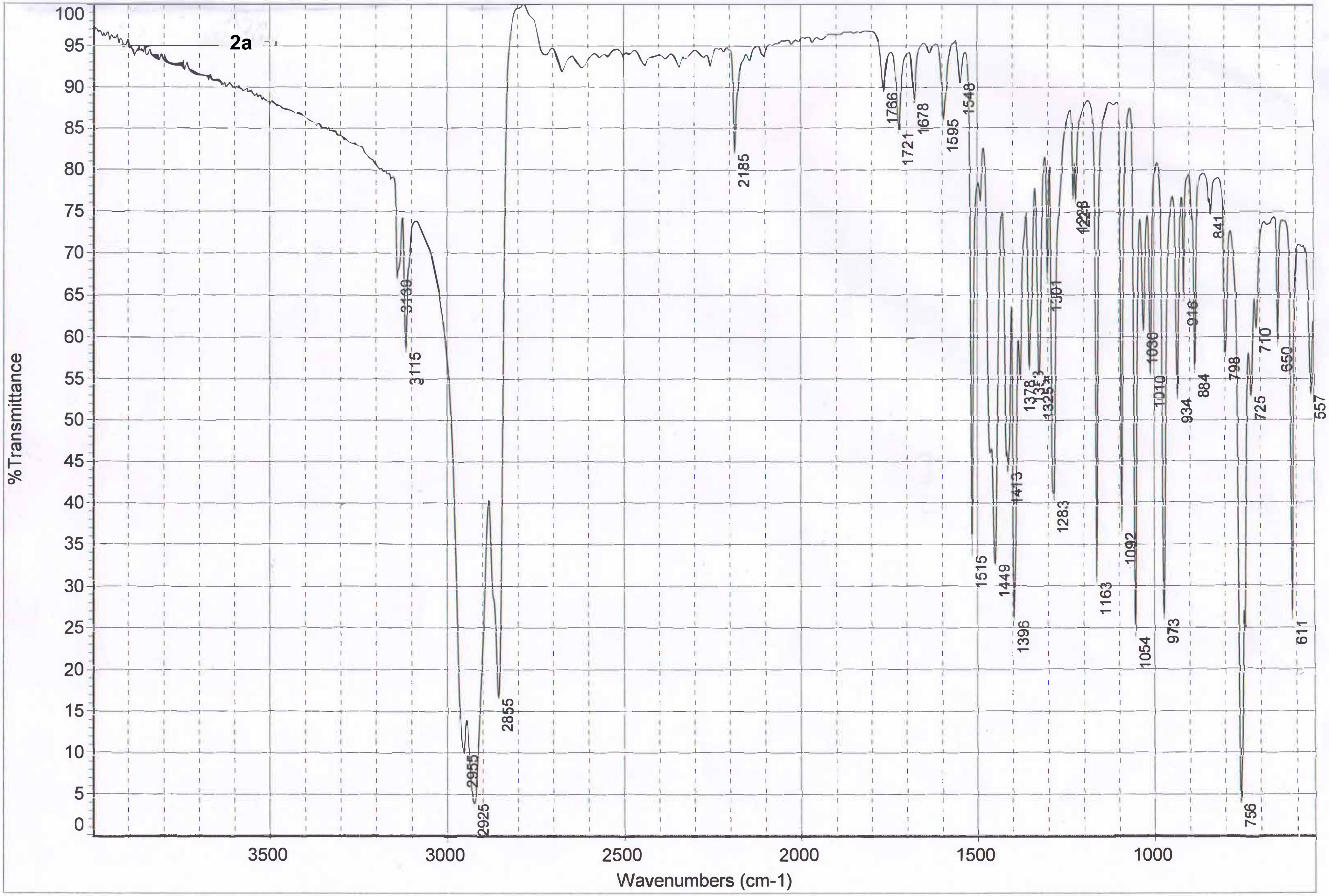
2a
%Transmittance
Wavenumbers (cm-1)
3500
3000
2500
2000
1500
1000
3139
3115
2955
2925
2855
2185
1766
1721
1678
1595
1548
1515
1449
1413
1396
1378
1325
1301
1283
1228
1163
1092
1054
1036
1010
973
934
916
884
841
798
756
725
710
650
611
557

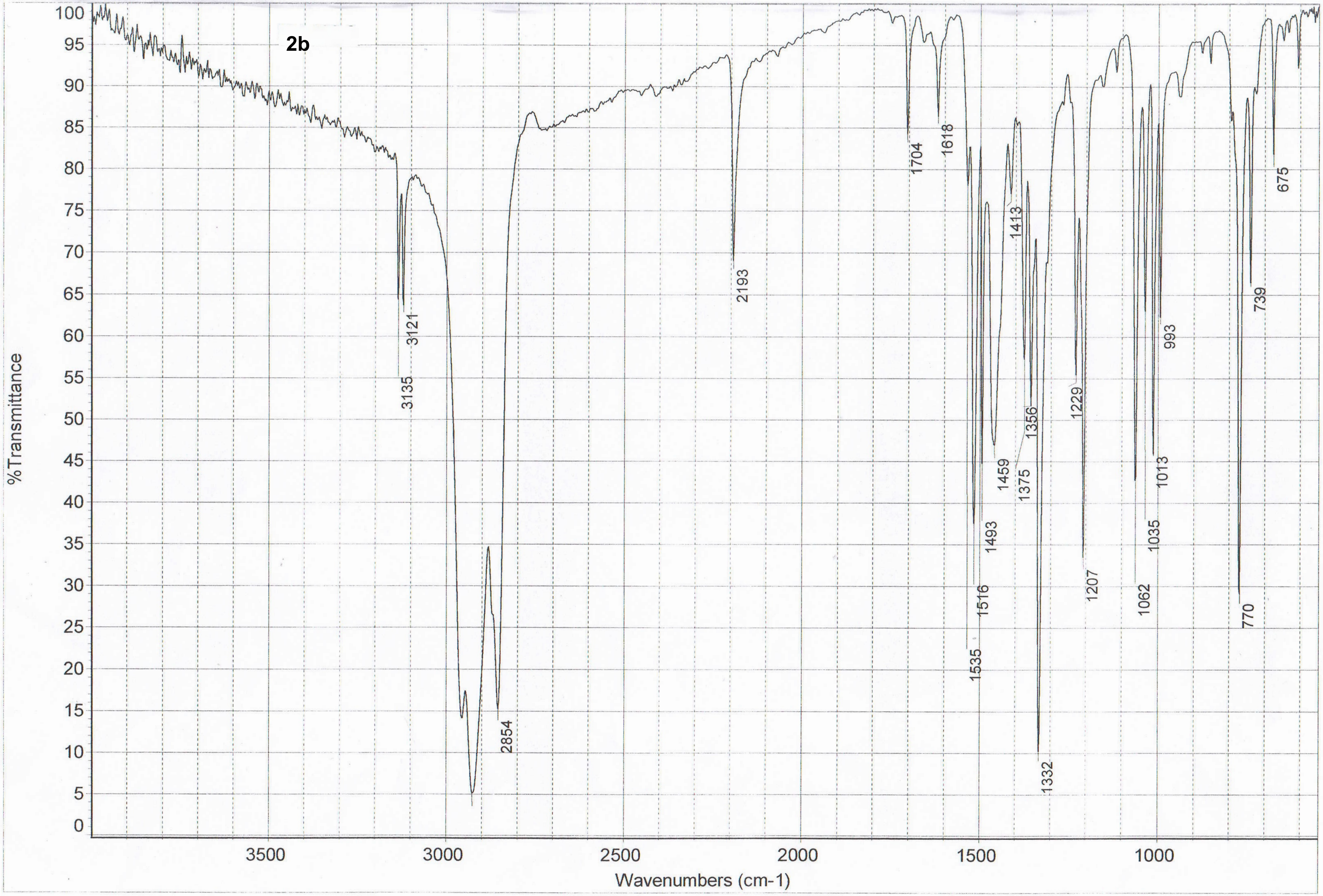

2b
%Transmittance
Wavenumbers (cm-1)
3135
3121
2854
2193
1704
1618
1535
1516
1493
1459
1413
1375
1356
1332
1229
1207
1062
1035
1013
993
770
739
675

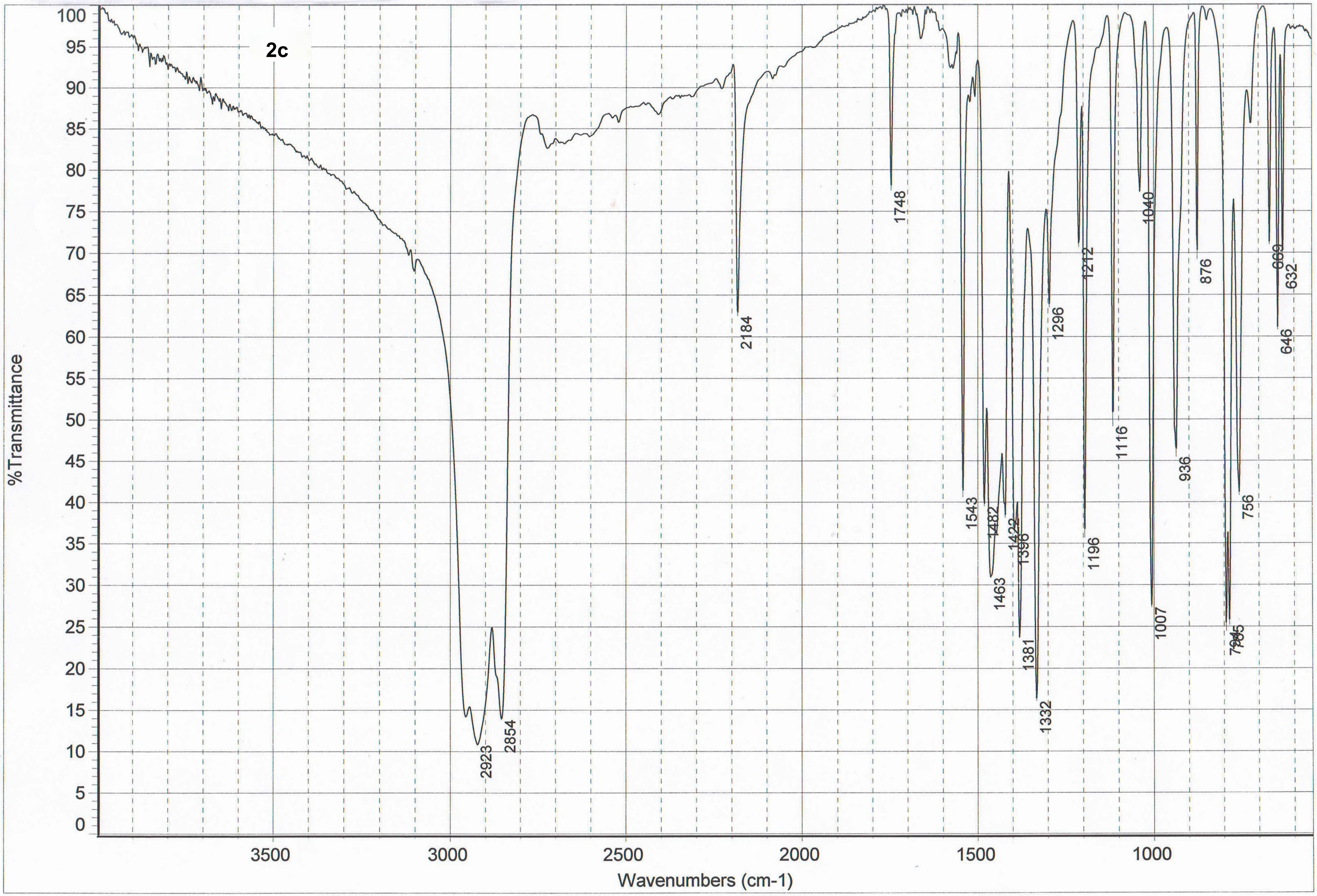
2c
%Transmittance
Wavenumbers (cm-1)
2923
2854
2184
1748
1543
1482
1463
1422
1396
1381
1332
1296
1212
1196
1116
1040
1007
936
876
756
669
646
632

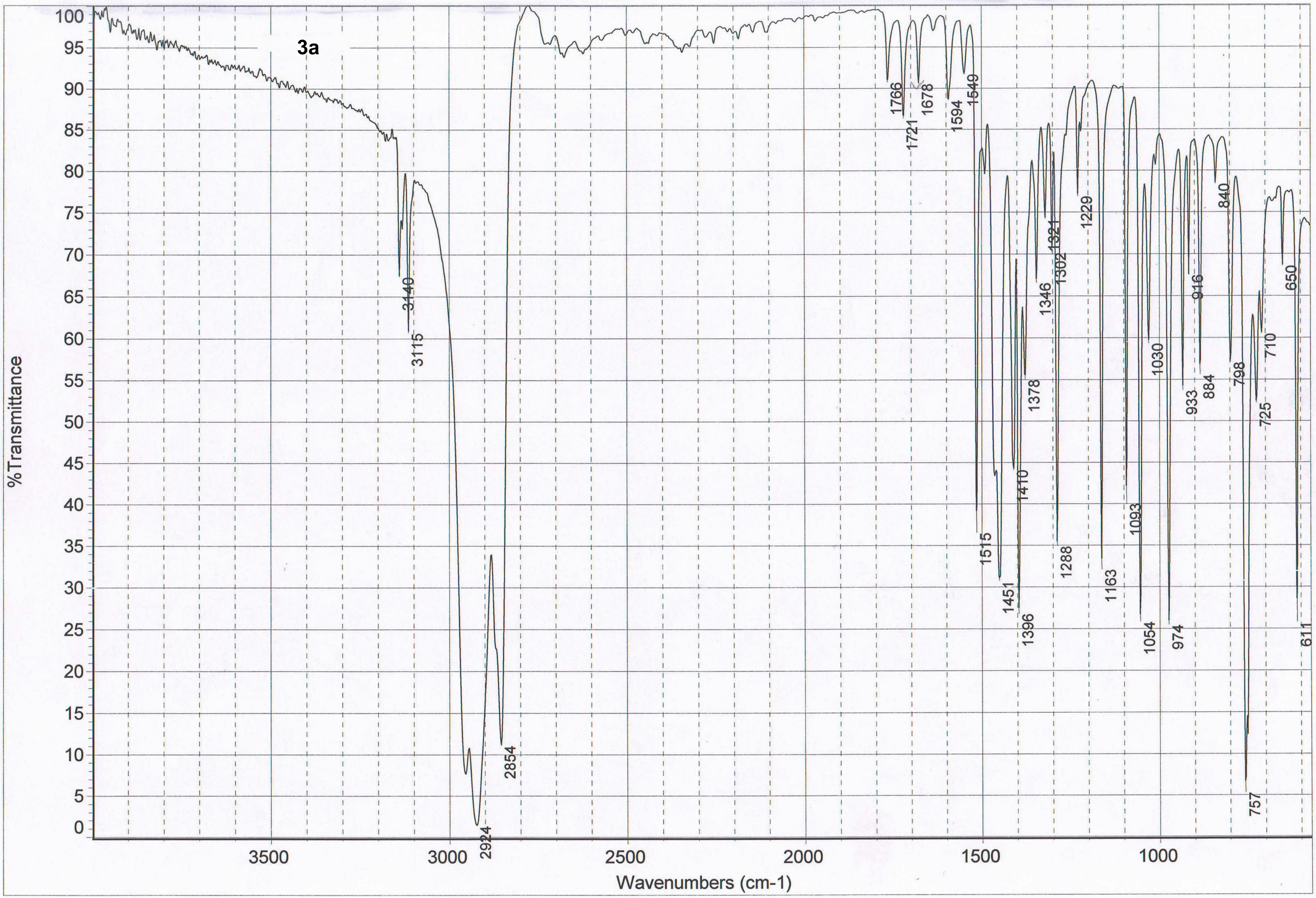
3a
%Transmittance
Wavenumbers (cm-1)
100
95
90
85
80
75
70
65
60
55
50
45
40
35
30
25
20
15
10
5
0
3500
3000
2500
2000
1500
1000
3140
3115
2924
2854
1766
1721
1678
1594
1549
1515
1451
1410
1396
1378
1346
1321
1302
1288
1229
1163
1093
1054
1030
974
933
916
884
840
798
757
725
710
650
611

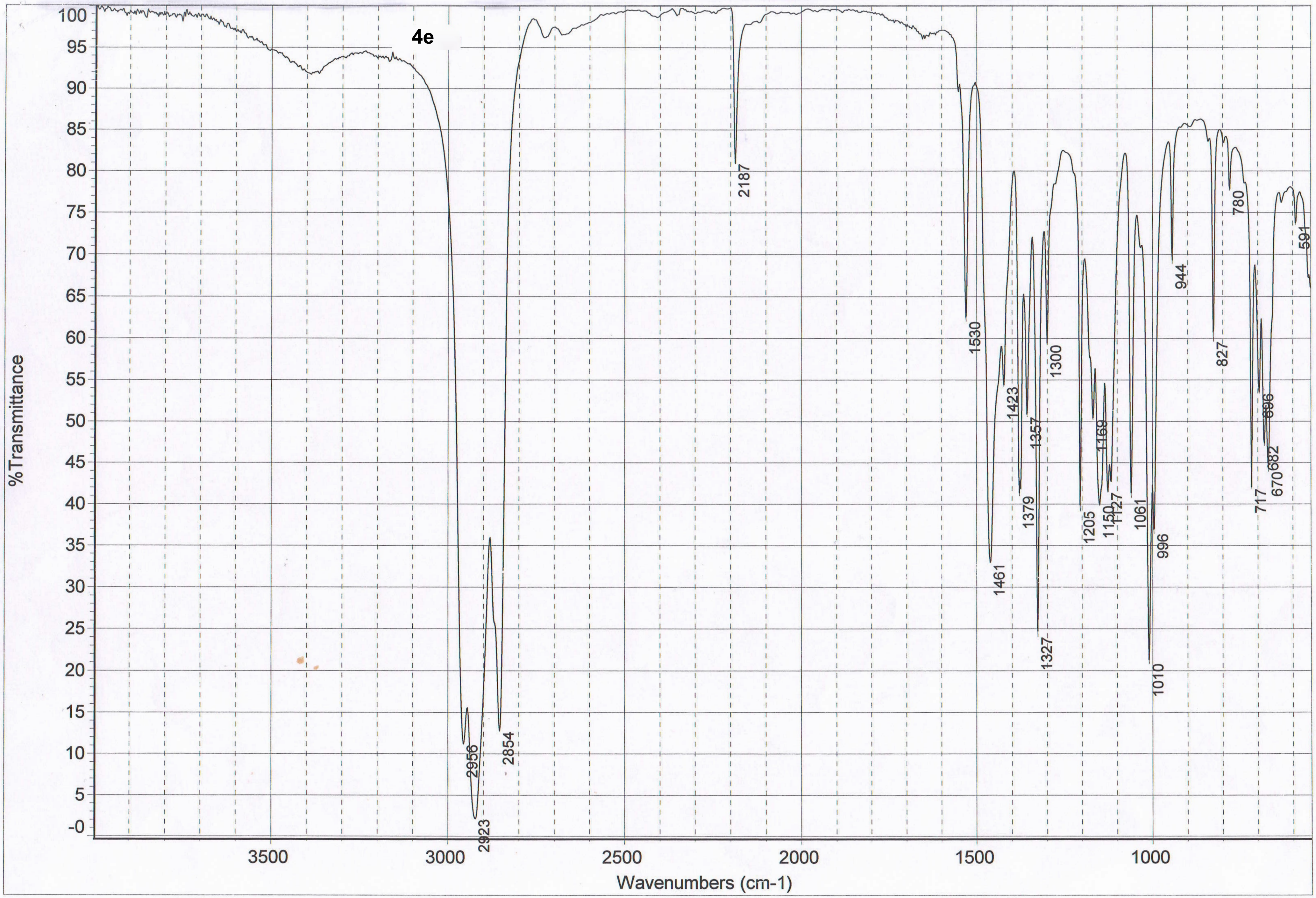
4e
%Transmittance
Wavenumbers (cm-1)
2956
2923
2854
2187
1530
1461
1423
1379
1357
1327
1300
1205
1169
1150
1127
1061
1010
996
944
827
780
717
696
682
670
591

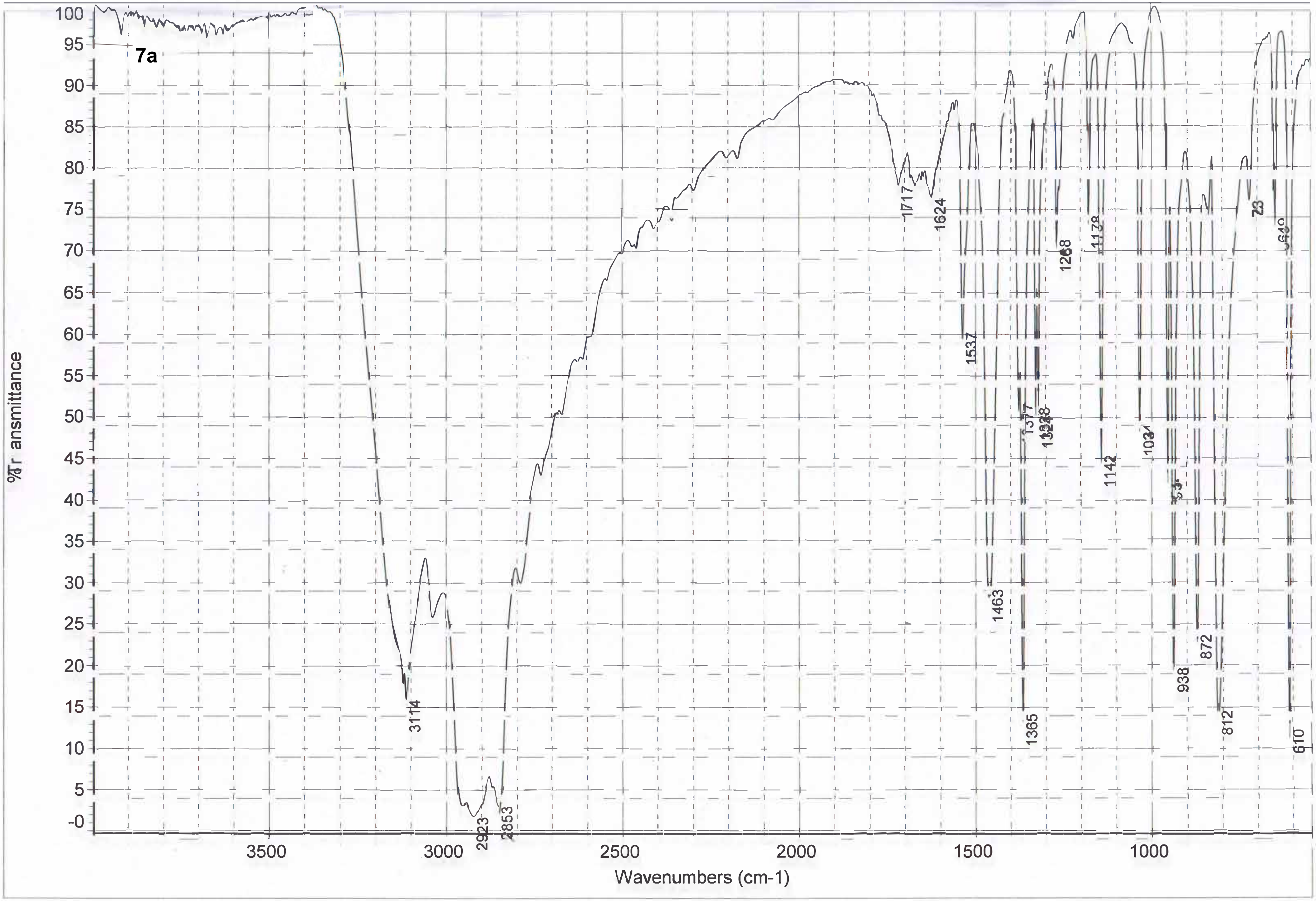

7a
%Transmittance
Wavenumbers (cm-1)
3114
2923
2853
1717
1624
1537
1463
1377
1365
1328
1268
1178
1142
1034
938
872
812
610

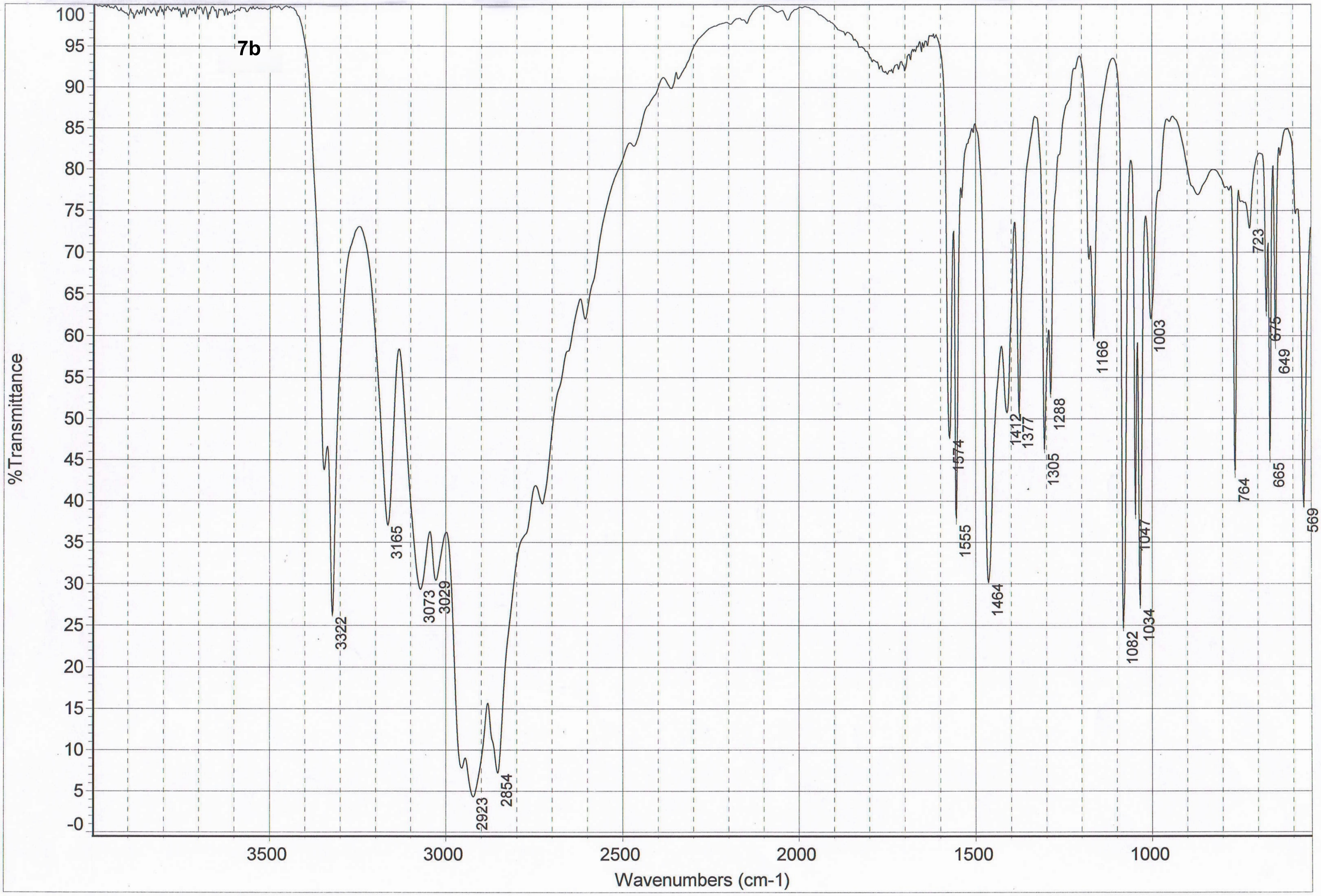
7b
%Transmittance
Wavenumbers (cm-1)
3322
3165
3073
3029
2923
2854
1574
1555
1464
1412
1377
1305
1288
1166
1082
1047
1034
1003
764
723
675
665
649
569

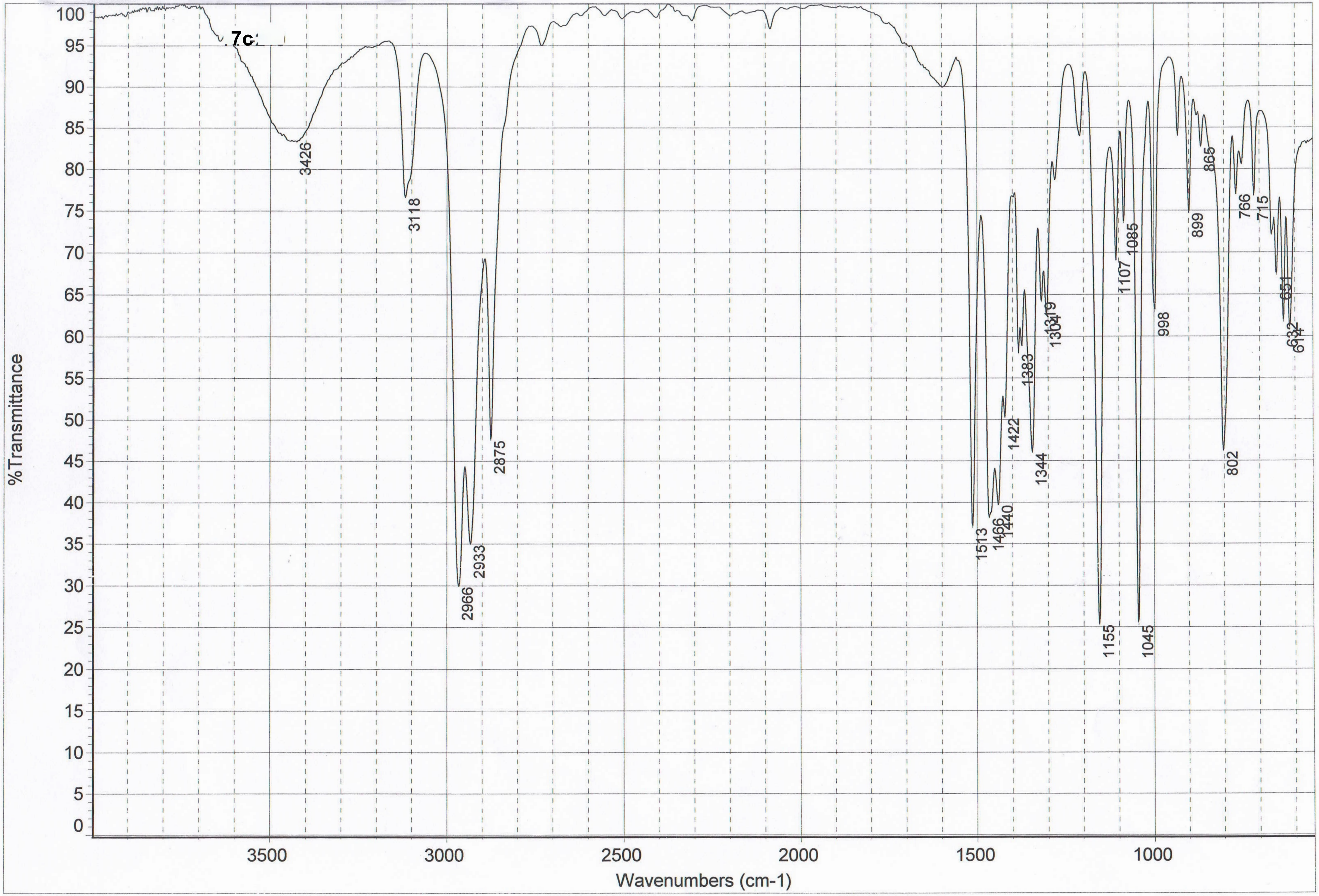
7c
%Transmittance
Wavenumbers (cm-1)
3426
3118
2966
2933
2875
1513
1466
1440
1422
1383
1344
1319
1304
1155
1107
1085
1045
998
899
865
802
766
715
651
632
614

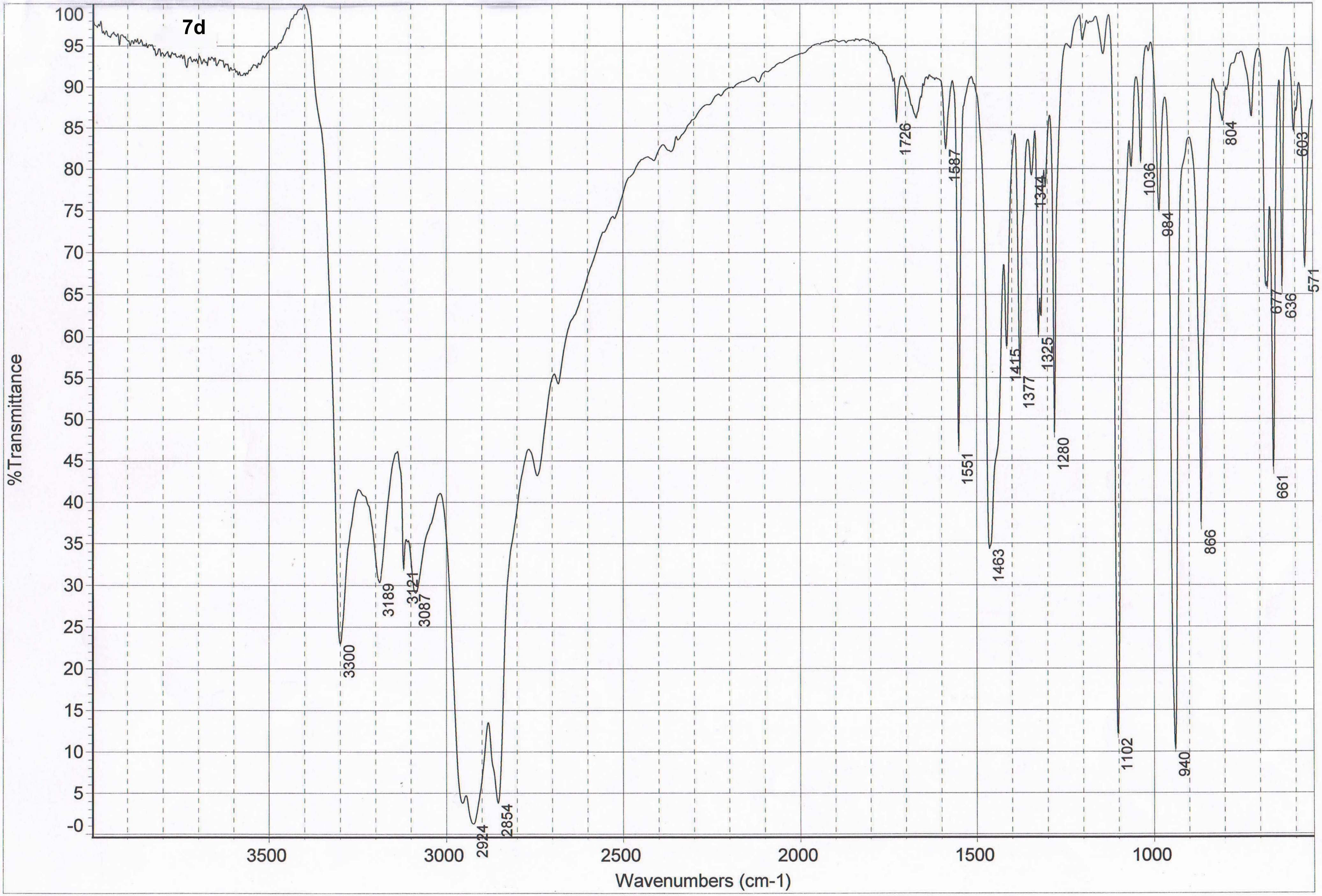

7d
%Transmittance
Wavenumbers (cm-1)
3300
3189
3121
3087
2924
2854
1726
1587
1551
1463
1415
1377
1344
1325
1280
1102
1036
984
940
866
804
677
661
636
603
571

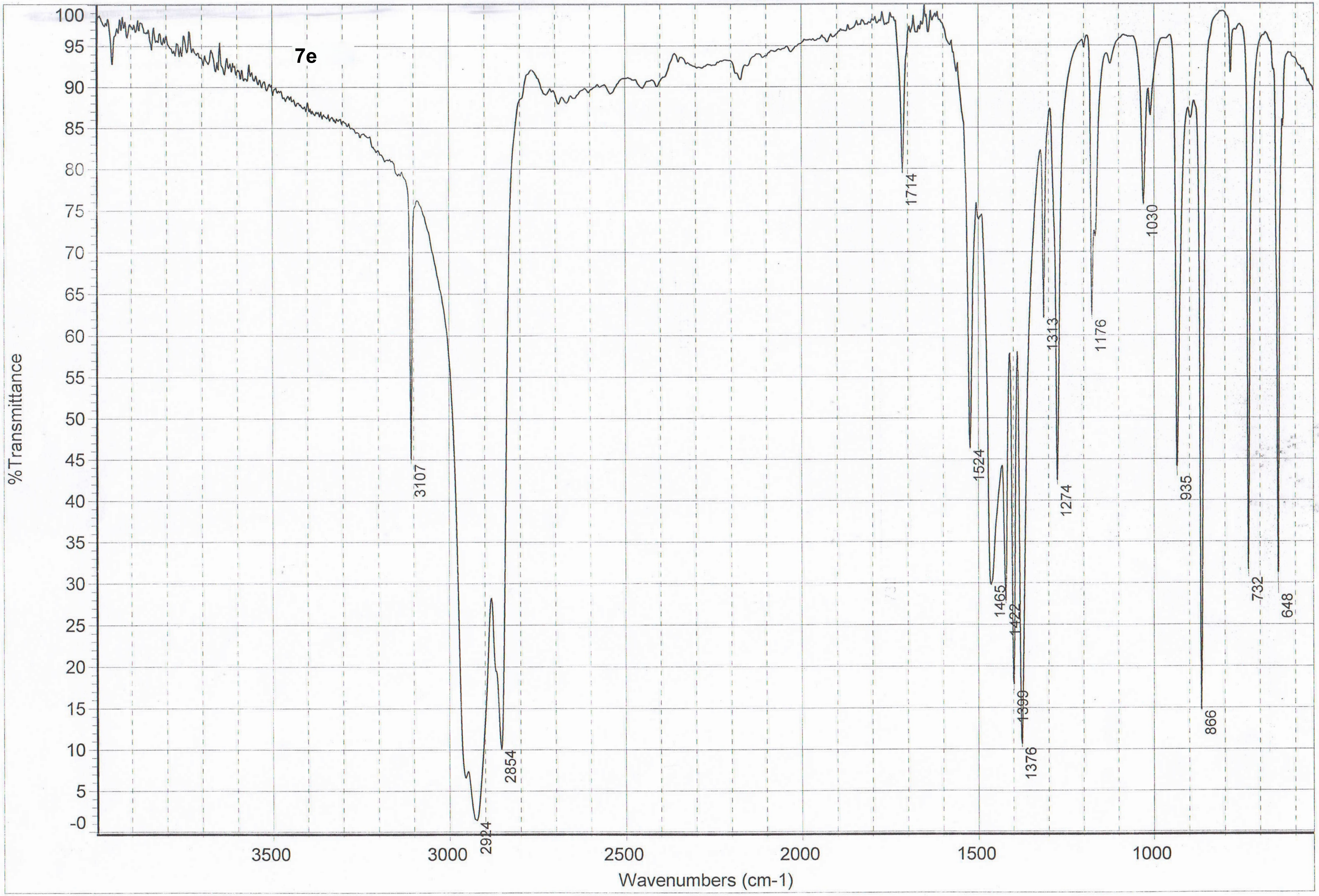
7e
%Transmittance
Wavenumbers (cm-1)
3107
2924
2854
1714
1524
1465
1422
1399
1376
1313
1274
1176
1030
935
866
732
648

X-ray Crystallographic Data

The diffraction measurements of crystals of compounds **2a, 2b, 2e** were carried out at temperature of 100K on a XtaLAB Synergy-S diffractometer using Mo-Kα radiation, ω-scans rotation. A multi-scan absorption correction for all samples was performed using CrysAlisPro 1.171.43.92a (Rigaku Oxford Diffraction, 2023) using spherical harmonics, implemented in SCALE3 ABSPACK scaling algorithm. The structures were solved by direct method and refined using the software package SHELXTL.[23] In all structural models, all hydrogen atoms were positioned geometrically and refined using riding model. All non-hydrogen atoms were refined anisotropically by full-matrix least squares methods. Crystallographic and experimental data and depositions numbers are listed in **Table S1**.

Table S1

Crystallographic and experimental data

Crystal Data			
Compound	**2a**	**2b**	**2e**
Depositions Number	2383957	2383965	2383967
Formula	$C_6H_5N_2I$	$C_7H_7N_2I$	$C_8H_9N_2I$
Formula Weight	232.02	246.05	260.07
Crystal System	Orthorhombic	Orthorhombic	Monoclinic
Space group	$P2_12_12_1$	Pnma	C_2/c
a, b, c [Å]	4.28307(5), 10.98852(13), 15.14434(18)	15.816(3), 6.8466(14), 7.6508(15)	13.7133(4), 9.9569(2), 15.0160(5)
α, β, γ [deg.]	90, 90, 90	90, 90, 90	90, 116.836(4), 90
V [$Å^3$]	712.762(15)	828.5(3)	1829.50(11)
Z	4	4	8
D(calc) [g/cm^3]	2.162	1.973	1.888
μ(MoKα) [mm^{-1}], T_{min}, T_{max}	4.401, 0.55256, 1.00000	3.792, 0.53350, 1.00000	3.440, 0.16100, 0.19119
F(000)	432	464	992
Crystal Size [mm]	0.38×0.20×0.18	0.38×0.20×0.08	0.34×0.23×0.17
Data Collection			
Temperature (K)	100	100	100
Radiation [Å]	MoKα 0.71073	MoKα 0.71073	MoKα 0.71073
θ_{min}, θ_{max} [Deg]	2.3, 32.2	2.6, 33.6	2.6, 33.2
Dataset	-6≤h≤6; -16≤k≤15; -22≤l≤22	-23≤h≤24; -10≤k≤10; -11≤l≤11	-19≤h≤15; -14≤k≤14; -16≤l≤22
Tot., Uniq. Data, R(int)	20130, 2402, 0.044	48692, 1689, 0.108	9587, 3094, 0.019
Observed data [I > 2.0 σ(I)]	2374	1557	2954
Refinement			
Nref, Npar	2402, 103	1689, 62	3094, 102

R, wR2, S	0.0160, 0.0360, 1.09	0.0251, 0.0639, 1.11	0.0285, 0.0767, 1.08

The molecular structure of the compounds **2a, 2b** and **2e** are shown in **Fig. S1, S2** and **S3** respectively.

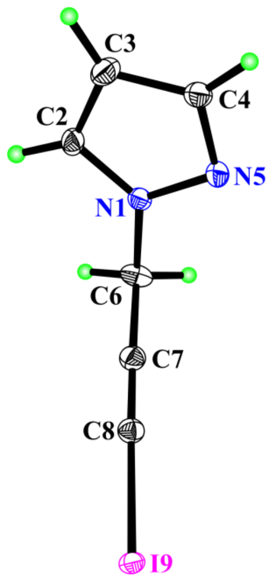

Fig. S1. The molecular structure of compound **2a** with thermal displacement ellipsoids drawn at the 50% probability level.

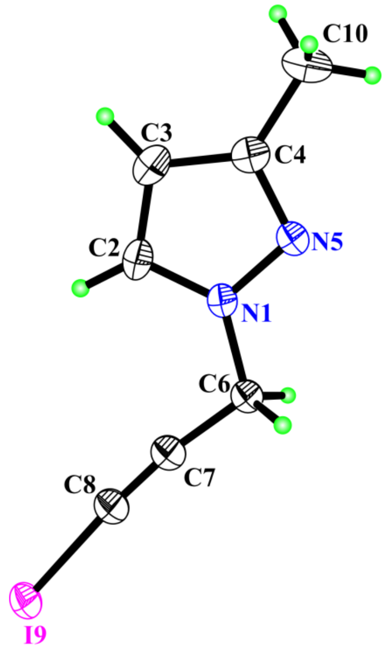

Fig. S2. The molecular structure of compound **2b** with thermal displacement ellipsoids drawn at the 50% probability level.

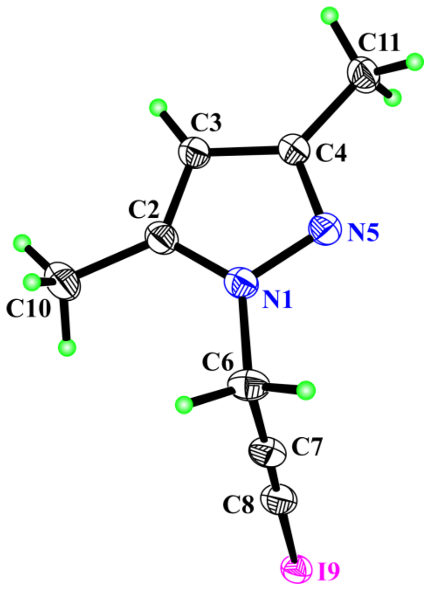

Fig. S3. The molecular structure of compound **2e** with thermal displacement ellipsoids drawn at the 50% probability level.
